# Supplementary material for: Mixed methods study to develop the content validity and the conceptual framework of the electronic patient-reported outcome measure for vascular conditions
Source: BMJ Open. 2020 Aug 11;10(8):e034154. doi: 10.1136/bmjopen-2019-034154 (PMC7418769; doi:10.1136/bmjopen-2019-034154)
Supplement: Supplementary data [file bmjopen-2019-034154supp001.pdf]

Supplementary material

S 1

Table 1- Appraisal criteria for assessing the psychometric properties of patient-reported outcome measures

Psychometric property domains	Subdomain	Thresholds
Reliability	Test–retest reliability	Intraclass correlation/weighted κ ≥ 0·70 for group comparisons<br>Intraclass correlation/weighted κ ≥ 0·90 for individual scores<br>Evidence of mean difference between time point 1 and time point 2, reported with 95 per cent c.i. (using paired *t* test or Wilcoxon signed-rank test)
	Internal consistency	Cronbach's α score of ≥ 0·70 indicates good evidence, but the score should not exceed ≥ 0·92 for group comparison<br>Item total correlations should be ≥ 0·20
Validity	Content validity	Evidence that the instrument has been developed by undertaking a literature review, consulting patients, clinicians and other experts

Psychometric property domains	Subdomain	Thresholds
	Construct validity	Correlation coefficient of ≥ 0·60 indicates strong evidence<br>This should be supported by specific directional hypotheses and a previous estimation of strength of correlation.
	Criterion validity	Justification for selection of standard should be adequate<br>Correlation between PROM and standard ≥ 0·70
Responsiveness	Responsiveness	Statistically significant changes in score of an expected magnitude based on methods including *t* tests, effect size, standardized response means, Guyatt's responsiveness index or responsiveness statistics
Acceptability	Floor/ceiling effects	Evidence of floor effect: 15 per cent of respondents are achieving the lowest score on the instrument<br>Evidence of ceiling effect: 15 per cent of respondents are achieving the highest score on the instrument
	Acceptability	Completeness of data supplied ≥ 80 per cent

S 2

AAA search strategies: search one
Database: Ovid MEDLINE(R) In-Process & Other Non-Indexed Citations and Ovid MEDLINE(R) <1946 to Present>
Search Strategy:
----
1. (Aortic aneurysm$ or triple A or true aneurysm$).tw.
2. Aortic Aneurysm/
3. Aortic Aneurysm, Abdominal/
4. 1 or 2 or 3
5. (patient reported outcome$ or patient-reported outcome$).tw.
6. (prom or proms).tw.
7. (disease reported outcome$ or disease-reported outcome$).tw.
8. 5 or 6 or 7
9. "Quality of Life"/
10. "Outcome and Process Assessment (Health Care)"/
11. "Outcome Assessment (Health Care)"/
12. quality of life.tw.
13. qol.tw.
14. outcome measure$.tw.
15. health outcome$.tw.
16. or/9-15
17. (patient adj20 report$).tw.
18. 16 and 17
19. 8 or 18
20. nottingham health profile.tw.
21. health related quality of life.tw.
22. health related qol.tw.
23. health related ql.tw.
24. hrqol.tw.
25. hql.tw.
26. health state utilit$.tw.
27. hsuv$.tw.

28. (euroqol or euro qol or eq5d or eq 5d).tw.
29. (sf6d or sf 6d or sf 6 dimension$ or sf six dimension$ or shortform 6d or shortform six dimension$ or short form 6d or short form 6 dimension$ or short form six dimension$).tw.
30. (sf12 or sf 12 or short form 12 or shortform 12 or sf twelve or sftwelve or shortform twelve or short form twelve).tw.
31. (sf36 or sf 36 or short form 36 or shortform 36 or sf thirtysix or sf thirty six or shortform thirtysix or shortform thirty six or short form thirtysix or short form thirty six).tw.
32. (item adj3 short form).tw.
33. (item adj3 shortform).tw.
34. medical outcomes survey.tw.
35. medical outcomes study.tw.
36. mos.tw.
37. psychological general wellbeing index.tw.
38. psychological general well being index.tw.
39. pgwb$.tw.
40. health utilit$.tw.
41. hui$.tw.
42. quality of wellbeing.tw.
43. quality of well being.tw.
44. qwb$.tw.
45. rosser.tw.
46. trade off$.tw.
47. standard gamble$.tw.
48. tto$.tw.
49. qaly$.tw.
50. quality adjusted life year$.tw.
51. quality-adjusted life years/
52. hye$.tw.
53. health$ year$ equivalent$.tw.
54. disutilit$.tw.
55. disbenefit$.tw.
56. "Quality of Life"/
57. "Outcome Assessment (Health Care)"/
58. quality of life.tw.
59. 56 or 57 or 58
60. (preference based or utilit$ or generic preference).tw.
61. 59 and 60
62. (preference$ adj2 (elicit$ or patient$ or population$ or measure$ or based or cost$)).tw.
63. (utilit$ adj2 (elicit$ or patient$ or population$ or measure$ or based or cost$)).tw.
64. or/20-55

65. 61 or 62 or 63 or 64
66. 19 or 65
67. 4 and 66

AAA search strategies: search two
Database: Ovid MEDLINE(R) In-Process & Other Non-Indexed Citations and Ovid MEDLINE(R) <1946 to Present>
Search Strategy:
----
1. (Aortic aneurysm$ or triple A or true aneurysm$).tw.
2. Aortic Aneurysm/
3. Aortic Aneurysm, Abdominal/
4. or/1-3
5. time trade off.mp.
6. Watt index.mp.
7. (Rand-36 or Rand36 or Rand 36).mp.
8. self-assessed health.mp.
9. screenQL.mp.
10. general health questionnaire.mp.
11. female sexual function index.mp.
12. Rose questionnaire.mp.
13. Australian Vascular Quality of Life Index.mp.
14. AUSVIQUOL.mp.
15. Nottingham Health Profile.mp.
16. NHP.mp.
17. (sf36 or sf 36 or short form 36 or shortform 36 or sf thirtysix or sf thirty six or shortform thirtysix or shortform thirty six or short form thirtysix or short form thirty six).mp. [mp=title, abstract, original title, name of substance word, subject heading word, keyword heading word, protocol supplementary concept word, rare disease supplementary concept word, unique identifier]
18. (sf12 or sf 12 or short form 12 or shortform 12 or sf twelve or sftwelve or shortform twelve or short form twelve).mp. [mp=title, abstract, original title, name of substance word, subject heading word, keyword heading word, protocol supplementary concept word, rare disease supplementary concept word, unique identifier]
19. (sf20 or sf 20 or short form 20 or shortform 20 or sf twenty or sftwenty or shortform twenty or short form twenty).mp. [mp=title, abstract, original title, name of substance word, subject heading word, keyword heading word, protocol supplementary concept word, rare disease supplementary concept word, unique identifier]
20. (Hospital Anxiety and Depression Scale).mp. [mp=title, abstract, original title, name of substance word, subject heading word, keyword heading word, protocol supplementary concept word, rare disease supplementary concept word, unique identifier]
21. HADS.mp.
22. Standard gamble$.mp. [mp=title, abstract, original title, name of substance word, subject heading word, keyword heading word, protocol supplementary concept word, rare disease supplementary concept word, unique identifier]
23. rosser.tw.
24. (euroqol or euro qol or eq5d or eq 5d or eq-5d or eq-5d VAS).mp. [mp=title, abstract, original title, name of substance word, subject heading word, keyword heading word, protocol supplementary concept word, rare disease supplementary concept word, unique identifier]

25. World Health Organization Quality of Life-BREF.mp. [mp=title, abstract, original title, name of substance word, subject heading word, keyword heading word, protocol supplementary concept word, rare disease supplementary concept word, unique identifier]
26. WHOQOL-BREF.mp. [mp=title, abstract, original title, name of substance word, subject heading word, keyword heading word, protocol supplementary concept word, rare disease supplementary concept word, unique identifier]
27. or/5-26
28. instrumentation.sh.
29. methods.sh.
30. Validation Studies.pt.
31. Comparative Study.pt.
32. Psychometrics/
33. psychometr*.ti,ab.
34. clinimetr*.tw.
35. clinometr*.tw.
36. "Outcome Assessment (Health Care)"/
37. outcome assessment.ti,ab.
38. outcome measure*.tw.
39. Observer Variation/
40. observer variation.ti,ab.
41. Health Status Indicators/
42. "Reproducibility of Results"/
43. reproducib*.ti,ab.
44. Discriminant Analysis/
45. reliab*.ti,ab.
46. unreliab*.ti,ab.
47. valid*.ti,ab.
48. coefficient.ti,ab.
49. homogeneity.ti,ab.
50. homogeneous.ti,ab.
51. "internal consistency".ti,ab.
52. 28 or 29 or 30 or 31 or 32 or 33 or 34 or 35 or 36 or 37 or 38 or 39 or 40 or 41 or 42 or 43 or 44 or 45 or 46 or 47 or 48 or 49 or 50 or 51
53. cronbach*.ti,ab.
54. (alpha or alphas).ti,ab.
55. 53 and 54
56. item.ti,ab.
57. (correlation* or selection* or reduction*).ti,ab.
58. 56 and 57
59. (agreement or precision or imprecision or "precise values" or "test–retest").ti,ab.
60. (test and retest).ti,ab.
61. reliab*.ti,ab.

62. (test or retest).ti,ab.
63. 61 and 62
64. 55 or 58 or 59 or 60 or 63
65. (stability or interrater or inter-rater or intrarater or intra-rater or intertester or inter-tester or intratester or intra-tester or interobserver or inter-observer or intraobserver or intra-observer).ti,ab.
66. (intertechnician or inter-technician or intratechnician or intra-technician or interexaminer or inter-examiner or intraexaminer or intra-examiner or interassay or inter-assay or intraassay or intra-assay or interindividual or inter-individual or intraindividual or intra-individual or interparticipant or inter-participant or intraparticipant or intra-participant).ti,ab.
67. (kappa or "kappa’s" or kappas or repeatab*).ti,ab.
68. repeatab*.ti,ab.
69. 65 or 66 or 67 or 68
70. (replicab* or repeated).ti,ab.
71. (measure or measures or findings or result or results or test or tests).ti,ab.
72. 70 and 71
73. (generaliza* or generalisa* or concordance).ti,ab.
74. (intraclass and correlation*).ti,ab.
75. (discriminative or "known group" or factor analysis or factor analyses or dimension* or subscale*).ti,ab.
76. (multitrait and scaling and (analysis or analyses)).ti,ab.
77. 72 or 73 or 74 or 75 or 76
78. (item discriminant or interscale correlation* or error or errors or "individual variability").ti,ab.
79. (variability and (analysis or values)).ti,ab.
80. (uncertainty and (measurement or measuring)).ti,ab.
81. ("standard error of measurement" or sensitiv* or responsive*).ti,ab.
82. ((minimal or minimally or clinical or clinically) and (important or significant or detectable) and (change or difference)).ti,ab.
83. (small* and (real or detectable) and (change or difference)).ti,ab.
84. (meaningful change or "ceiling effect" or "floor effect" or "Item response model" or IRT or Rasch or "Differential item functioning" or DIF or "computer adaptive testing" or "item bank" or "cross-cultural equivalence").ti,ab.
85. 78 or 79 or 80 or 81 or 82 or 83 or 84
86. 52 or 64 or 69 or 77 or 85
87. 4 and 27 and 86

Databases and research registers searched:

- MEDLINE and MEDLINE In Process: Ovid.
- EMBASE: Ovid.
- Cochrane Library
    - Cochrane Database of Systematic Reviews (CDSR)
    - Database of Abstracts of Reviews of Effects (DARE)
    - Cochrane Central Register of Controlled Trials (CCRCT)

    - Health Technology Assessment Database (HTA)
    - NHS Economic Evaluation Database (NHS EED)
- CINAHL
- PROQOLID
- PsychINFO
- Science Citation Index Expanded (SCIE): Web of Science

Varicose veins systematic review search strategy

1 ((Venous or vein) adj2 (disease or stasis or obstruction or incompetence or insufficiency or syndrome or thromboembolism)).tw. (25175)
2 (Venous ulcer$ or leg ulcer$ or venous leg ulcer$).tw. (6632)
3 (Venous insufficiency or Venous incompetence or Venous thromboembolism or VTE or Venous outflow obstruction or VOO or Venous valvular incompetence or VVI).tw. (19687)
4 exp Venous Insufficiency/ (6167)
5 exp Varicose Veins/ (15946)
6 (Venous adj2 (edema or oedema)).tw. (262)
7 Venous disorder$ of the leg$.tw. (16)
8 (Varicose vein$ or spider vein$ or varicosity or varices or telangiectasia$).tw. (28086)
9 Endovenous occlusion.tw. (11)
10 post-thrombotic syndrome.tw. (716)
11 Venous Thrombosis/ or Venous Thromboembolism/ (24327)
12 or/1-11 (82600)
13 (patient reported outcome$ or patient-reported outcome$).tw. (5201)
14 (prom or proms).tw. (1755)
15 (disease reported outcome$ or disease-reported outcome$).tw. (2)
16 13 or 14 or 15 (6667)
17 "Quality of Life"/ (129051)
18 "Outcome and Process Assessment (Health Care)"/ (22708)
19 "Outcome Assessment (Health Care)"/ (52618)
20 quality of life.tw. (169753)
21 qol.tw. (22367)
22 outcome measure$.tw. (161039)
23 health outcome$.tw. (26145)
24 ((evalua$ or assess$ or measure$) adj (function$ or symptom$)).tw. (8832)
25 or/17-24 (451890)
26 (patient adj20 report$).tw. (188387)
27 25 and 26 (14509)
28 16 or 27 (17478)

29 (nottingham health profile or aberdeen varicose vein questionnaire or AVVQ or chronic venous insufficiency questionnaire or CIVIQ or charing cross venous ulceration questionnaire or CXVUQ or Venous Insufficiency Epidemiological Economic Study tool or VEINES-QOL or VEINES-SYM or Venous Clinical Severity Score or VCSS or Deep Vein Thrombosis Leg Symptom Index or DVT-LSI or Ferrans Powers Quality of Life Index or QLI).tw. (1447)
30 health related quality of life.tw. (25867)
31 health related qol.tw. (978)
32 health related ql.tw. (5)
33 hrqol.tw. (8922)
34 hql.tw. (87)
35 health state utilit$.tw. (334)
36 hsuv$.tw. (35)
37 (activities of daily living or ADL).tw. (19652)
38 "Activities of Daily Living"/ (52765)
39 (euroqol or euro qol or eq5d or eq 5d).tw. (5064)
40 (sf8 or sf 8 or short form 8 or shortform 8 or sf eight or shortform eight or short form eight).tw. (310)
41 (sf6d or sf 6d or sf 6 dimension$ or sf six dimension$ or shortform 6d or shortform six dimension$ or short form 6d or short form 6 dimension$ or short form six dimension$).tw. (524)
42 (sf12 or sf 12 or short form 12 or shortform 12 or sf twelve or sftwelve or shortform twelve or short form twelve).tw. (3332)
43 (sf36 or sf 36 or short form 36 or shortform 36 or sf thirtysix or sf thirty six or shortform thirtysix or shortform thirty six or short form thirtysix or short form thirty six).tw. (18047)
44 (item adj3 short form).tw. (3935)
45 (item adj3 shortform).tw. (1)
46 medical outcomes survey.tw. (240)
47 medical outcomes study.tw. (3459)
48 mos.tw. (5442)
49 psychological general wellbeing index.tw. (10)
50 psychological general well being index.tw. (203)
51 pgwb$.tw. (252)
52 health utilit$.tw. (1278)
53 hui$.tw. (2939)
54 quality of wellbeing.tw. (8)
55 quality of well being.tw. (354)
56 qwb$.tw. (221)
57 rosser.tw. (74)
58 trade off$.tw. (12716)
59 standard gamble$.tw. (732)
60 tto$.tw. (1143)
61 qaly$.tw. (6062)
62 quality adjusted life year$.tw. (7209)
63 quality-adjusted life years/ (7816)

64 hye$.tw. (753)
65 health$ year$ equivalent$.tw. (39)
66 disutilit$.tw. (274)
67 disbenefit$.tw. (24)
68 "Quality of Life"/ (129051)
69 "Outcome Assessment (Health Care)"/ (52618)
70 quality of life.tw. (169753)
71 68 or 69 or 70 (262013)
72 (preference based or utilit$ or generic preference).tw. (136252)
73 71 and 72 (7974)
74 (preference$ adj2 (elicit$ or patient$ or population$ or measure$ or based or cost$)).tw. (11249)
75 (utilit$ adj2 (elicit$ or patient$ or population$ or measure$ or based or cost$)).tw. (5889)
76 or/29-67 (136441)
77 73 or 74 or 75 or 76 (150495)
78 28 or 77 (164078)
79 12 and 78 (993)

S3

<u>AAA qualitative literature review</u>
Database: Ovid MEDLINE(R) In-Process & Other Non-Indexed Citations and Ovid MEDLINE(R) <1946 to Present>
Search Strategy:
----
1 (Aortic aneurysm$ or triple A or true aneurysm$).tw.
2 Aortic Aneurysm/
3 Aortic Aneurysm, Abdominal/
4 or/1-3 (38292)
5 *Attitude to Health/
6 *Self Care/
7 *Health Knowledge, Attitudes, Practice/
8 (patient* adj4 (feeling* or emotion* or view* or symptom* or perception* or attribute*)).ti,ab.
9 ("health related quality of life" or "health related qol" or "health related ql" or hrqol or hql or "patient reported outcome*" or "patient-reported outcome*" or prom or proms or "disease reported outcome*").ti,ab.
10 ("quality of life" or "qol" or "outcome measure*" or "health outcome*").ti,ab.
11 *"Quality of Life"/
12 *"Outcome Assessment (Health Care)"/ or *"Outcome and Process Assessment (Health Care)"/
13 5 or 6 or 7 or 8 or 9 or 10 or 11 or 12
14 (qualitative* or findings or interview*).mp.

15 focus groups/ or interviews as topic/
16 exp qualitative research/
17 14 or 15 or 16
18 14 or 15 or 16
19 4 and 13
20 17 and 19

****

Databases and research registers which were searched:

- MEDLINE and MEDLINE In Process: Ovid.
- EMBASE: Ovid.
- CINAHL: EBSCO
- PROQOLID
- PsychINFO
- Science Citation Index Expanded (SCIE): Web of Science
- Social Science Citation Index
- Proquest dissertation and theses

S4

Table 1: Example of triangulation between PAD PROM review, qualitative evidence synthesis and qualitative interview themes

Qualitative Review Themes	Qualitative Interview Themes	EQ-5D	NHP	SF-6D	SF-8	SF-36	AUSVI QoL	ICQ	PADQ OL	SIPic	WIQ	Vascu QOL
PAD Health Outcomes												
**Pain - location**	Positive	+/-	-	-	-	-	+	-	+	+/-	-	+
**Pain - sensation**	Positive	+/-	+	+	+	+	-	+	+	-	+	-
**Pain - severity**	Positive	+	-	-	-	-	-	-	+/-	+	-	+
**Pain on walking**	Positive	+	+	-	-	-	+	+	+	+	+	+
**Pain at rest**	Positive	+	+	-	-	-	+	+	+	-	-	+
**Mobility - walking ability**	Postive	+/-	+	-	-	+	+	+	+	+	+	+
**Mobility - walking distance**	Positive	+/-	+/-	-	-	+	+	+	-	+	+	+
**Mobility - walking speed**	Positive	+/-	-	-	-	-	-	-	-	-	+	-
**Mobility - stair climbing**	Positive	+/-	+/-	-	-	+	-	+	-	+	+	-

**Mobility - walking uphill**	Positive	+/-	-	-	-	-	-	-	-	-	-	-
**Bending/ picking up things**	Negative	+/-	-	-	-	+	-	-	-	-	-	-
**Sleep**	Positive	-	+	-	-	+	+	-	-	-	-	--
**Sleep/ pain**	Silence	-	+	-	-	-	+	-	-	-	-	-
**Fatigue**	Negative	-	+	-	-	+	-	-	-	-	-	+
**Sexual functioning**	Negative	-	+	-	-	-	-	-	-	-	-	-
**Comorbiditi es**	Negative	-	-	-	-	-	-	-	-	-	-	-
**Non-healing wounds**	Positive	-	-	-	-	-	+	-	-	-	-	-
**Symptom progression**	Postive	-	-	-	-	-	-	-	-	-	-	-
**Appearance of leg**	Silence	-	-	-	-	-	+	-	-	-	-	-
**Altered sensations in leg**	Silence	-	-	-	-	-	-	-	-	-	-	-
**Foot temperature**	Silence	-	-	-	-	-	-	-	-	-	-	-
Impact on Physical Functioning												
**Personal care**	Positive	+	+	+/-	+/-	+	+	-	+	+	-	+
**Hobbies**	Positive	+	-	-	-	+/-	-	+	+	+	-	+/-
**Exercise**	Negative	+/-	-	-	-	+/-	-	-	-	-	-	+/-

**Daily activities**	Positive	+/-	+	+	+	+	+	+	+	+	-	+
Impact on Social Functioning												
**Social support**	Positive	-	+	-	-	-	-	-	-	-	-	-
**Social activities**	Positive	-	+	+	+	+	+	+	+	+	-	+
**Role**	Positive	-	+/-	+	+	-	-	+	+	-	-	+/-
**Isolation**	Positive	-	+	+/-	+/-	-	+	-	+	-	-	-
**Independence/ burden**	Positive	-	+	+	-	+/-	-	+	-	-	-	+
Impact on Psychological Function												
**Loss/ grief**	Positive	-	-	-	-	+/-	+	-	+	-	-	
**Unfulfilled desire**	Negative	-	+	-	-	-	-	-	+	-	-	
**Illness beliefs**	Positive	-	+	-	-	-	-	-	+	-	-	
**Feeling old**	Positive	-	-	-	-	-	-	-	-	-	-	
**Feeling abnormal**	Positive	-	-	-	-	-	-	-	+	-	-	
**Emotions/ mood**	Positive	-	+/-	+	+	+	+	+	+	+	-	
**Depression**	Positive	-	+	+	+	+	+	+	+	-	-	
**Fears**	Positive	-	-	-	-	+	+	+	+	-	-	
**Concentration/ intellectual**		-	-	-	-	-	-	-	+	-	-	

**capacity**												
**Treatment beliefs**		-	-	-	-	-		-	+	-	-	
**Uncertainty over the future**	Partial agreement	-	-	-	-	-	-	+/-	+	-	-	
Key:<br>. = silence<br>-/+ = partial agreement<br>- = dissonance<br>+ = agreement												

S5

ePAQ electronic Personal Assessment Questionnaire **Ms Minnie Mouse - 666555 - 111222TEST - 27/11/2019** Page 1 of 7

AAA - Symptom anxiety	—		
AAA - Size increase anxiety	—		
Rupture	—		
Lifting	—		
AAA - Anxiety Independent travel	—		
AAA - ADL Overall impact	—		
AAA - ADL Physical activities	—		
AAA - ADL Personal responsibilities	—		
AAA - ADL Independent living	—		
AAA - ADL Social activity	—		
AAA - ADL Low mood	—		

ePAQ VASC 1.7 Minnie Mouse Completed on 27/11/2019

Name	Minnie Mouse	Date of birth	21/09/1951	NHS Number	111 222 TEST (02)
Clinician	Mr PAD Surgeon	Clinic	Claudication Clinic	Hospital Number	666555

Lower Limb

Lower limb - Vascular disease	Yes	Lower limb - Vascular disease date	Over 5 years ago
Lower limb - Precise diagnosis date			
Lower limb - Treatments	Yes		
Lower limb - Procedures	Bypass graft of a blockage , Balloon stretch of a vessel ,	LL - Other procedures	—
Lower limb - Cramping pain	0 1 **2** 3	Impact	0 **1** 2 3
Lower limb - Walking	Most of the time		
Lower limb - Walking distance	0 1 2 **3**		
Lower limb - Walking speed	0 1 **2** 3		
Lower limb - Walking uphill	0 1 **2** 3		
Lower limb - Climbing stairs	0 1 **2** 3		
Lower limb - Pain at night	Never	Impact	—
Lower limb - Dangling	—		
Lower limb - pain resting	—		
Lower limb - Leg ulcers	—	Impact	—
Lower limb - Ulceration	Yes	Lower limb - Ulceration first notice	3 - 5 years
LL - Ulceration location	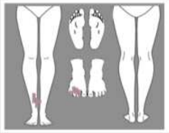		
LL - Ulceration smell	**0** 1 2 3		
LL - Ulceration appearance	**0** 1 2 3		
LL - Ulceration fluid leakage	**0** 1 2 3		
LL - Ulceration infection	**0** 1 2 3		
LL - Ulceration recurrence	No		
LL - Ulceration healing anxiety	**0** 1 2 3		

©ePAQ systems Ltd 2020 Page 1 of 7

ePAQ electronic Personal Assessment Questionnaire **Ms Minnie Mouse - 666555 - 111222TEST - 27/11/2019** Page 2 of 7

ePAQ VASC 1.7 Minnie Mouse Completed on 27/11/2019

Name	Minnie Mouse	Date of birth	21/09/1951	NHS Number	111 222 TEST (02)
Clinician	Mr PAD Surgeon	Clinic	Claudication Clinic	Hospital Number	666555

Carotid Artery Disease

CAD Diagnosis	No	Diagnosis time frame	—
CAD - Diagnosis date			
CAD - Previous TIA	—	CAD - TIA Count	—
CAD - Most recent TIA			
CAD - First TIA			
CAD - Previous Stroke	—	CAD - Stroke Count	—
CAD - Most recent stroke			
CAD - First stroke			
CAD - TIA/Stroke Anxiety	—		
CAD - Anxiety	—		
Health anxiety	—		
Independence anxiety	—		
CAD - Balance	—		
CAD - Memory symptoms	—		
CAD - Altered speech	—		
CAD - Swallowing	—		
CAD - Vision	—		
Right eye symptoms	—		
CAD - Left eye	—		
CAD - ADL overall impact	—		
CAD - Physical activity	—		
CAD - Personal responsibilities	—		
CAD - Independent living	—		
CAD - Social activity	—		

©ePAQ systems Ltd 2020 Page 2 of 7

Frequency of pain	Occasionally	Pain - length of time	Less than 6 months
Pain - ADL Overall impact	0 1 **2** 3		
Sensation change	Yes	Cause	Numb right below knee stump
Sensation change - Location	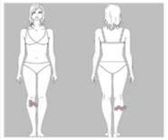		
Sensation change - Description	Stump of right leg has always been numb since my operation		
Sensation change - Frequency	All of the time	Sensation change - Duration	2 or more years
Sensation change - ADL Overall impact	0 **1** 2 3		
Weakness	No	Weakness - Cause	**##QuestionShown##**
Weakness location	—		
Weakness - Description	—		
Weakness - Frequency	—	Weakness - Duration	—
Weakness - ADL Overall impact	—		
BMI	Height:157.48cm Weight:60.33kg BMI: 24		
Smoking status	Ex-smoker (quit more than 1 year ago)	Current habit	
Smoking history	30		
Smoking history cont.	15		
Smoking habit	;		
Diabetes status	Yes	Diabetes duration	3-5 years
Diabetes treatment	Diet , Tablets ,		
Blood clots	None ,		
Medical treatments	Aspirin , Cholesterol lowering (Statin)		

ePAQ VASC 1.7 Minnie Mouse Completed on 27/11/2019

Name	Minnie Mouse	Date of birth	21/09/1951	NHS Number	111 222 TEST

Anxiety		Dimension skipped	
ADL		Dimension skipped	
CNS Symptoms		Dimension skipped	

Aortic aneurysm		Score (0 - 100)	Impact
Quality of Life		Dimension skipped	
Anxiety		Dimension skipped	
AAA Symptoms		Insufficient data	

Lower Limb		Score (0 - 100)	Impact
Pain	0		
Ulceration	0		
Varicose veins		Insufficient data	
ADL	35		
Claudication	62		

ePAQ VASC 1.7 Minnie Mouse Completed on 27/11/2019

Name	Minnie Mouse	Date of birth	21/09/1951	NHS Number	111 222 TEST (02)
Clinician	Mr PAD Surgeon	Clinic	Claudication Clinic	Hospital Number	666555

Generic

Consent	Yes		
Pain	Yes	Cause of pain	Poor circulation in my left leg

Clinician	Mr PAD Surgeon	Clinic	Claudication Clinic	Hospital Number	666555
Smoker	Ex-smoker (quit more than 1 year ago)	Referal reason		Condition change	
Diabetes status	Yes	BMI	24 (Height:157.48 cm) (Weight:60.32778521 kg)		
Goals					
Concerns	1. Poor circulation left leg<br>2. Risk of losing my other leg				
Questions	1. What can be done to treat my poor circulation				

Generic		Score (0 - 100)	Impact
EQ-5D	35		
Pain	67		
Sensation	33		
Weakness		Insufficient data	

Carotid Artery Disease		Score (0 - 100)	Impact
Anxiety		Dimension skipped	
ADL		Dimension skipped	
CNS Symptoms		Dimension skipped	

Aortic aneurysm		Score (0 - 100)	Impact
Quality of Life		Dimension skipped	
Anxiety		Dimension skipped	
AAA Symptoms		Insufficient data	

Lower Limb		Score (0 - 100)	Impact
Pain	0		
Ulceration	0		
Varicose veins		Insufficient data	
ADL	35		
Claudication	62		

ePAQ VASC 1.7 Minnie Mouse Completed on 27/11/2019

Name	Minnie Mouse	Date of birth	21/09/1951	NHS Number	111 222 TEST (02)
Clinician	Mr PAD Surgeon	Clinic	Claudication Clinic	Hospital Number	666555

Generic

Consent	Yes		
Pain	Yes	Cause of pain	Poor circulation in my left leg
Pain - Location	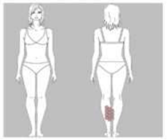		
Pain - Description	Cramping pain on walking		
Frequency of pain	Occasionally	Pain - length of time	Less than 6 months
Pain - ADL Overall impact	0 1 2 3		

©ePAQ systems Ltd 2020

Clinician	Mr PAD Surgeon	Clinic	Claudication Clinic	Hospital Number	666555
Smoker	Ex-smoker (quit more than 1 year ago)	Referal reason		Condition change	
Diabetes status	Yes	BMI	24 (Height:157.48 cm) (Weight:60.32778521 kg)		
Goals					
Concerns	1. Poor circulation left leg<br>2. Risk of losing my other leg				
Questions	1. What can be done to treat my poor circulation				

Generic		Score (0 - 100)	Impact
EQ-5D	35		
Pain	67		
Sensation	33		
Weakness		Insufficient data	

Carotid Artery Disease		Score (0 - 100)	Impact
Anxiety		Dimension skipped	
ADL		Dimension skipped	

©ePAQ systems Ltd 2020

ePAQ electronic Personal Assessment Questionnaire **Ms Minnie Mouse - 666555 - 111222TEST - 27/11/2019** Page 7 of 7

Clinician	Mr PAD Surgeon	Clinic	Claudication Clinic	Hospital Number	666555
Smoker	Ex-smoker (quit more than 1 year ago)	Referal reason		Condition change	
Diabetes status	Yes	BMI	24 (Height:157.48 cm) (Weight:60.32778521 kg)		
Goals					
Concerns	1. Poor circulation left leg<br>2. Risk of losing my other leg				
Questions	1. What can be done to treat my poor circulation				

Generic		Score (0 - 100)	Impact
EQ-5D	35		
Pain	67		
Sensation	33		
Weakness		Insufficient data	

Carotid Artery Disease		Score (0 - 100)	Impact
Anxiety		Dimension skipped	
ADL		Dimension skipped	
CNS Symptoms		Dimension skipped	

Aortic aneurysm		Score (0 - 100)	Impact
Quality of Life		Dimension skipped	
Anxiety		Dimension skipped	
AAA Symptoms		Insufficient data	

Lower Limb		Score (0 - 100)	Impact
Pain	0		
Ulceration	0		
Varicose veins		Insufficient data	
ADL	35		
Claudication	62		

©ePAQ systems Ltd 2020
