# Supplementary material for: Mixed methods study to develop the content validity and the conceptual framework of the electronic patient-reported outcome measure for vascular conditions
Source: BMJ Open. 2020 Aug 11;10(8):e034154. doi: 10.1136/bmjopen-2019-034154 (PMC7418769; doi:10.1136/bmjopen-2019-034154)
Supplement: Author's manuscript [file bmjopen-2019-034154.draft_revisions.pdf]

BMJ Open is committed to open peer review. As part of this commitment we make the peer review history of every article we publish publicly available.

When an article is published we post the peer reviewers' comments and the authors' responses online. We also post the versions of the paper that were used during peer review. These are the versions that the peer review comments apply to.

The versions of the paper that follow are the versions that were submitted during the peer review process. They are not the versions of record or the final published versions. They should not be cited or distributed as the published version of this manuscript.

BMJ Open is an open access journal and the full, final, typeset and author-corrected version of record of the manuscript is available on our site with no access controls, subscription charges or pay-per-view fees (http://bmjopen.bmj.com).

If you have any questions on BMJ Open's open peer review process please email info.bmjopen@bmj.com

The development of the conceptual framework for an electronic patient-reported outcome measure for vascular conditions

Journal:	*BMJ Open*
Manuscript ID	bmjopen-2019-034154
Article Type:	Original research
Date Submitted by the Author:	19-Sep-2019
Complete List of Authors:	Aber, Ahmed; The University of Sheffield, ScHARR; University of Sheffield,<br>Phillips, Patrick<br>Lumley, Elizabeth<br>Radley, Stephen; Sheffield Teaching Hospitals NHS Foundation Trust<br>Thomas, Steve; The University of Sheffield, School of Health and Related Research (ScHARR)<br>Nawaz, Shah; Sheffield Teaching Hospitals NHS Foundation Trust, Sheffield Vascular Institute<br>Jones, Georgina; Leeds Beckett University Faculty of Health and Social Sciences,<br>Michaels, Jonathan; University of Sheffield
Keywords:	Patient reported outcome measures, Vascular surgery < SURGERY, electronic health record (EHR), Quality in health care < HEALTH SERVICES ADMINISTRATION & MANAGEMENT, QUALITATIVE RESEARCH, Mixed methods

SCHOLARONE™
Manuscripts

I, the Submitting Author has the right to grant and does grant on behalf of all authors of the Work (as defined in the below author licence), an exclusive licence and/or a non-exclusive licence for contributions from authors who are: i) UK Crown employees; ii) where BMJ has agreed a CC-BY licence shall apply, and/or iii) in accordance with the terms applicable for US Federal Government officers or employees acting as part of their official duties; on a worldwide, perpetual, irrevocable, royalty-free basis to BMJ Publishing Group Ltd ("BMJ") its licensees and where the relevant Journal is co-owned by BMJ to the co-owners of the Journal, to publish the Work in this journal and any other BMJ products and to exploit all rights, as set out in our licence.

The Submitting Author accepts and understands that any supply made under these terms is made by BMJ to the Submitting Author unless you are acting as an employee on behalf of your employer or a postgraduate student of an affiliated institution which is paying any applicable article publishing charge ("APC") for Open Access articles. Where the Submitting Author wishes to make the Work available on an Open Access basis (and intends to pay the relevant APC), the terms of reuse of such Open Access shall be governed by a Creative Commons licence – details of these licences and which Creative Commons licence will apply to this Work are set out in our licence referred to above.

Other than as permitted in any relevant BMJ Author's Self Archiving Policies, I confirm this Work has not been accepted for publication elsewhere, is not being considered for publication elsewhere and does not duplicate material already published. I confirm all authors consent to publication of this Work and authorise the granting of this licence.

For peer review only

The development of the conceptual framework for an electronic patient-reported outcome measure for vascular conditions.

1) Authors: Ahmed Aber[a], Patrick Phillips[a], Elizabeth Lumley[a], Stephen Radley[b], Steve Thomas[c], Shah Nawaz[c], Georgina Jones[c], Jonathan Michaels[a]
Ahmed Aber, University of Sheffield, a.aber@sheffield.ac.uk
Patrick Phillips, University of Sheffield, p.phillips@sheffield.ac.uk
Elizabeth Lumley, University of Sheffield, e.lumley@sheffield.ac.uk
Stephen Radley, Sheffield Teaching Hospitals, Stephen.Radley@sth.nhs.uk
Steve Thomas, Sheffield Teaching Hospitals, s.m.thomas@sheffield.ac.uk
Shah Nawaz, Sheffield Teaching Hospitals, shah.nawaz@sth.nhs.uk
Georgina Jones, Leeds Beckett University, g.l.jones@leedsbeckett.ac.uk
Jonathan Michaels, University of Sheffield, j.michaels@sheffield.ac.uk

2) [a]Institution: School of Health and Related Research, University of Sheffield, Sheffield
[b]Institution: Jessop Wing, Sheffield Teaching Hospitals, Sheffield
[c]Institution: Sheffield Vascular Institute, Sheffield Teaching Hospitals, Sheffield
[d]Institution: Department of Psychology, School of Social Sciences, Leeds Beckett University, Leeds
3) *Corresponding author: Ahmed Aber, Regent Court, 30 Regent Street, School of Health and Related Research, University of Sheffield, Sheffield, S1 4DA.
E-mail: a.aber@sheffield.ac.uk.
Tel: +44 (0) 114 222 4029
Fax: +44 (0) 114 222 0740

4) Category for manuscript submission: Original Research

5) Source of funding for research and publication: This manuscript presents independent research funded by the National Institute for Health Research (NIHR) under the Programme Grants for Applied Research (RP-PG-1210-12009).
The views expressed are those of the authors and not necessarily those of the NHS, the NIHR or the Department of Health.
6) The authors thank the UK Vascular PROM Group and Cardiovascular Research Patient Panel at Sheffield Teaching Hospitals NHS Foundation Trust.

7) Conflict of interest: Stephen Radley is a shareholder in ePAQ Systems Ltd, an NHS spin-out technology company, the majority shareholder being Sheffield Teaching Hospitals. The other authors have no conflict of interest.

For peer review only

UK Vascular PROM Group:
Ms. Alison Abbott, Vascular Clinical Nurse Specialist.
Mr. Ayman Badawy, Vascular Surgery Associate Specialist.
Mr. Nasim Akhtar, Vascular Consultant Surgeon.
Mr. Faisal, Alam, Vascular Consultant Surgeon.
Mr. Wissam Al-Jundi, Vascular Consultant Surgeon.
Mr. Matthew Armon, Vascular Consultant Surgeon.
Mr. Lukla Biasi, Vascular Consultant Surgeon.
Professor Matt Bown, Vascular Consultant Surgeon.
Professor Bruce Campbell, Vascular Consultant surgeon.
Mr. Tommaso Donati, Vascular Consultant Surgeon.
Mr. Tony Fox, Vascular Consultant Surgeon.
Mr. Andrew Gordon, Vascular Consultant Surgeon.
Professor Linda Hands, Vascular Consultant Surgeon.
Mr. Gary Hicken, Vascular Consultant Surgeon.
Mr. Peter Holt, Vascular Consultant Surgeon.
Dr. Shelley Jackson, General Practioner.
Ms. Brenda King, Specialist Nurse.
Ms. Talia Lea, Vascular Clinical Nurse Specialist.
Mr. Greg McMahon, Vascular Consultant Surgeon.
Mr. John Mosley, Vascular Consultant Surgeon.
Mr. Sanjay Patel, Vascular Consultant Surgeon.
Dr. Gaynor Radley, General Practioner.
Mr. Harjeet Rayt, Vascular Consultant Surgeon.
Professor Rob Sayers, Vascular Consultant Surgeon.
Mr. Paul Tisi, Vascular Consultant Surgeon.

Patient and Public involvement:

The research question and output was developed in consultation with patients and public. The authors thank the Cardiovascular Research Patient Panel at Sheffield Teaching Hospitals NHS Foundation Trust. The aim of the research was to develop a patient focused outcome measure. In this process patients were recruited for two qualitative studies to ensure content validity and face validity of this tool. Patients were involved in every stage of the development of the study. The developed electronic patient assessment questionnaire for vascular conditions (ePAQ-VAS) have been used by patients in a clinical study and there are plans for regular clinical use. The results will also be disseminated in relevant meetings and among patient groups.

Keywords:
Vascular disease, patient-reported outcome measures, quality of life, content validity, qualitative research, electronic questionnaire.

For peer review only

Abstract

Objective:

The aim of this paper is to describe the stages undertaken to generate the items and conceptual framework of a new electronic personal assessment questionnaire for vascular conditions.

Design:

A mixed methods study: a clinician survey followed by quantitative systematic reviews to identify validated patient reported outcome measures (PROM). A qualitative study of patients and systematic reviews of the qualitative evidence were used to develop this new PROM. This was followed by a quantitative clinicians' consensus study and finally, a qualitative face validity study with patients.

Participants:

Vascular patients participated in the primary qualitative study and the face validity study. In the qualitative study fifty-five patients were interviewed and for the face validity nineteen patients gave feedback. Twelve clinicians completed the survey and thirteen completed two cycles of the clinicians' consensus study.

Results:

The items and scales in the electronic personal assessment questionnaire for vascular conditions (ePAQ-VAS) were generated based on the results of five systematic reviews evaluating existing PROM for possible inclusion in ePAQ-VAS, five systematic reviews of qualitative evidence, a primary qualitative study involving 55 patients and clinicians' input. One hundred and sixty-eight items were initially generated; of which 59 were eliminated by the expert panel due to repetition. The instrument was divided into one generic, and three disease-specific sections (abdominal aortic aneurysm (AAA), carotid artery disease (CAD), and lower limb vascular conditions). In each section, items were grouped together into putative scales. Fifty-five items were grouped across eight scales; the remaining items were kept as individual items, because of relevance to service users.

Conclusions:

This multi-dimensional electronic questionnaire covers the most common vascular conditions. This is particularly important for patients presenting with mixed symptoms or multiple conditions. This tool captures symptomatology, HRQoL and other clinically relevant data, such as experience with services and co-morbidities.

For peer review only

Strengths and limitations of the study

- This electronic patient assessment questionnaire for vascular conditions was developed with input from patients and clinicians. We used mixed-methods to ensure content and face validity.
- We examined the validity of existing patient reported outcome measures for vascular conditions and identified a lack of a holistic electronic measure. We systematically reviewed the qualitative evidence and recruited patients to generate qualitative data. The themes generated were used to develop most items.
- Vascular clinicians were surveyed to ensure clinically relevant conditions and questions were included. They were also consulted in a consensus study.
- This holistic assessment tool can be incorporated to the electronic patient records. This can help provide patient focused care, as well as be used to assess outcomes of vascular patients' overtime.
- The burden of questionnaire is its main limitation; however, by providing strict skipping rules only relevant questions are presented to patients. This tool will be examined for other psychometric validity and reliability in future studies.

For peer review only

Introduction

Vascular conditions can cause problems throughout the body; epidemiological studies suggest that both venous and arterial diseases are very common **(1, 2)**. It therefore makes sense to assess individuals with vascular disease holistically; investigating not only existing or potential manifestations of vascular disease, but also the impact of conditions on health-related quality of life. PROM are questionnaires or instruments, designed to elicit information directly from the patient and can be used as part of such an assessment.

Validity and reliability are integral to developing or selecting a PROM. A key aspect of validity is *content validity,* and international guidelines including the U.S. Food and Drug Administration (FDA) guidance stress the importance of this psychometric property **(3)**.

Many generic and condition specific PROM have been adopted to examine impact of vascular conditions on patients and measure outcomes. This is despite a lack of evidence that they have been developed and evaluated in-line with accepted guidelines; in addition, these instruments are rarely used or formally evaluated in routine patient assessment in day to day clinical practice. We conducted scoping searches and informal discussions with vascular clinicians to identify any existing PROM, however, these preliminary stages in the research process suggested an absence of valid and reliable PROM for use in vascular populations.

In this paper, we report the stages in developing an electronic personal assessment questionnaire for vascular patients (ePAQ-VAS). This includes:

(i) Identifying the main vascular conditions to be included in this electronic measure based on a survey of clinicians treating vascular disease.

(ii) Developing a hypothesized framework for the sections for different disease categories based on the previous systematic reviews that identified PROM used in patients with abdominal aortic aneurysm (AAA), carotid artery disease (CAD), peripheral arterial disease (PAD), venous leg ulcers (VLU) and varicose veins (VV) **(4-8).**

(iii) Developing the items within each section of ePAQ-VAS based on qualitative systematic reviews **(9-12),** and a primary qualitative study **(13).**

(iv) A consensus study with clinicians to rate the relevance of included items, and to add items to ePAQ-VAS based on the opinion of vascular surgeons, radiologists and nurses.

(v) A face validity study with vascular patients to examine the clarity and relevance of the items within ePAQ-VAS.

The aim of these steps was to develop a single electronic instrument covering most vascular conditions in line with international guidance **(3)**. The conceptual framework and items were developed in a way to ensure this assessment tool can be used in patients with mixed symptoms and multiple vascular conditions.

Methods

Clinicians involved in the care of vascular patients were invited to identify the common vascular conditions treated by vascular surgeons and vascular specialists. They were asked to list the key issues, symptoms and the impact of these conditions on patients suffering with these diseases. Data from this round were used to inform qualitative evidence synthesis.

The conceptual framework of ePAQ-VAS was based on primary qualitative interviews with vascular patients, input from clinicians, systematic reviews examining the validity of existing PROM, and qualitative reviews of the impact of vascular diseases on quality of life. Ethical approval was obtained from Yorkshire & Humber NRES committee– Bradford Leeds (REC Number: 14/YH/1117) on 25.09.2014. Figure 1. Illustrates the process used to develop ePAQ-VAS in accordance to FDA guidelines **(3)**.

For peer review only

Figure 1. Development of ePAQ-VAS conceptual framework

Insert figure 1 here

Systematic Reviews to Identify and Appraise Existing PROM

Systematic searches were conducted of bibliographic databases including CINAHL via EBSCO, MEDLINE and MEDLINE in Process via Ovid, EMBASE via Ovid, PsycINFO via Ovid, Social Science Citation Index/Science Citation Index via Web of Science (Thomson Reuters), and Proquest dissertations and theses. PROM were included where there was evidence that they had undergone some form of psychometric evaluation that would allow the validity, reliability and responsiveness of the PROM to be assessed. Included PROM were categorised per type, (generic or condition specific) and the vascular population(s) in which they had been validated. Quality assessment **(3, 14)** was conducted to identify high quality existing PROM for possible direct inclusion in ePAQ-VAS, or to be used as a basis to inform the qualitative evidence synthesis.

Primary qualitative study

Semi-structured interviews were conducted with 55 vascular patients from Sheffield Teaching Hospitals NHS Foundation Trust (STH) following purposeful sampling to ensure a range of participants of different age and gender, at different stages of treatment and covering the main five vascular conditions (AAA, PAD, CAD, VLU, VV). Questions were asked about the signs, symptoms, and impact of the condition on function and lifestyle. These conditions were identified by the vascular surgeons and other clinicians' involved in the care of vascular patients.

Systematic reviews of the qualitative evidence

Systematic searches of the following databases; CINAHL via EBSCO, MEDLINE and MEDLINE in Process via Ovid, EMBASE via Ovid, PsycINFO via Ovid, Social Science Citation Index/Science Citation Index via Web of Science (Thomson

For peer review only

Reuters), and Proquest dissertations were conducted to identify existing qualitative research detailing vascular patients' experience of living with AAA, PAD, CAD, VLU, and VV.

Analysis of the qualitative evidence

Qualitative data from the primary study and each of the systematic qualitative reviews were analysed separately. Framework analysis was used to analyse the interviews **(15).** This analysis includes five stages,

- the first involved familiarization by reading of the transcripts and reading the primary data.
- The second stage involved identification of a thematic framework; the thematic framework was based either on clinical opinion for areas with no valid PROM, such as AAA and CAD, or a combination of clinical opinion and, when available, the scales of PROM with good content validity.
- In the third stage the data was coded and indexed by applying the thematic framework to the whole data set.
- At the fourth stage, a framework matrix was created by arranging the data per the thematic references.
- finally mapping and interpretation, including examining patterns within the data and associations with it.

Clinicians input and consensus exercise

Twenty-three clinicians involved in the care and management of patients with vascular conditions were invited to a survey to list the most common vascular conditions managed by them and to list the key issues, symptoms and the impact of these diseases on patients. Data from this round were used to inform qualitative evidence synthesis.

Different group of clinicians involved in the care of vascular patients were invited to a consensus study to score the relevance of items (questions) in the provisional version of ePAQ-VAS. In total, thirty clinicians including vascular surgeons, interventional radiologists, vascular nurses, physiotherapists and occupational therapists were invited. Participants were asked to rate the appropriateness of each question on a five-

For peer review only

point Likert scale of "Strongly disagree" (=0) to 'Strongly agree' (=4). This process was repeated, and members of the clinicians' panel were presented with the aggregate findings of the previous round, and again asked to score each question. This process aimed to examine the relevance of each item from the clinicians' perspective and to identify any new items suggested by the clinicians **(16)**.

Developing scales and items

The ePAQ development team (AA, EL, PP, GJ, SR) employed an iterative process, incorporating evidence from the systematic reviews, qualitative study, and the clinicians' consensus study. In line with the FDA guidance **(3)**, items (questions) were developed from the qualitative data using the following three steps: interpretation, translation, and triangulation of themes.

Interpretation involved familiarisation with the language used in the primary data included in the synthesis. This enabled translation of descriptions of apparently diverse issues affecting vascular patients into a single set of harmonised themes. The resulting themes were used to develop the items for ePAQ-VAS. The items were grouped into sections, and each section, further divided into scales consisting of a connected group of items. Triangulation was performed across evidence sources to ensure the items comprehensively covered all issues of importance to patients with AAA, PAD, CAD, VLU, and VV.

Face validity of ePAQ-VAS

A second phase of semi-structured patient interviews was conducted by (EL, PP) with 19 participants, purposeful sampled from the vascular populations previously described. ePAQ-VAS (version 1) was presented to participants and a focused interview was conducted to investigate vascular patients' perceptions of the questionnaire as a whole, and individual items. Questions were asked under the following headings of:

- overall impressions,

For peer review only

- clarity,
- relevance and emotional response.

Interviews were audio taped, transcribed and analysed. A pragmatic approach was used for the analysis, with comments collated and presented back to the working group who made consensus decisions on revisions to ePAQ-VAS.

Results

In total twelve clinicians completed the first survey and identified PAD, AAA, VLU, VV and CAD as the most common vascular conditions treated by them. They listed common issues such as pain on walking, rest pain, reduced mobility or lack of mobility for patients with PAD, no physical symptoms for those with AAA but need for multidisciplinary approach to manage these patients. For patients with VLU, the main issues included burning pain, recurrence and healing; for patients suffering with VV, skin changes, appearance of leg and ulcer as well as ache were the main issues raised. The clinicians felt the key issue for patients with CAD was identifying patients benefiting from intervention and reducing the risk of stroke. The result from this survey was used to inform the analysis of qualitative data used to develop ePAQ-VAS.

Systematic reviews and assessment of psychometric evaluation were conducted for PROM validated for use in PAD, AAA, VLU, VV and CAD. A total of 33 PROM that had undergone some form of validation were identified in 41 studies **(Table 1.)**.

Condition	Number of Citations	Number of Included Papers	Results	Conclusions
AAA	1232	3	4 validated PROMs identified –1 generic, 1 vascular generic and 2 condition-specific	This review has highlighted a gap in the evidence for validated PROMs in AAA. Due to a lack of rigorous psychometric testing.
CAD	1670	5	6 validated PROMs identified –	There was a lack of validated PROMs to

For peer review only

			4 generic and 2 condition-specific	measure outcomes for CAS patients.
PAD	6981	14	13 validated PROMs identified – 6 generic and 7 condition-specific	VascQol was the most psychometrically robust instrument.
VV	3879	7	3 validated PROMs identified – 1 generic and 2 condition-specific	Aberdeen Varicose Vein Questionnaire is the most psychometrically robust disease specific PROM for use with VV patients.
VLU	3879	6	7 validated PROMs identified – 3 generic and 4 condition-specific	The most valid and reliable condition specific PROM was VLU-QOL.
Abbreviations: Abdominal aortic aneurysm (AAA), Carotid artery disease (CAD), Peripheral arterial disease (PAD), Venous leg ulcer (VLU), Varicose veins (VV).				

Table 1: Results from the systematic reviews of psychometric evaluation of vascular PROMs

No PROM were identified that had undergone sufficiently rigorous development and validation to suggest that they were suitable for direct use in ePAQ-VAS, the details of these reviews have been reported previously **(4-8).** Where evidence existed, this fell short of required standards **(3,14)**. For instance, the review investigating VV PROM **(4)** found some evidence for, and discussion of, content validity in relation to the Aberdeen varicose vein questionnaire (AVVQ) and suggest that it is the most appropriate existing condition specific measure for use in a VV population. However, item generation for this PROM involved a literature review and assessment by clinicians of relevance of included items with no direct involvement of patients, therefore suggesting a deficiency in terms of content validity **(17)**. The scales from these reviews were used to provide a framework for the systematic qualitative reviews and the primary qualitative study **(5, 9-13)**.

For peer review only

In total 111 patients were approached but only fifty-five patients (69.1% male) were interviewed about their experience of living with vascular disease, ages ranged from 35-77 years. Summary results are shown in **Table 2**.

Condition	Sample size	Key Findings
AAA	13	No physical symptoms, a small number of participants reported abdominal pain and pain in their legs. Uncertainty, anxiety and fear of rupture and death appeared to impact most greatly on people's QoL.
CAD	9	This condition seemed to have had the least impact on physical and social function, although psychologically it created a sense of worry and anxiety for some participants. The main reported outcome was fear of having a major stroke.
PAD	14	Pain and mobility were the most commonly reported themes. The extent to which they impacted on QoL was associated with the severity, age expectations and social support. Fear of the symptoms worsening and amputation was evident.
VV	10	VV do not appear to have had a major impact on overall QoL for most the participants. Pain was the most common issue. The perceived unpleasant appearance of the VV seemed to have the greatest psychological impact. Many of the participants had had their VV for very long periods of time, often just "putting up with it'' for numerous years before seeking help.
VLU	10	The impact of VLU on QoL differed within the group. For some there were no major issues and having a VLU was accepted as part of their current life, with the hope that it would heal eventually. For others, there was a far more significant effect. Pain was quite severe for some participants leading to a significantly reduced QoL. VLU appeared to have a significant psychological impact causing a high degree of distress for some.
Abbreviations: Abdominal aortic aneurysm (AAA), Carotid artery disease (CAD), Peripheral arterial disease (PAD), Venous leg ulcer (VLU), Varicose veins (VV).		

Table 2: Findings from the primary qualitative study with vascular patients

Six overarching themes relating to the impact of the five vascular conditions were identified. These were symptoms (including pain), impact on physical function, social

For peer review only - http://bmjopen.bmj.com/site/about/guidelines.xhtml

impact, psychological impact, financial impact and lifestyle. Identified signs, symptoms and impact of the conditions were then mapped and tabulated to see which themes were relevant to which condition, and where the similarities and differences lay **(Table 3.)**.

	**PAD**	**AAA**	**CAD**	**VV**	**VLU**
**Symptoms**					
No symptoms		x	x		
Pain	x	x	x	x	x
Neck pain			x		
Leg pain	x	x	x	x	x
Abdominal pain	x	x			
Arm pain	x				
Cramp/ aching	x	x		x	x
Burning sensation					x
Pain severity	x	x	x	x	x
Pain on walking	x	x		x	x
Pain at rest	x			x	
Pain when standing				x	x
Mobility	x	x	x	x	x
Distance	x	x		x	x
Speed	x	x			
Stairs/ slopes	x	x			
Non-healing wounds	x				x
Co-morbidities	x	x	x		x
Progression of symptoms	x	x		x	x
Sleep	x		x	x	x
Swelling			x	x	
Loss of balance			x		
Confusion			x		
**Impact on physical functioning**					
Hobbies	x			x	x
Exercise	x	x		x	x

For peer review only

Daily activities	x				x
**Social impact**					
Travel	x	x	x		
Social activities	x	x		x	x
Social support	x	x	x		
**Psychological impact**					
Anxiety	x	x	x	x	x
Depression	x	x			x
Feelings of loss	x			x	x
Health expectations	x	x	x	x	x
Unsightly appearance				x	
Feeling self-conscious	x			x	x
Fear of worsening symptoms	x	x	x	x	x
Fear of rupture death		x			
Fear of amputation				x	x
Fear of stroke			x		
**Financial impact**					
Income	x	x			x
Time off work				x	x
**Lifestyle**					
Smoking	x	x	x	x	x
Exercise	x	x	x	x	
Diet	x	x	x		x
Weight				x	x
Abbreviations: Abdominal aortic aneurysm (AAA), Carotid artery disease (CAD), Peripheral arterial disease (PAD), Venous leg ulcer (VLU), Varicose veins (VV).					

Table 3: Map of symptoms and Quality of life concepts across five conditions.

For peer review only

A total of 31 studies were included across the five reviews of existing qualitative research **(6, 10-13)**. A short summary of the main themes to emerge for each condition is shown in **Table 4**.

Condition	Numbers of Citations	Number of Included Studies	Key Themes
AAA	315	3	Anxiety and *lack* of physical symptoms.
CAD	964	3	Symptoms, psychological and social impact, risk and service experience.
PAD	973	9	Pain, compromised physical function, impact on social life.
VV	1804	3	Adaptation – coping strategies employed to limit various impacts, appearance of VV.
VLU	1804	13	Pain, odour and exudate - impact on sleep, mobility and mood.
Abbreviations: Abdominal aortic aneurysm (AAA), Carotid artery disease (CAD), Peripheral arterial disease (PAD), Venous leg ulcer (VLU), Varicose veins (VV).			

Table 4: Results from Qualitative Reviews examining the impact of the major vascular conditions on quality of life

For peer review only

The themes from the first round of the clinicians' consensus study, as well as scales of identified PROM, were used to inform the framework analysis of the qualitative data. Items from existing PROM were then mapped against emerging themes from the qualitative study, and the qualitative review synthesis for each condition, to explore which PROM items or scales captured themes deemed to be the most pertinent to patients. A triangulation approach was followed, whereby researchers evaluated whether the concepts were the same (agreement), offered similar concepts (partial agreement), were in contradiction (dissonance) or were not present (silence). An example of this triangulation approach is provided in **Appendix 1**.

The ePAQ-VAS development team used the findings from the triangulation for AAA, PAD, VV, VLU and CAD to develop themes for distinct sections relevant for each of

these vascular conditions. The primary qualitative data were used to create each item. Items were then grouped into sections and within each section there were scales consisting of items that measured the same latent variable such as anxiety related to the diagnosis of AAA. The results of the clinicians' consensus study were considered to add further items to the relevant sections **(Table 5)**.

The items of ePAQ-VAS were arranged into four sections; generic, AAA, CAD and lower limb (LL) vascular conditions. A single LL section was developed as common themes were identified for conditions affecting the legs, regardless of whether the underlying pathology was venous or arterial. An inclusive approach to development was used and a comprehensive questionnaire was produced with 168 questions. (See Figure 2 for an overview of the process to develop ePAQ-VAS).

Section	Scale	Clinicians' consensus Study	Most valid Condition Specific PROMs			Qualitative study					Qualitative Reviews					Question Text
			VLU-QOL	PADQOL	AVVQ	AAA	CAD	VV	VLU	PAD	AAA	CAD	VV	VLU	PAD	
Generic	Pain	4	X	X	X	X	X	X	X	X	X	X	X	X	X	Do you suffer with any pain?
Generic	Pain	4	X	-	-	X	X	X	X	X	X	X	X	X	X	Use the below Image and click on body parts where you experience pain or discomfort.
Generic	Pain	-	X	-	-	X	X	X	X	X	X	X	X	X	X	Please use your own words to describe this problem.
Generic	Pain	4	X	X	X	X	X	X	X	X	X	X	X	X	X	How often do you experience a significant amount of pain?
Generic	Pain	4	-	-	-	X	X	X	X	X	X	X	X	X	X	How much do problems caused by pain affect your overall enjoyment of life?
Generic	Sensation	4	-	-	-		X	X		X	X	X	X		X	Do you experience any numbness or pins and needles in any part of your body?
Gen	Sen	4	-	-	-		X	X		X	X	X	X		X	Please use the image below to select where you

eric		sation															experience sensation change in your body.
Generic		Sensation	-	-	-	-		X	X		X	X	X	X		X	Please use your own words to describe this problem.
Generic		Sensation	4	-	-	-		X	X		X	X	X	X		X	How often do you experience numbness or pins and needles?
Generic		Sensation	4	-	-	-		X	X		X	X	X	X		X	How much do problems caused by numbness or pins & needles affect your overall enjoyment of life?
Generic		Weakness	4	-	-	-						X	X			X	Do you have any loss of strength or weakness in any part of your body?
Generic		Weakness	3	-	-	-						X	X			X	Please use the image below to indicate the areas where you experience any physical weakness.
Generic		Weakness	-	-	-	-						X	X			X	Please use your own words to describe this problem.
Generic		Weakness	4	-	-	-						X	X			X	How often do you experience loss of strength or weakness?
Generic		Weakness	3	-	-	-						X	X			X	How much do problems caused by weakness affect your overall enjoyment of life?
Carotid		Anxiety	4					X					X				Do you worry about having a stroke?
Carotid		Anxiety	3					X					X				Does carotid artery disease make you feel anxious?
Carotid		Anxiety	New item														Are you worried about your health getting worse because of carotid artery disease?
Carotid		Anxiety	New item														Are you worried about losing your independence because of carotid artery disease?
Carotid		Symptoms	2					X									Do you have any problems with maintaining your balance?
Carotid		Symptoms	2					X									Do you suffer with any problems with your memory? (E.g. Forgetting or losing things)
Carotid		Symptoms	4					X					X				Have you had any problems with your speech? (E.g. Slurring your words or not being able to speak or say things properly)
Carotid		Symptoms	New item														Do you have any problems with swallowing food?
Carotid		Symptoms	4					X					X				Have you had any problems with partial or complete loss of vision in either of your eyes?
Carotid		Symptoms	4					X					X				How would you describe any loss of vision in your RIGHT eye?
Carotid		Symptoms	4					X					X				How would you describe any loss of vision in your LEFT eye?
Carotid		ADL	3					X					X				How much do problems caused by carotid artery disease (anxiety associated with diagnosis or physical symptoms) affect your overall enjoyment of life?
Carotid		ADL	4					X					X				How much do problems caused by carotid artery disease (e.g. mini-stroke or stroke memory balance speech visual or other related issues) affect your physical activities such as exercise walking or running?

Carotid		ADL	3					X					X				How much do problems caused by carotid artery disease (e.g. mini-stroke or stroke memory balance speech visual or other related issues) affect your ability to undertake personal roles and responsibilities such as caring for others study or work?
Carotid		ADL	4					X					X				How much do problems caused by carotid artery disease (e.g. mini-stroke or stroke memory balance speech visual or other related issues) affect your ability to look after yourself?
Carotid		ADL	3					X					X				How much do problems caused by carotid artery disease (e.g. mini-stroke or stroke memory balance speech visual or other related issues) affect your social activities such as visiting friends and family?
Carotid		ADL	2					X					X				How much do problems caused by carotid artery disease visual or other related issues) affect your mood?
AAA		Symptoms	4				X					X					Do you have any abdominal (tummy) pain?
AAA		Symptoms	New item (3)														Do you experience a throbbing feeling in your abdomen (tummy)?
AAA		Anxiety	4				X					X					Do you worry about aortic aneurysm?
AAA		Anxiety	4				X					X					Do you worry about any symptoms you experience that may be caused by aortic aneurysm?
AAA		Anxiety	4				X					X					Do you worry about possible increase in the size of your aneurysm?
AAA		Anxiety	4				X					X					Do you fear sudden death or rupture of your aortic aneurysm?
AAA		Anxiety	4				X					X					Do you avoid physical exertion because of having an aortic aneurysm?
AAA		Anxiety	3				X					X					Do you avoid travelling independently because of aortic aneurysm?
AAA		ADL	3									X					How much do problems caused by aortic aneurysm affect your overall enjoyment of life?
AAA		ADL	4				X					X					How much does aortic aneurysm affect your physical activities? (E.g. Exercise walking or going out)
AAA		ADL	3				X					X					How much does aortic aneurysm affect your ability to undertake personal roles and responsibilities? (E.g. Caring for others study or work?
AAA		ADL	3									X					How much do you feel aortic aneurysm affects your ability to look after yourself? (E.g. Rest wash toilet or feed yourself)
AAA		ADL	4									X					How much does aortic aneurysm affect your social activities? (E.g. Visiting friends or family)
AAA		ADL	4				X					X					Do you suffer from low mood because of having an aortic aneurysm?
LL		Ischaemic Pain	4	+/-	+/-				X	X	X			X		X	Do you experience any cramping pain in your legs or feet?
LL		Ischaemic Pain	4		+/-				X		X					X	Do you experience cramping pain in your legs or feet when walking?
LL		Ischaemic Pain	4		+/-						X					X	How far can you walk before you experience any cramping pain in your legs or feet?
LL		Ischaemic Pai	4		+/-						X					X	Do you walk more slowly than you would choose to in order to avoid cramping pain in your legs and feet?

		n															
LL		Ischaemic Pain	4		-						X					X	Do you experience cramping pain in your legs or feet when walking uphill?
LL		Ischaemic Pain	4		-						X					X	Do you experience pain in your legs or feet when you climb stairs?
LL		Ischaemic Pain	4		-				X	X	X			X	X	X	Do you experience pain in your feet at night?
LL		Ischaemic Pain	4		-												Do you dangle one or both of your legs over the side of the bed to help reduce foot pain?
LL		Ischaemic Pain	4		-				X	X	X			X	X	X	Do you experience severe pain in your legs or feet when you are resting or sitting?
LL		Ischaemic Pain	2		-											X	Are you troubled by cold feet?
LL		Ulcer	4	X	-					X	X			X	X	X	Have you ever had any ulcers on your legs or feet now or at any time in the past?
LL		Ulcer	4		-					X	X			X	X	X	Please use the image below to show where you currently have any leg or foot ulcers.
LL		Ulcer	4	-	-					X					X	X	Are you concerned about the smell of your leg ulcers?
LL		Ulcer	4	X	-										X	X	Are you concerned about the appearance of your leg ulcers?
LL		Ulcer	4	-	-										X	X	Do you have leg ulcers that leak fluid (watery liquid)?
LL		Ulcer	4	-	-										X	X	Do you experience infections in your leg ulcers? (E.g. foul smell or pus)
LL		Ulcer	4	-	-					X					X	X	Do you experience repeated leg ulcers?
LL		Ulcer	4	X	-					X					X	X	Do you worry about your leg ulcers? (E.g. Not healing becoming infected losing part of your leg or foot).
LL		VV	4		-	-								X			Do you experience any bleeding from veins in your legs or feet?
LL		VV	4		-	X			X					X			Do you have any problems with the skin over your varicose veins?
LL		VV	4		-	X			X					X			Do varicose veins make you feel self-conscious or embarrassed?
LL		VV	4	X	-	X			X					X	X	X	Do leg or foot problems affect what clothing or shoes you can wear?
LL		VV	4		-	X			X					X	X		Do you experience any swelling in your legs or feet?
LL		VV	4		-	X			X					X	X	X	Do you experience itching in your legs or feet?
LL		VV	4		-	X			X					X	X		Do you wear compression stockings or tights for your legs?
LL		Tissue loss	4		-	-				X	X					X	Have you lost any part of your legs or feet through amputation or gangrene?
LL		Tissue loss	4			-				X	X					X	Please click on the appropriate part or parts of your legs feet or toes that you have had amputated or have been lost â€¦
LL		Anxiety	3	X	X	X			X	X	X			X	X	X	Do you worry about your leg problems getting worse in the future?
LL		ADL	3	+/-	X	+/-			X	X	X			X	X	X	How much do leg or foot problems affect your overall enjoyment of life?

LL		ADL	3	+/-	X	-			X	X	X			X	X	X	How much do leg or foot problems affect your ability to carry out physical activities? (E.g. Walking housework or exercise)
LL		ADL	3	+/-	X	+/-			X	X	X			X	X	X	How much do leg or foot problems affect your personal responsibilities? E.g. caring for others study or work
LL		ADL	3	-	X	+/-			X	X	X			X	X	X	How much do leg or foot problems affect your ability to look after yourself? (E.g. Rest wash toilet or feed yourself)
LL		ADL	3	+/-	X	+/-			X	X	X			X	X	X	How much do leg or foot problems affect your social activities? (E.g. Going out visiting friends or family)
LL		ADL	4	X	X	-			X	X	X			X	X	X	Do you suffer from low mood because of leg or foot problems?
Generic		Personal data		-	X	-	X	X		X	X	X	X		X	X	Do you rely on any people to help you with your everyday activities?
Generic		Personal data		-			X		X	X	X	X			X	X	Do you experience financial problems because of your vascular condition?
Abbreviations: Abdominal aortic aneurysm (AAA), Aberdeen varicose veins questionnaire (AVVQ), Activity of daily living (ADL), Carotid artery disease (CAD), Peripheral arterial disease (PAD), , Lower limb (LL), Peripheral arterial disease (PADQOL), Venous leg ulcer (VLU), Venous leg ulcer Quality of life (VLU-QOL), Varicose veins (VV). The items of the eight scales are colored in this table.																	

Table 5: Structure of the main ePAQ-VAS with evidence base for inclusion of individual items, scales and sections.

Figure 2. Evidence synthesis to develop of ePAQ-VAS

Insert figure 2 here

ePAQ-VAS was presented to 19 vascular patients. Overall the response was positive; with participants reporting that they felt it was fit for purpose and potentially useful. There was little consistency in items that participants found difficult and no individual item was identified with which most participants had difficulty.

Discussion included the use of abbreviations, font size and contrast between text and background, response options and scales, electronic format versus paper format, relevance to patients and clinicians, the use of free text boxes and the language and wording used, when and how to use the skip button, repetition of items and subject matter and the possibility of emotional distress associated with questions about the possibility of deterioration or death.

Figure 3. Overview of ePAQ-VAS structure

For peer review only

Insert figure 3 here

Based on the findings from the face validity exercise, and input from the Vascular PROM group, further revisions were made in an iterative process; culminating in the development of ePAQ-VAS. The structure of the questionnaire is illustrated in Figure 3. Fifty-nine items were eliminated for overlap; these include questions asking about common symptoms experienced across most vascular conditions. Five items were added based on suggestion from clinicians. Generic items for all respondents were presented in the first section and include questions about pain, altered sensation, weakness, weight/height, smoking habit, previous medical history and regular medication. This information was deemed important for assessment of vascular conditions both by patients and clinicians.

The next three sections are condition specific relating to CAD, AAA and LL vascular disease sections. These sections are further divided into scales. There are fifty-five items within eight scales and the remainder of questions do not contribute to scales but are kept due to their clinical relevance. The eight scales are part of the of condition specific sections and include CAD related anxiety, impact of CAD on activities of daily living (ADL), AAA related anxiety, Impact of AAA on activities of ADL, PAD symptoms, VLU symptoms, VV symptoms and Impact of LL vascular disease on ADL. Individual items, scales and sections of ePAQ-VAS in its initial version can be viewed on https://demo-questionnaire.epaq.co.uk/home/project?id=VASC_1.6&page=1

The evidence used to develop each item in ePAQ-VAS is made explicit in **Table 5**, this table show whether the source for the item is the qualitative study, reviews or consensus study.

Discussion

This study documents stages undertaken to develop ePAQ-VAS and the conceptual framework underpinning this new tool for use in undifferentiated vascular populations. The main strength of this new instrument is that it can be used as a holistic clinical assessment tool that can be completed by patients before meeting the

For peer review only

vascular surgeon in the clinic. The information generated can be used to help shared decision making by focusing on patient priorities. This tool has the further advantage of being an electronic online patient reported outcome measure since it can be used to monitor impact of the disease and/or interventions overtime. Furthermore, this instrument is preference based, unlike the identified vascular PROM **(4-8)**; once further validated the disease specific scales can be used to generate utility values either by mapping to the values of a generic utility measure, or by further utility studies **(18).**

This instrument has been developed in line with FDA guidelines for developing PROM **(3)**. Items were developed based on themes extracted from primary qualitative data, systematic qualitative evidence synthesis and clinicians' consensus exercise **(9-13)**. We have made efforts through purposive sampling to ensure that we have included diverse demographic groupings in the primary research, and this is augmented by the inclusion of systematic reviews that include evidence gathered in national and international studies. Another strength of this study is that the qualitative evidence in the review and the primary study included patients at different stages of their disease. The data collected included the impact of disease, including symptoms, on daily living and the impact of diagnosis and treatment on the daily living. The vascular clinicians' input into developing and rating the items was sought, and new items were incorporated based on recommendation from twenty-five vascular clinicians.

The work of developing individual items and their assignment to putative scales and sections was based both on the framework of existing PROM **(4-8),** and on input from vascular clinicians. In this stage of the ePAQ-VAS development, an inclusive approach was chosen, and all relevant items were incorporated except for those with clear repetition. This has resulted in a draft version that is long and potentially repetitive, but comprehensive; It is expected that factor analysis and psychometric testing will lead to a reduction in the number of individual items and will confirm (or refute) the putative scales identified in the current version.

As stated by the FDA **(3)** a fundamental consideration in the development of PROM is the adequacy of item generation. Due to the heterogeneous nature of vascular

For peer review only

disease, it was not straightforward to identify what exactly should be measured when developing and defining the initial conceptual framework for the ePAQ-VAS. To this end, as recommended by the FDA, the initial conceptual framework was based on information gathered from reviews of the literature, patients and expert opinion.

The findings of the qualitative study indicated an overlap in patient experiences of the various conditions. However, there was also a clear difference in how each condition impacted on different aspects of quality of life. This demonstrated that while it may be possible to develop a PROM for use across a variety of vascular conditions, it would also need to include condition-specific items to fully capture the impact and clinically relevant information for each disease or condition.

In conclusion ePAQ-VAS is a multi-dimensional measure developed for use in a range of vascular conditions. It is a single electronic tool, covering most vascular conditions. This is important for those patients presenting with mixed symptoms or multiple conditions. The items in ePAQ-VAS can capture information about disease symptoms, HRQoL, co-morbidities, medical history and other relevant healthcare issues. This type of information can aid communication between healthcare professionals and patients and support shared-decision making. The electronic format may make it easier to monitor patients over time, especially those with chronic conditions and those treated with lifestyle modification or conservatively. Based on methods used in its construction, this tool has a strong degree of content validity; however, further psychometric testing for reliability, responsiveness, and validity is needed. Once this electronic PROM is validated, it can be used as an outcome measure in clinical practice and research.

Contributorship statement

JAM, GJ, AA, SCR designed the study. AA, PP, EL, SCR, GJ performed the analysis of the data and theme generation. ST and SN helped with consensus study and qualitative study recruitment. AA wrote the paper and all authors reviewed and edited the paper. All authors approved the manuscript.

For peer review only

Competing interests

Stephen Radley is a shareholder in ePAQ Systems Ltd, an NHS spin-out technology company, the majority shareholder being Sheffield Teaching Hospitals. The other authors have no conflict of interest.

Funding

This manuscript presents independent research funded by the National Institute for Health Research (NIHR) under the Programme Grants for Applied Research (RP-PG-1210-12009).

Data sharing statement

No data are available.

For peer review only

References:

1- Beebe-Dimmer JL, Pfeifer JR, Engle JS, Schottenfeld D. The epidemiology of chronic venous insufficiency and varicose veins. Ann Epidemiol. 2005 Mar;15(3):175-84.

2- Peach G, Griffin M, Jones KG, Thompson MM, Hinchliffe RJ. Diagnosis and management of peripheral arterial disease. BMJ. 2012 Aug 14;345:e5208.

3- Food and Drug Administration (FDA), Guidance for Industry: Patient-Reported Outcome Measures: Use in Medical Product Development to Support Labeling Claims, accessed and available at: https://www.fda.gov/downloads/drugs/guidances/ucm193282.pdf

4- Aber A, Poku E, Phillips P, Essat M, Buckley Woods H, Palfreyman S, Kaltenthaler E, Jones G, Michaels J. Systematic review of patient-reported outcome measures in patients with varicose veins. Br J Surg. 2017 Oct;104(11):1424-1432.

5- Duncan R, Essat M, Jones G, Booth A, Buckley Woods H, Poku E, Kaltenthaler E, Keetharuth AD, Palfreyman S, Michaels J. Systematic review and qualitative evidence synthesis of patient-reported outcome measures for abdominal aortic aneurysm. Br J Surg. 2017 Mar;104(4):317-327. doi: 10.1002/bjs.

6- Poku E, Duncan R, Keetharuth A, Essat M, Phillips P, Woods HB, Palfreyman S, Jones G, Kaltenthaler E, Michaels J. Patient-reported outcome measures in patients with peripheral

arterial disease: a systematic review of psychometric properties. Health Qual Life Outcomes. 2016 Nov 24;14(1):161.

7- Poku E, Aber A, Phillips P, Essat M, Buckley Woods H, Palfreyman S, Kaltenthaler E, Jones G, Michaels J. Systematic review assessing the measurement properties of patient-reported outcomes for venous leg ulcers. 2017 Oct;1(5):138-147.

8- Essat M, Aber A, Phillips P, Poku E, Woods HB, Howard A, Palfreyman S, Kaltenthaler E, Jones G, Michaels J. Patient-Reported Outcome Measures in Carotid Artery Revascularization: Systematic Review and Psychometric Analysis. Ann Vasc Surg. 2018 Jul;50:275-283.

9- Aber A, Lumley E, Phillips P, Woods HB, Jones G, Michaels J. Themes that Determine Quality of Life in Patients with Peripheral Arterial Disease: A Systematic Review. Patient. 2018 May 8. doi: 10.1007/s40271-018-0307-7.

10- Phillips P, Lumley E, Duncan R, Aber A, Woods HB, Jones GL, Michaels J. A systematic review of qualitative research into people's experiences of living with venous leg ulcers. J Adv Nurs. 2018 Mar;74(3):550-563.

11- Aber A, Howard A, Woods HB, Jones G, Michaels J. Impact of Carotid Artery Stenosis on Quality of Life: A Systematic Review. Patient. 2018 Oct 17. doi: 10.1007/s40271-018-0337-1.

12- Lumley E, Phillips P, Aber A, Woods HB, Jones G, Michaels J. Experiences of living with varicose veins: a systematic review of qualitative research. J Clin Nurs. 2018 Nov. J Clin Nurs. 2018 Nov 21. doi: 10.1111/jocn.14720.

13- Duncan R, Lumley E, Tod A, Hughes J, Palfreyman S, Nawaz S, Jones G, Michaels J. The experiences of people living with a vascular condition: a qualitative study. HEDS Discussion Papers, University of Sheffield. 2018 Jul. https://www.sheffield.ac.uk/scharr/sections/heds/discussion-papers/18_07-1.794000 (Accessed 19 November 2018).

14- Mokkink LB, Terwee CB, Patrick DL, Alonso J, Stratford PW, Knol DL et al. The COSMIN study reached international consensus on taxonomy, terminology, and definitions of measurement properties for health-related patient-reported outcomes. J Clin Epidemiol 2010; 63: 737–745.

15- Ritchie J, Spencer L. Qualitative data analysis for applied policy research. In: Bryman G, Burgess A, editors. Analysing qualitative data. London: Routledge; 1994. p. 173–94.

16- Hsu, C., & Sandford, B. (2007). The Delphi Technique : Making Sense Of Consensus. Practical Assessment, Research & Evaluation, 12(10). http://www.endoedu.com/mobile/guideline/delphi.pdf (Accessed 19 November 2018).

17- Garratt AM, Macdonald LM, Ruta DA, Russell IT, Buckingham JK, Krukowski ZH. Towards measurement of outcome for patients with varicose veins. Qual Health Care 1993; 2: 5–10

18- Brazier J, Deverill M. A checklist for judging preference-based measures of health related quality of life: learning from psychometrics. Health Econ 1999; 8: 41–5

For peer review only

For peer review only

For peer review only

Figure 1. Development of ePAQ-VAS conceptual framework

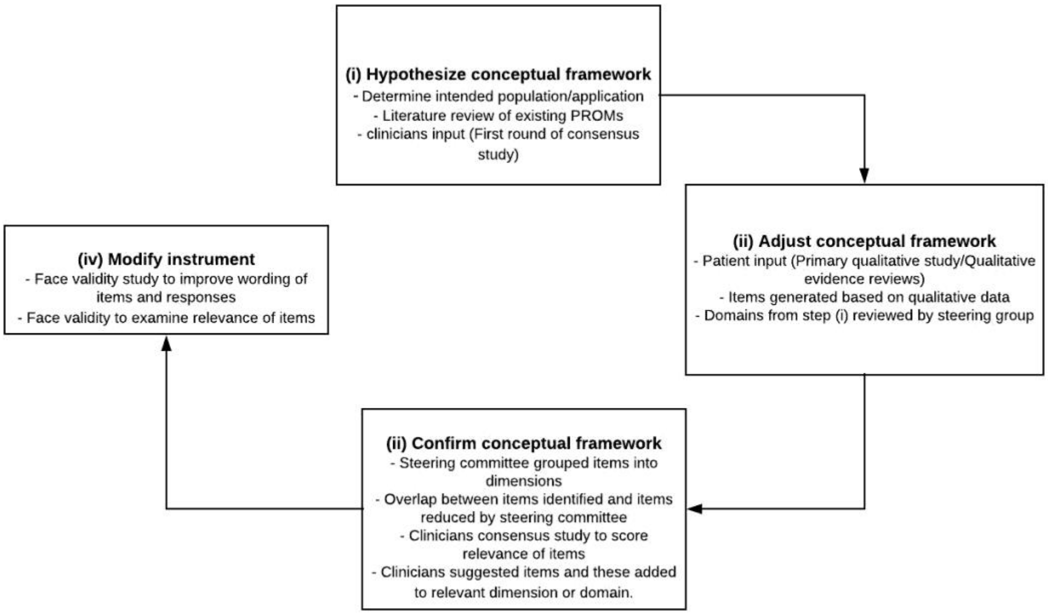

For peer review only

Figure 2. Evidence synthesis to develop of ePAQ-VAS

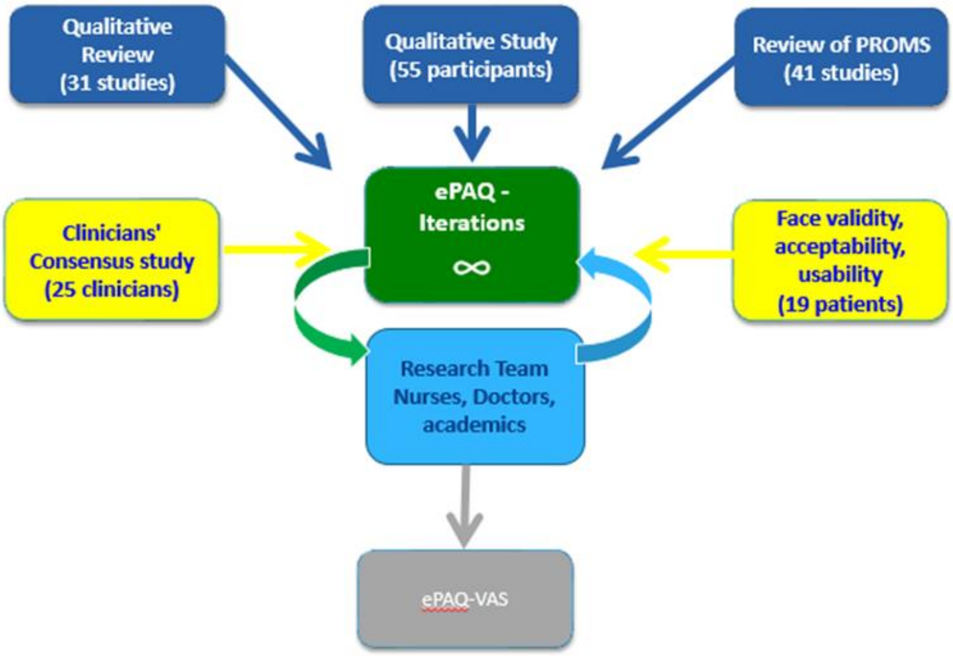

For peer review only

Figure 3. Overview of ePAQ-VAS structure

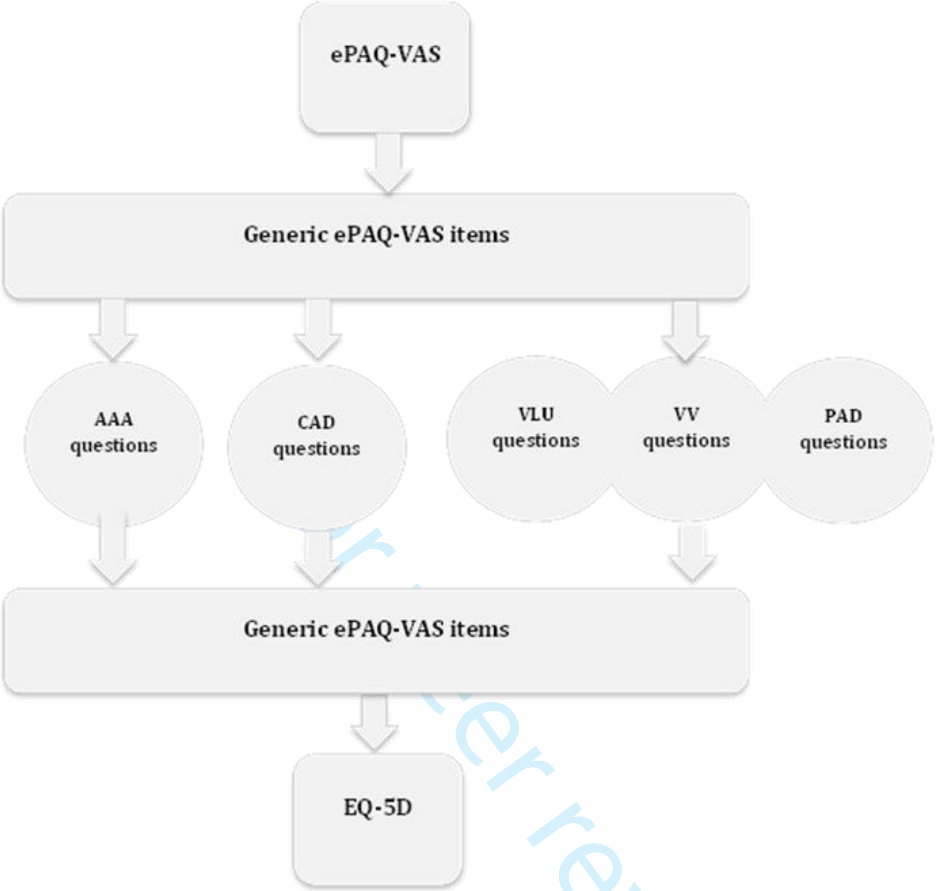

Appendix 1

Table 1: Example of triangulation between PAD PROM review, qualitative evidence synthesis and qualitative interview themes

Qualitative Review Themes	Qualitative Interview Themes	EQ-5D	NHP	SF-6D	SF-8	SF-36	AUSVI QoL	ICQ	PADQ OL	SIPic	WIQ	Vascu QOL
PAD Health Outcomes												
**Pain - location**	Positive	+/-	-	-	-	-	+	-	+	+/-	-	+
**Pain - sensation**	Positive	+/-	+	+	+	+	-	+	+	-	+	-
**Pain - severity**	Positive	+	-	-	-	-	-	-	+/-	+	-	+
**Pain on walking**	Positive	+	+	-	-	-	+	+	+	+	+	+
**Pain at rest**	Positive	+	+	-	-	-	+	+	+	-	-	+
**Mobility - walking ability**	Postive	+/-	+	-	-	+	+	+	+	+	+	+
**Mobility - walking distance**	Positive	+/-	+/-	-	-	+	+	+	-	+	+	+
**Mobility - walking speed**	Positive	+/-	-	-	-	-	-	-	-	-	+	-
**Mobility - stair climbing**	Positive	+/-	+/-	-	-	+	-	+	-	+	+	-
**Mobility -**	Positive	+/-	-	-	-	-	-	-	-	-	-	-

**walking uphill**												
**Bending/ picking up things**	Negative	+/-	-	-	-	+	-	-	-	-	-	-
**Sleep**	Positive	-	+	-	-	+	+	-	-	-	-	--
**Sleep/ pain**	Silence	-	+	-	-	-	+	-	-	-	-	-
**Fatigue**	Negative	-	+	-	-	+	-	-	-	-	-	+
**Sexual functioning**	Negative	-	+	-	-	-	-	-	-	-	-	-
**Comorbiditi es**	Negative	-	-	-	-	-	-	-	-	-	-	-
**Non-healing wounds**	Positive	-	-	-	-	-	+	-	-	-	-	-
**Symptom progression**	Postive	-	-	-	-	-	-	-	-	-	-	-
**Appearance of leg**	Silence	-	-	-	-	-	+	-	-	-	-	-
**Altered sensations in leg**	Silence	-	-	-	-	-	-	-	-	-	-	-
**Foot temperature**	Silence	-	-	-	-	-	-	-	-	-	-	-
Impact on Physical Functioning												
**Personal care**	Positive	+	+	+/-	+/-	+	+	-	+	+	-	+
**Hobbies**	Positive	+	-	-	-	+/-	-	+	+	+	-	+/-
**Exercise**	Negative	+/-	-	-	-	+/-	-	-	-	-	-	+/-
**Daily**	Positive	+/-	+	+	+	+	+	+	+	+	-	+

For peer review only

**activities**												
Impact on Social Functioning												
**Social support**	Positive	-	+	-	-	-	-	-	-	-	-	-
**Social activities**	Positive	-	+	+	+	+	+	+	+	+	-	+
**Role**	Positive	-	+/-	+	+	-	-	+	+	-	-	+/-
**Isolation**	Positive	-	+	+/-	+/-	-	+	-	+	-	-	-
**Independen ce/ burden**	Positive	-	+	+	-	+/-	-	+	-	-	-	+
Impact on Psychologica l Function												
**Loss/ grief**	Positive	-	-	-	-	+/-	+	-	+	-	-	
**Unfulfilled desire**	Negative	-	+	-	-	-	-	-	+	-	-	
**Illness beliefs**	Positive	-	+	-	-	-	-	-	+	-	-	
**Feeling old**	Positive	-	-	-	-	-	-	-	-	-	-	
**Feeling abnormal**	Positive	-	-	-	-	-	-	-	+	-	-	
**Emotions/ mood**	Positive	-	+/-	+	+	+	+	+	+	+	-	
**Depression**	Positive	-	+	+	+	+	+	+	+	-	-	
**Fears**	Positive	-	-	-	-	+	+	+	+	-	-	
**Concentrati on/ intellectual capacity**		-	-	-	-	-	-	-	+	-	-	

For peer review only

**Treatment beliefs**		-	-	-	-	-		-	+	-	-	
**Uncertainty over the future**	Partial agreement	-	-	-	-	-	-	+/-	+	-	-	
Key:<br>. = silence<br>-/+ = partial agreement<br>- = dissonance<br>+ = agreement												

For peer review only

BMJ Open

Mixed methods study to develop the content validity and the conceptual framework of the electronic patient-reported outcome measure for vascular conditions

Journal:	*BMJ Open*
Manuscript ID	bmjopen-2019-034154.R1
Article Type:	Original research
Date Submitted by the Author:	18-Mar-2020
Complete List of Authors:	Aber, Ahmed; The University of Sheffield, ScHARR<br>Phillips, Patrick<br>Lumley, Elizabeth<br>Radley, Stephen; Sheffield Teaching Hospitals NHS Foundation Trust<br>Thomas, Steve; The University of Sheffield, School of Health and Related Research (ScHARR)<br>Nawaz, Shah; Sheffield Teaching Hospitals NHS Foundation Trust, Sheffield Vascular Institute<br>Jones, Georgina; Leeds Beckett University Faculty of Health and Social Sciences,<br>Michaels, Jonathan; University of Sheffield
**Primary Subject Heading**:	Surgery
Secondary Subject Heading:	Health policy, Health services research, Qualitative research, Surgery, Cardiovascular medicine
Keywords:	Patient reported outcome measures, Vascular surgery < SURGERY, electronic health record (EHR), Quality in health care < HEALTH SERVICES ADMINISTRATION & MANAGEMENT, QUALITATIVE RESEARCH, Mixed methods

SCHOLARONE™
Manuscripts

I, the Submitting Author has the right to grant and does grant on behalf of all authors of the Work (as defined in the below author licence), an exclusive licence and/or a non-exclusive licence for contributions from authors who are: i) UK Crown employees; ii) where BMJ has agreed a CC-BY licence shall apply, and/or iii) in accordance with the terms applicable for US Federal Government officers or employees acting as part of their official duties; on a worldwide, perpetual, irrevocable, royalty-free basis to BMJ Publishing Group Ltd ("BMJ") its licensees and where the relevant Journal is co-owned by BMJ to the co-owners of the Journal, to publish the Work in this journal and any other BMJ products and to exploit all rights, as set out in our licence.

The Submitting Author accepts and understands that any supply made under these terms is made by BMJ to the Submitting Author unless you are acting as an employee on behalf of your employer or a postgraduate student of an affiliated institution which is paying any applicable article publishing charge ("APC") for Open Access articles. Where the Submitting Author wishes to make the Work available on an Open Access basis (and intends to pay the relevant APC), the terms of reuse of such Open Access shall be governed by a Creative Commons licence – details of these licences and which Creative Commons licence will apply to this Work are set out in our licence referred to above.

Other than as permitted in any relevant BMJ Author's Self Archiving Policies, I confirm this Work has not been accepted for publication elsewhere, is not being considered for publication elsewhere and does not duplicate material already published. I confirm all authors consent to publication of this Work and authorise the granting of this licence.

For peer review only

Mixed methods study to develop the content validity and the conceptual framework of the electronic patient-reported outcome measure for vascular conditions

1) Authors: Ahmed Aber[a], Patrick Phillips[a], Elizabeth Lumley[a], Stephen Radley[b], Steve Thomas[c], Shah Nawaz[c], Georgina Jones[c], Jonathan Michaels[a]

Ahmed Aber, University of Sheffield, a.aber@sheffield.ac.uk
Patrick Phillips, University of Sheffield, p.phillips@sheffield.ac.uk
Elizabeth Lumley, University of Sheffield, e.lumley@sheffield.ac.uk
Stephen Radley, Sheffield Teaching Hospitals, Stephen.Radley@sth.nhs.uk
Steve Thomas, Sheffield Teaching Hospitals, s.m.thomas@sheffield.ac.uk
Shah Nawaz, Sheffield Teaching Hospitals, shah.nawaz@sth.nhs.uk
Georgina Jones, Leeds Beckett University, g.l.jones@leedsbeckett.ac.uk
Jonathan Michaels, University of Sheffield, j.michaels@sheffield.ac.uk

2) [a]Institution: School of Health and Related Research, University of Sheffield, Sheffield
[b]Institution: Jessop Wing, Sheffield Teaching Hospitals, Sheffield
[c]Institution: Sheffield Vascular Institute, Sheffield Teaching Hospitals, Sheffield
[d]Institution: Department of Psychology, School of Social Sciences, Leeds Beckett University, Leeds

3) *Corresponding author: Ahmed Aber, Regent Court, 30 Regent Street, School of Health and Related Research, University of Sheffield, Sheffield, S1 4DA.
E-mail: a.aber@sheffield.ac.uk.
Tel: +44 (0) 114 222 4029
Fax: +44 (0) 114 222 0740

4) Category for manuscript submission: Original Research

5) Source of funding for research and publication: This manuscript presents independent research funded by the National Institute for Health Research (NIHR) under the Programme Grants for Applied Research (RP-PG-1210-12009).
The views expressed are those of the authors and not necessarily those of the NHS, the NIHR or the Department of Health.

6) The authors thank the UK Vascular PROMs Group and Cardiovascular Research Patient Panel at Sheffield Teaching Hospitals NHS Foundation Trust.

7) Conflict of interest: Stephen Radley is a shareholder in ePAQ Systems Ltd, an NHS spin-out technology company, the majority shareholder being Sheffield Teaching Hospitals. The other authors have no conflict of interest.

For peer review only

UK Vascular PROMs Group:
Ms. Alison Abbott, Vascular Clinical Nurse Specialist.
Mr. Ayman Badawy, Vascular Surgery Associate Specialist.
Mr. Nasim Akhtar, Vascular Consultant Surgeon.
Mr. Faisal, Alam, Vascular Consultant Surgeon.
Mr. Wissam Al-Jundi, Vascular Consultant Surgeon.
Mr. Matthew Armon, Vascular Consultant Surgeon.
Mr. Lukla Biasi, Vascular Consultant Surgeon.
Professor Matt Bown, Vascular Consultant Surgeon.
Professor Bruce Campbell, Vascular Consultant surgeon.
Mr. Tommaso Donati, Vascular Consultant Surgeon.
Mr. Tony Fox, Vascular Consultant Surgeon.
Mr. Andrew Gordon, Vascular Consultant Surgeon.
Professor Linda Hands, Vascular Consultant Surgeon.
Mr. Gary Hicken, Vascular Consultant Surgeon.
Mr. Peter Holt, Vascular Consultant Surgeon.
Dr. Shelley Jackson, General Practioner.
Ms. Brenda King, Specialist Nurse.
Ms. Talia Lea, Vascular Clinical Nurse Specialist.
Mr. Greg McMahon, Vascular Consultant Surgeon.
Mr. John Mosley, Vascular Consultant Surgeon.
Mr. Sanjay Patel, Vascular Consultant Surgeon.
Dr. Gaynor Radley, General Practioner.
Mr. Harjeet Rayt, Vascular Consultant Surgeon.
Professor Rob Sayers, Vascular Consultant Surgeon.
Mr. Paul Tisi, Vascular Consultant Surgeon.

Patient and Public involvement:

The research question and output was developed in consultation with patients and public. The authors thank the Cardiovascular Research Patient Panel at Sheffield Teaching Hospitals NHS Foundation Trust. The aim of the research was to develop a patient focused outcome measure. In this process patients were recruited for two qualitative studies to ensure content validity and face validity of this tool. Patients were involved in every stage of the development of the study. The developed electronic patient assessment questionnaire for vascular conditions (ePAQ-VAS) have been used by patients in a clinical study and there are plans for regular clinical use. The results will also be disseminated in relevant meetings and among patient groups.

Keywords:
Vascular disease, patient-reported outcome measures, quality of life, content validity, qualitative research, electronic questionnaire.

For peer review only

Abstract

Objective:

The aim of this paper is to describe the stages undertaken to generate the items and conceptual framework of a new electronic personal assessment questionnaire for vascular conditions.

Design:

A mixed methods study: a clinician survey followed by quantitative systematic reviews to identify validated patient reported outcome measures (PROMs). A qualitative study of patients and systematic reviews of the qualitative evidence were used to develop this new PROMs. This was followed by a quantitative clinicians' consensus study and finally, a qualitative face validity study with patients.

Participants:

Vascular patients participated in the primary qualitative study and the face validity study. In the qualitative study fifty-five patients were interviewed and for the face validity nineteen patients gave feedback. Twelve clinicians completed the survey and thirteen completed two cycles of the clinicians' consensus study.

Results:

The items and scales in the electronic personal assessment questionnaire for vascular conditions (ePAQ-VAS) were generated based on the results of five systematic reviews evaluating existing PROMs for possible inclusion in ePAQ-VAS, five systematic reviews of qualitative evidence, a primary qualitative study involving 55 patients and clinicians' input. One hundred and sixty-eight items were initially generated; of which 59 were eliminated by the expert panel due to repetition. The instrument was divided into one generic, and three disease-specific sections (abdominal aortic aneurysm (AAA), carotid artery disease (CAD), and lower limb vascular conditions). In each section, items were grouped together into putative scales. Fifty-five items were grouped across eight scales; the remaining items were kept as individual items, because of relevance to service users.

Conclusions:

This multi-dimensional electronic questionnaire covers the most common vascular conditions. This is particularly important for patients presenting with mixed symptoms or multiple conditions. This tool captures symptomatology, HRQoL and other clinically relevant data, such as experience with services and co-morbidities.

For peer review only

Strengths and limitations of the study

- This electronic patient assessment questionnaire for vascular conditions was developed with input from patients and clinicians. The themes generated from previously published five comprehensive qualitative reviews and a qualitative study of vascular patients were used to develop the items.
- Vascular clinicians were surveyed to ensure clinically relevant conditions and questions were included.

The burden of questionnaire is its main limitation; however, by providing strict skipping rules only relevant questions are presented to patients. The face validity study examined the clarity and relevance of the items, however, the comprehensiveness this PROMs was not assessed.

For peer review only

Introduction

Vascular conditions can cause problems throughout the body; epidemiological studies suggest that both venous and arterial diseases are very common **(1, 2)**. It therefore makes sense to assess individuals with vascular disease holistically; investigating not only existing or potential manifestations of vascular disease, but also the impact of conditions on health-related quality of life. PROMs are questionnaires or instruments, designed to elicit information directly from the patient and can be used as part of such an assessment.

Validity and reliability are integral to developing or selecting a PROMs. A key aspect of validity is *content validity,* and international guidelines including the U.S. Food and Drug Administration (FDA) guidance stress the importance of this psychometric property **(3)**.

Many generic and condition specific PROMs have been adopted to examine impact of vascular conditions on patients and measure outcomes. This is despite a lack of evidence that they have been developed and evaluated in-line with accepted guidelines; in addition, these instruments are rarely used or formally evaluated in routine patient assessment in day to day clinical practice. We conducted scoping searches and informal discussions with vascular clinicians to identify any existing PROMs, however, these preliminary stages in the research process suggested an absence of valid and reliable PROMs for use in vascular populations.

In this paper, we report the stages in developing an electronic personal assessment questionnaire for vascular patients (ePAQ-VAS). This includes:

(i) Identifying the main vascular conditions to be included in this electronic measure based on a survey of clinicians treating vascular disease.

(ii) Developing a hypothesized framework for the sections for different disease categories based on the previous systematic reviews that identified PROMs used in patients with abdominal aortic aneurysm (AAA), carotid artery disease (CAD), peripheral arterial disease (PAD), venous leg ulcers (VLU) and varicose veins (VV) **(4-8).**

(iii) Developing the items within each section of ePAQ-VAS based on qualitative systematic reviews **(9-12),** and a primary qualitative study **(13).**

For peer review only

(iv) A consensus study with clinicians to rate the relevance of included items, and to add items to ePAQ-VAS based on the opinion of vascular surgeons, radiologists and nurses.

(v) A face validity study with vascular patients to examine the clarity and relevance of the items within ePAQ-VAS.

The aim of these steps was to develop a single electronic instrument covering most vascular conditions in line with international guidance **(3)**. The conceptual framework and items were developed in a way to ensure this assessment tool can be used in patients with mixed symptoms and multiple vascular conditions.

Methods

Clinicians involved in the care of vascular patients were invited to identify the common vascular conditions treated by vascular surgeons and vascular specialists. They were asked to list the key issues, symptoms and the impact of these conditions on patients suffering with these diseases. Data from this round were used to inform qualitative evidence synthesis.

The conceptual framework of ePAQ-VAS was based on primary qualitative interviews with vascular patients, input from clinicians, systematic reviews examining the validity of existing PROMs, and qualitative reviews of the impact of vascular diseases on quality of life. Ethical approval was obtained from Yorkshire & Humber NRES committee– Bradford Leeds (REC Number: 14/YH/1117) on 25.09.2014. Figure 1. Illustrates the process used to develop ePAQ-VAS in accordance to FDA guidelines **(3)**.

For peer review only

Figure 1. Development of ePAQ-VAS conceptual framework

Insert figure 1 here

Systematic Reviews to Identify and Appraise Existing PROMs

Systematic searches were conducted of bibliographic databases including CINAHL via EBSCO, MEDLINE and MEDLINE in Process via Ovid, EMBASE via Ovid, PsycINFO via Ovid, Social Science Citation Index/Science Citation Index via Web of Science (Thomson Reuters), and Proquest dissertations and theses. PROMs were included where there was evidence that they had undergone some form of psychometric evaluation that would allow the validity, reliability and responsiveness of the PROMs to be assessed. Included PROMs were categorised per type, (generic or condition specific) and the vascular population(s) in which they had been validated. Quality assessment **(3, 14)** was conducted to identify high quality existing PROMs for possible direct inclusion in ePAQ-VAS, or to be used as a basis to inform the qualitative evidence synthesis. For further information about the appraisal criteria to examine the robustness of the psychometric analysis and samples of search strategies please see the supplementary materials.

Primary qualitative study

Semi-structured interviews were conducted with 55 vascular patients from Sheffield Teaching Hospitals NHS Foundation Trust (STH) following purposeful sampling to ensure a range of participants of different age and gender, at different stages of treatment and covering the main five vascular conditions (AAA, PAD, CAD, VLU, VV). A consultant vascular surgeon or specialist nurse approached each patient either in clinic or over the telephone to explain about the project and ask if the patient would be interested in participating in the study. If the initial approach was by the clinician in clinic, the researcher would then speak to the patient and give further information about the project including a participant information sheet (PIS) before taking contact details. For those patients that were first contacted over the phone the clinician would then gain verbal consent to pass on their contact details to a researcher. Copies of the PIS were sent out through the mail to those who had not been initially approached in

For peer review only

clinic. The researcher gave at least 24 hours for the patient to read through the PIS and consider the information before contacting each person by telephone to ask if they would be interested in participating in an interview. If they were interested in taking part a date and time was agreed for a researcher to visit the participant at home to carry out an interview. Questions were asked about the signs, symptoms, and impact of the condition on function and lifestyle. On the day of the interview the trained qualitative researcher checked the participant understood the PIS and took informed written consent. Field notes were taken to aid interpretation of the interview data. Each interview was recorded and transcribed verbatim. Personal details were removed from the transcript to enhance participant anonymity. The interview transcripts were typed and uploaded into NVIVO 11 (QSR International, Warrington, UK) for management and analysis.

Systematic reviews of the qualitative evidence

Systematic searches of the following databases; CINAHL via EBSCO, MEDLINE and MEDLINE in Process via Ovid, EMBASE via Ovid, PsycINFO via Ovid, Social Science Citation Index/Science Citation Index via Web of Science (Thomson Reuters), and Proquest dissertations were conducted to identify existing qualitative research detailing vascular patients' experience of living with AAA, PAD, CAD, VLU, and VV. For further samples of search strategies please see the supplementary materials.

Analysis of the qualitative evidence

Qualitative data from the primary study and each of the systematic qualitative reviews were analysed separately. Framework analysis was used to analyse the interviews **(15).** This analysis includes five stages,

- the first involved familiarization by reading of the transcripts and reading the primary data.
- The second stage involved identification of a thematic framework; the thematic framework was based either on clinical opinion for areas with no valid PROMs, such as AAA and CAD, or a combination of clinical opinion and, when available, the scales of PROMs with good content validity.

For peer review only

- In the third stage the data was coded and indexed by applying the thematic framework to the whole data set until saturation was achieved. Anindependent researcher checked all the themes that were identified, and differences in were discussed and adjusted involving a third senior author (GJ)
- At the fourth stage, a framework matrix was created by arranging the data per the thematic references.
- finally mapping and interpretation, including examining patterns within the data and associations with it.

Clinicians input and consensus exercise

Twenty-three clinicians involved in the care and management of patients with vascular conditions were invited to a survey to list the most common vascular conditions managed by them and to list the key issues, symptoms and the impact of these diseases on patients. Data from this round were used to inform qualitative evidence synthesis.

Different group of clinicians involved in the care of vascular patients were invited to a consensus study to score the relevance of items (questions) in the provisional version of ePAQ-VAS. In total, thirty clinicians including vascular surgeons, interventional radiologists, vascular nurses, physiotherapists and occupational therapists were invited. Participants were asked to rate the appropriateness of each question on a five-point Likert scale of “Strongly disagree” (=0) to ‘Strongly agree’ (=4). This process was repeated, and members of the clinicians’ panel were presented with the aggregate findings of the previous round, and again asked to score each question. This process aimed to examine the relevance of each item from the clinicians’ perspective and to identify any new items suggested by the clinicians **(16)**.

Developing scales and items

The ePAQ development team (AA, EL, PP, GJ, SR) employed an iterative process, incorporating evidence from the systematic reviews, qualitative study, and the clinicians’ consensus study. In line with the FDA guidance **(3)**, items (questions) were

For peer review only

developed from the qualitative data using the following three steps: interpretation, translation, and triangulation of themes.

Interpretation involved familiarisation with the language used in the primary data included in the synthesis. This enabled translation of descriptions of apparently diverse issues affecting vascular patients into a single set of harmonised themes. The resulting themes were used to develop the items for ePAQ-VAS. The items were grouped into sections, and each section, further divided into scales consisting of a connected group of items. Triangulation was performed across evidence sources to ensure the items comprehensively covered all issues of importance to patients with AAA, PAD, CAD, VLU, and VV.

Face validity of ePAQ-VAS

A second phase of semi-structured patient interviews was conducted by (EL, PP) with 19 participants, purposefully sampled from the vascular populations previously described. This sample included patients with AAA, CAD, PAD, VLU and VVs. ePAQ-VAS (version 1) was presented to these patients and a focused interview was conducted to investigate vascular patients' perceptions of the questionnaire in its entirety as well as the relevant items to the individual being interviewed. Questions were asked under the following headings of:

- overall impressions,
- clarity,
- relevance and emotional response.

Interviews were audio taped, transcribed and analysed. A pragmatic approach was used for the analysis, with comments collated and presented back to the working group who made consensus decisions on revisions to ePAQ-VAS. Written consent was obtained from the participants.

Results

In total twelve clinicians completed the first survey and identified PAD, AAA, VLU, VV and CAD as the most common vascular conditions treated by them. They listed

For peer review only

common issues such as pain on walking, rest pain, reduced mobility or lack of mobility for patients with PAD, no physical symptoms for those with AAA but need for multidisciplinary approach to manage these patients. For patients with VLU, the main issues included burning pain, recurrence and healing; for patients suffering with VV, skin changes, appearance of leg and ulcer as well as ache were the main issues raised. The clinicians felt the key issue for patients with CAD was identifying patients benefiting from intervention and reducing the risk of stroke. The result from this survey was used to inform the analysis of qualitative data used to develop ePAQ-VAS.

Systematic reviews and assessment of psychometric evaluation were conducted for PROMs validated for use in PAD, AAA, VLU, VV and CAD. A total of 33 PROMs that had undergone some form of validation were identified in 41 studies **(Table 1.)**.

Condition	Number of Citations	Number of Included Papers	Results	Conclusions
AAA	1232	3	4 validated PROMs identified –1 generic, 1 vascular generic and 2 condition-specific	This review has highlighted a gap in the evidence for validated PROMs in AAA. Due to a lack of rigorous psychometric testing.
CAD	1670	5	6 validated PROMs identified – 4 generic and 2 condition-specific	There was a lack of validated PROMs to measure outcomes for CAS patients.
PAD	6981	14	13 validated PROMs identified – 6 generic and 7 condition-specific	VascQol was the most psychometrically robust instrument.
VV	3879	7	3 validated PROMs identified – 1 generic and 2 condition-specific	Aberdeen Varicose Vein Questionnaire is the most psychometrically robust disease specific PROMs

For peer review only

				for use with VV patients.
VLU	3879	6	7 validated PROMs identified – 3 generic and 4 condition-specific	The most valid and reliable condition specific PROMs was VLU-QOL.
Abbreviations: Abdominal aortic aneurysm (AAA), Carotid artery disease (CAD), Peripheral arterial disease (PAD), Venous leg ulcer (VLU), Varicose veins (VV).				

Table 1: Results from the systematic reviews of psychometric evaluation of vascular PROMs

No PROMs were identified that had undergone sufficiently rigorous development and validation to suggest that they were suitable for direct use in ePAQ-VAS, the details of these reviews have been reported previously **(4-8).** Where evidence existed, this fell short of required standards **(3,14)**. For instance, the review investigating VV PROMs **(4)** found some evidence for, and discussion of, content validity in relation to the Aberdeen varicose vein questionnaire (AVVQ) and suggest that it is the most appropriate existing condition specific measure for use in a VV population. However, item generation for this PROMs involved a literature review and assessment by clinicians of relevance of included items with no direct involvement of patients, therefore suggesting a deficiency in terms of content validity **(17)**. The scales from these reviews were used to provide a framework for the systematic qualitative reviews and the primary qualitative study **(5, 9-13)**.

In total 111 patients were approached but only fifty-five patients (69.1% male) were interviewed about their experience of living with vascular disease, ages ranged from 35-77 years. For further information about the study participants please see table 2.

For peer review only

	AAA	CAD	PAD	VV	VLU	TOTAL
Gender n(%):						
Male	10(77)	5(56)	11(79)	5(50)	8(80)	39(70)
Female	3	4	3	5	2	17
Age Range	53-87	52-86	47-82	35-77	47-84	35-87
(mean)	(72)	missing	(69)	(50)	(59)	missing

Table 2: Participant characteristics of the primary qualitative study

Six overarching themes relating to the impact of the five vascular conditions were identified. These were symptoms (including pain), impact on physical function, social impact, psychological impact, financial impact and lifestyle. Pain and mobility were the most commonly reported themes by participants with PAD. The extent to which they impacted daily living was dependent on the severity of the disease, age expectations and social support. Fear of symptoms worsening and future amputation had a significant impact on daily living.

Most participants with AAA reported having no physical symptoms, a small number of participants reported abdominal pain and pain in their legs. Uncertainty, anxiety and fear of sudden death had the most impact on their quality of life. This was similar for patients with CAD who reported few lasting symptoms since the majority had what they described as a “mini-stroke”. However, CAD patients reported the widest range of signs and symptoms, with nine different symptoms. This condition had the least impact on physical and social function, although psychologically it caused a sense of worry and anxiety. This was mainly caused by fear of having a major stroke.

Pain was the most common issue reported by patients with VVs, other issues included swelling of the legs and the impact of this on mobility. The perceived unpleasant appearance of the VV seemed to have had the greatest psychological impact, and was described by many of the participants. The impact of VLU on daily living and quality of life differed within the group that was interviewed. For some there were no major issues and having a VLU was accepted as part of their life, with the hope that it would heal eventually. For others, there was a far more significant effect with reports of severe sharp pain that significantly reduced their quality of life. This had a bearing on

For peer review only

people's mobility and their ability, or desire, to go out and socialise. Sleep was also disturbed due to pain. The progression of VLU had resulted in participants suffering for long periods of time. In addition, the non-healing or reoccurring nature of the condition had a significant impact for many. VLU appeared to have a significant psychological impact causing a high degree of distress for some patients. Summary results are shown in **Table 3**.

Condition	Sample size	Key Findings
AAA	13	No physical symptoms, a small number of participants reported abdominal pain and pain in their legs. Uncertainty, anxiety and fear of rupture and death appeared to impact most greatly on people's QoL.
CAD	9	This condition seemed to have had the least impact on physical and social function, although psychologically it created a sense of worry and anxiety for some participants. The main reported outcome was fear of having a major stroke.
PAD	14	Pain and mobility were the most commonly reported themes. The extent to which they impacted on QoL was associated with the severity, age expectations and social support. Fear of the symptoms worsening and amputation was evident.
VV	10	VV do not appear to have had a major impact on overall QoL for most the participants. Pain was the most common issue. The perceived unpleasant appearance of the VV seemed to have the greatest psychological impact. Many of the participants had had their VV for very long periods of time, often just "putting up with it'' for numerous years before seeking help.
VLU	10	The impact of VLU on QoL differed within the group. For some there were no major issues and having a VLU was accepted as part of their current life, with the hope that it would heal eventually. For others, there was a far more significant effect. Pain was quite severe for some participants leading to a significantly reduced QoL. VLU appeared to have a significant psychological impact causing a high degree of distress for some.
Abbreviations: Abdominal aortic aneurysm (AAA), Carotid artery disease (CAD), Peripheral arterial disease (PAD), Venous leg ulcer (VLU), Varicose veins (VV).		

Table 3: Findings from the primary qualitative study with vascular patients

For peer review only

Identified signs, symptoms and impact of the conditions were then mapped and tabulated to see which themes were relevant to which condition, and where the similarities and differences lay **(Table 4.)**.

	**PAD**	**AAA**	**CAD**	**VV**	**VLU**
**Symptoms**					
No symptoms		x	x		
Pain	x	x	x	x	x
Neck pain			x		
Leg pain	x	x	x	x	x
Abdominal pain	x	x			
Arm pain	x				
Cramp/ aching	x	x		x	x
Burning sensation					x
Pain severity	x	x	x	x	x
Pain on walking	x	x		x	x
Pain at rest	x			x	
Pain when standing				x	x
Mobility	x	x	x	x	x
Distance	x	x		x	x
Speed	x	x			
Stairs/ slopes	x	x			
Non-healing wounds	x				x
Co-morbidities	x	x	x		x
Progression of symptoms	x	x		x	x
Sleep	x		x	x	x
Swelling			x	x	
Loss of balance			x		
Confusion			x		
**Impact on physical functioning**					
Hobbies	x			x	x

Exercise	x	x		x	x
Daily activities	x				x
**Social impact**					
Travel	x	x	x		
Social activities	x	x		x	x
Social support	x	x	x		
**Psychological impact**					
Anxiety	x	x	x	x	x
Depression	x	x			x
Feelings of loss	x			x	x
Health expectations	x	x	x	x	x
Unsightly appearance				x	
Feeling self-conscious	x			x	x
Fear of worsening symptoms	x	x	x	x	x
Fear of rupture death		x			
Fear of amputation				x	x
Fear of stroke			x		
**Financial impact**					
Income	x	x			x
Time off work				x	x
**Lifestyle**					
Smoking	x	x	x	x	x
Exercise	x	x	x	x	
Diet	x	x	x		x
Weight				x	x
Abbreviations: Abdominal aortic aneurysm (AAA), Carotid artery disease (CAD), Peripheral arterial disease (PAD), Venous leg ulcer (VLU), Varicose veins (VV).					

Table 4: Map of symptoms and Quality of life concepts across five conditions.

For peer review only

A total of 31 studies were included across the five reviews of existing qualitative research **(6, 10-13)**. A short summary of the main themes to emerge for each condition is shown in **Table 5**.

Condition	Numbers of Citations	Number of Included Studies	Key Themes
AAA	315	3	Anxiety and *lack* of physical symptoms.
CAD	964	3	Symptoms, psychological and social impact, risk and service experience.
PAD	973	9	Pain, compromised physical function, impact on social life.
VV	1804	3	Adaptation – coping strategies employed to limit various impacts, appearance of VV.
VLU	1804	13	Pain, odour and exudate - impact on sleep, mobility and mood.
Abbreviations: Abdominal aortic aneurysm (AAA), Carotid artery disease (CAD), Peripheral arterial disease (PAD), Venous leg ulcer (VLU), Varicose veins (VV).			

Table 5: Results from Qualitative Reviews examining the impact of the major vascular conditions on quality of life

The themes from the first round of the clinicians' consensus study, as well as scales of identified PROMs, were used to inform the framework analysis of the qualitative data. Items from existing PROMs were then mapped against emerging themes from the qualitative study, and the qualitative review synthesis for each condition, to explore which PROMs items or scales captured themes deemed to be the most pertinent to patients. A triangulation approach was followed, whereby researchers evaluated whether the concepts were the same (agreement), offered similar concepts (partial agreement), were in contradiction (dissonance) or were not present (silence). An example of this triangulation approach is provided in **the supplementary material**. The results of the triangulation study were only used to group symptoms together and avoid repetition. No items were deleted based on the triangulation.

For peer review only

The ePAQ-VAS development team used the findings from the triangulation for AAA, PAD, VV, VLU and CAD to develop themes for distinct sections relevant for each of these vascular conditions. The primary qualitative data were used to create each item. Items were then grouped into sections and within each section there were scales consisting of items that measured the same latent variable such as anxiety related to the diagnosis of AAA. The results of the clinicians' consensus study were considered to add further items to the relevant sections **(Table 6)**.

The items of ePAQ-VAS were arranged into four sections; generic, AAA, CAD and lower limb (LL) vascular conditions. A single LL section was developed as common themes were identified for conditions affecting the legs, regardless of whether the underlying pathology was venous or arterial. An inclusive approach to development was used and a comprehensive questionnaire was produced with 168 questions. (See Figure 2 for an overview of the process to develop ePAQ-VAS).

For peer review

**Section**	**Scale**		**Clinicians' consensus Study**	**Most valid Condition Specific PROMs**			**Qualitative study**					**Qualitative Reviews**					**Question Text**
				**VLU-QOL**	**PADQOL**	**AVVQ**	**AAA**	**CAD**	**VV**	**VLU**	**PAD**	**AAA**	**CAD**	**VV**	**VLU**	**PAD**	
**Generic**		**Pain**	**4**	**X**	**X**	**X**	**X**	**X**	**X**	**X**	**X**	**X**	**X**	**X**	**X**	**X**	**Do you suffer with any pain?**
**Generic**		**Pain**	**4**	**X**	**-**	**-**	**X**	**X**	**X**	**X**	**X**	**X**	**X**	**X**	**X**	**X**	**Use the below Image and click on body parts where you experience pain or discomfort.**
**Generic**		**Pain**	**-**	**X**	**-**	**-**	**X**	**X**	**X**	**X**	**X**	**X**	**X**	**X**	**X**	**X**	**Please use your own words to describe this problem.**
**Generic**		**Pain**	**4**	**X**	**X**	**X**	**X**	**X**	**X**	**X**	**X**	**X**	**X**	**X**	**X**	**X**	**How often do you experience a significant amount of pain?**
**Generic**		**Pain**	**4**	**-**	**-**	**-**	**X**	**X**	**X**	**X**	**X**	**X**	**X**	**X**	**X**	**X**	**How much do problems caused by pain affect your overall enjoyment of life?**

Generic		Sensation	4	-	-	-		X	X		X	X	X	X		X	Do you experience any numbness or pins and needles in any part of your body?
Generic		Sensation	4	-	-	-		X	X		X	X	X	X		X	Please use the image below to select where you experience sensation change in your body.
Generic		Sensation	-	-	-	-		X	X		X	X	X	X		X	Please use your own words to describe this problem.
Generic		Sensation	4	-	-	-		X	X		X	X	X	X		X	How often do you experience numbness or pins and needles?
Generic		Sensation	4	-	-	-		X	X		X	X	X	X		X	How much do problems caused by numbness or pins & needles affect your overall enjoyment of life?
Generic		Weakness	4	-	-	-						X	X			X	Do you have any loss of strength or weakness in any part of your body?
Generic		Weakness	3	-	-	-						X	X			X	Please use the image below to indicate the areas where you experience any physical weakness.
Generic		Weakness	-	-	-	-						X	X			X	Please use your own words to describe this problem.
Generic		Weakness	4	-	-	-						X	X			X	How often do you experience loss of strength or weakness?
Generic		Weakness	3	-	-	-						X	X			X	How much do problems caused by weakness affect your overall enjoyment of life?
Carotid		Anxiety	4					X					X				Do you worry about having a stroke?
Carotid		Anxiety	3					X					X				Does carotid artery disease make you feel anxious?
Carotid		Anxiety	New item														Are you worried about your health getting worse because of carotid artery disease?
Carotid		Anxiety	New item														Are you worried about losing your independence because of carotid artery disease?
Carotid		Symptoms	2					X									Do you have any problems with maintaining your balance?
Carotid		Symptoms	2					X									Do you suffer with any problems with your memory? (E.g. Forgetting or losing things)
Carotid		Symptoms	4					X					X				Have you had any problems with your speech? (E.g. Slurring your words or not being able to speak or say things properly)
Carotid		Symptoms	New item														Do you have any problems with swallowing food?
Carotid		Symptoms	4					X					X				Have you had any problems with partial or complete loss of vision in either of your eyes?
Carotid		Symptoms	4					X					X				How would you describe any loss of vision in your RIGHT eye?
Carotid		Symptoms	4					X					X				How would you describe any loss of vision in your LEFT eye?
Carotid		ADL	3					X					X				How much do problems caused by carotid artery disease (anxiety associated with diagnosis or physical symptoms) affect your overall enjoyment of life?

Carotid		ADL	4					X					X				How much do problems caused by carotid artery disease (e.g. mini-stroke or stroke memory balance speech visual or other related issues) affect your physical activities such as exercise walking or running?
Carotid		ADL	3					X					X				How much do problems caused by carotid artery disease (e.g. mini-stroke or stroke memory balance speech visual or other related issues) affect your ability to undertake personal roles and responsibilities such as caring for others study or work?
Carotid		ADL	4					X					X				How much do problems caused by carotid artery disease (e.g. mini-stroke or stroke memory balance speech visual or other related issues) affect your ability to look after yourself?
Carotid		ADL	3					X					X				How much do problems caused by carotid artery disease (e.g. mini-stroke or stroke memory balance speech visual or other related issues) affect your social activities such as visiting friends and family?
Carotid		ADL	2					X					X				How much do problems caused by carotid artery disease visual or other related issues) affect your mood?
AAA		Symptoms	4				X					X					Do you have any abdominal (tummy) pain?
AAA		Symptoms	New item (3)														Do you experience a throbbing feeling in your abdomen (tummy)?
AAA		Anxiety	4				X					X					Do you worry about aortic aneurysm?
AAA		Anxiety	4				X					X					Do you worry about any symptoms you experience that may be caused by aortic aneurysm?
AAA		Anxiety	4				X					X					Do you worry about possible increase in the size of your aneurysm?
AAA		Anxiety	4				X					X					Do you fear sudden death or rupture of your aortic aneurysm?
AAA		Anxiety	4				X					X					Do you avoid physical exertion because of having an aortic aneurysm?
AAA		Anxiety	3				X					X					Do you avoid travelling independently because of aortic aneurysm?
AAA		ADL	3									X					How much do problems caused by aortic aneurysm affect your overall enjoyment of life?
AAA		ADL	4				X					X					How much does aortic aneurysm affect your physical activities? (E.g. Exercise walking or going out)
AAA		ADL	3				X					X					How much does aortic aneurysm affect your ability to undertake personal roles and responsibilities? (E.g. Caring for others study or work?
AAA		ADL	3									X					How much do you feel aortic aneurysm affects your ability to look after yourself? (E.g. Rest wash toilet or feed yourself)
AAA		ADL	4									X					How much does aortic aneurysm affect your social activities? (E.g. Visiting friends or family)
AAA		ADL	4				X					X					Do you suffer from low mood because of having an aortic aneurysm?
LL		Ischaemic Pain	4	+/-	+/-				X	X	X			X		X	Do you experience any cramping pain in your legs or feet?
LL		Ischaemic Pain	4		+/-				X		X					X	Do you experience cramping pain in your legs or feet when walking?
LL		Ischaemic Pain	4		+/-						X					X	How far can you walk before you experience any cramping pain in your legs or feet?

LL		Ischaemic Pain	4		+/-						X					X	Do you walk more slowly than you would choose to in order to avoid cramping pain in your legs and feet?
LL		Ischaemic Pain	4		-						X					X	Do you experience cramping pain in your legs or feet when walking uphill?
LL		Ischaemic Pain	4		-						X					X	Do you experience pain in your legs or feet when you climb stairs?
LL		Ischaemic Pain	4		-				X	X	X			X	X	X	Do you experience pain in your feet at night?
LL		Ischaemic Pain	4		-												Do you dangle one or both of your legs over the side of the bed to help reduce foot pain?
LL		Ischaemic Pain	4		-				X	X	X			X	X	X	Do you experience severe pain in your legs or feet when you are resting or sitting?
LL		Ischaemic Pain	2		-											X	Are you troubled by cold feet?
LL		Ulcer	4	X	-					X	X			X	X	X	Have you ever had any ulcers on your legs or feet now or at any time in the past?
LL		Ulcer	4		-					X	X			X	X	X	Please use the image below to show where you currently have any leg or foot ulcers.
LL		Ulcer	4	-	-					X					X	X	Are you concerned about the smell of your leg ulcers?
LL		Ulcer	4	X	-										X	X	Are you concerned about the appearance of your leg ulcers?
LL		Ulcer	4	-	-										X	X	Do you have leg ulcers that leak fluid (watery liquid)?
LL		Ulcer	4	-	-										X	X	Do you experience infections in your leg ulcers? (E.g. foul smell or pus)
LL		Ulcer	4	-	-					X					X	X	Do you experience repeated leg ulcers?
LL		Ulcer	4	X	-					X					X	X	Do you worry about your leg ulcers? (E.g. Not healing becoming infected losing part of your leg or foot).
LL		VV	4		-	-								X			Do you experience any bleeding from veins in your legs or feet?
LL		VV	4		-	X			X					X			Do you have any problems with the skin over your varicose veins?
LL		VV	4		-	X			X					X			Do varicose veins make you feel self-conscious or embarrassed?
LL		VV	4	X	-	X			X					X	X	X	Do leg or foot problems affect what clothing or shoes you can wear?
LL		VV	4		-	X			X					X	X		Do you experience any swelling in your legs or feet?
LL		VV	4		-	X			X					X	X	X	Do you experience itching in your legs or feet?
LL		VV	4		-	X			X					X	X		Do you wear compression stockings or tights for your legs?
LL		Tissue loss	4		-	-				X	X					X	Have you lost any part of your legs or feet through amputation or gangrene?
LL		Tissue loss	4			-				X	X					X	Please click on the appropriate part or parts of your legs feet or toes that you have had amputated or have been lost â€¦
LL		Anxiety	3	X	X	X			X	X	X			X	X	X	Do you worry about your leg problems getting worse in the future?

**LL**		**ADL**	**3**	+/-	**X**	+/-			**X**	**X**	**X**			**X**	**X**	**X**	**How much do leg or foot problems affect your overall enjoyment of life?**
**LL**		**ADL**	**3**	+/-	**X**	-			**X**	**X**	**X**			**X**	**X**	**X**	**How much do leg or foot problems affect your ability to carry out physical activities? (E.g. Walking housework or exercise)**
**LL**		**ADL**	**3**	+/-	**X**	+/-			**X**	**X**	**X**			**X**	**X**	**X**	**How much do leg or foot problems affect your personal responsibilities? E.g. caring for others study or work**
**LL**		**ADL**	**3**	-	**X**	+/-			**X**	**X**	**X**			**X**	**X**	**X**	**How much do leg or foot problems affect your ability to look after yourself? (E.g. Rest wash toilet or feed yourself)**
**LL**		**ADL**	**3**	+/-	**X**	+/-			**X**	**X**	**X**			**X**	**X**	**X**	**How much do leg or foot problems affect your social activities? (E.g. Going out visiting friends or family)**
**LL**		**ADL**	**4**	**X**	**X**	-			**X**	**X**	**X**			**X**	**X**	**X**	**Do you suffer from low mood because of leg or foot problems?**
**Generic**		**Personal data**		-	**X**	-	**X**	**X**		**X**	**X**	**X**	**X**		**X**	**X**	**Do you rely on any people to help you with your everyday activities?**
**Generic**		**Personal data**		-			**X**		**X**	**X**	**X**	**X**			**X**	**X**	**Do you experience financial problems because of your vascular condition?**

Abbreviations: Abdominal aortic aneurysm (AAA), Aberdeen varicose veins questionnaire (AVVQ), Activity of daily living (ADL), Carotid artery disease (CAD), Peripheral arterial disease (PAD), , Lower limb (LL), Peripheral arterial disease (PADQOL), Venous leg ulcer (VLU), Venous leg ulcer Quality of life (VLU-QOL), Varicose veins (VV). The items of the eight scales are colored in this table.

Table 6: Structure of the main ePAQ-VAS with evidence base for inclusion of individual items, scales and sections.

Figure 2. Evidence synthesis to develop of ePAQ-VAS

Insert figure 2 here

ePAQ-VAS was presented to 19 vascular patients. Overall the response was positive; the participants felt the generic and the relevant disease specific were comprehensive, fit for purposeand potentially useful. There was little consistency in items that participants found difficult and no individual item was identified with which most participants had difficulty.

Discussion included the use of abbreviations, font size and contrast between text and background, response options and scales, electronic format versus paper format, relevance to patients and clinicians, the use of free text boxes and the language and wording used, when and how to use the skip button, repetition of items and subject matter and the possibility of emotional distress associated with questions about the possibility of deterioration or death.

For peer review only - http://bmjopen.bmj.com/site/about/guidelines.xhtml

Figure 3. Overview of ePAQ-VAS structure

Insert figure 3 here

Based on the findings from the face validity exercise, and input from the Vascular PROMs group, further revisions were made in an iterative process; culminating in the development of ePAQ-VAS. The structure of the questionnaire is illustrated in Figure 3. Fifty-nine items were eliminated for overlap; these include questions asking about common symptoms experienced across most vascular conditions. Five items were added based on suggestion from clinicians. Generic items for all respondents were presented in the first section and include questions about pain, altered sensation, weakness, weight/height, smoking habit, previous medical history and regular medication. This information was deemed important for assessment of vascular conditions both by patients and clinicians.

The next three sections are condition specific relating to CAD, AAA and LL vascular disease sections. These sections are further divided into scales. There are fifty-five items within eight scales and the remainder of questions do not contribute to scales but are kept due to their clinical relevance. The eight scales are part of the of condition specific sections and include CAD related anxiety, impact of CAD on activities of daily living (ADL), AAA related anxiety, Impact of AAA on activities of ADL, PAD symptoms, VLU symptoms, VV symptoms and Impact of LL vascular disease on ADL. Individual items, scales and sections of ePAQ-VAS in its initial version can be viewed on https://demo-questionnaire.epaq.co.uk/home/project?id=VASC_1.6&page=1

The evidence used to develop each item in ePAQ-VAS is made explicit in **Table 5**, this table show whether the source for the item is the qualitative study, reviews or consensus study.

For peer review only

Discussion

This study documents stages undertaken to develop ePAQ-VAS and the conceptual framework underpinning this new tool for use in undifferentiated vascular populations. The main strength of this new instrument is that it can be used as a holistic clinical assessment tool that can be completed by patients before meeting the vascular surgeon in the clinic. The information generated can be used to help shared decision making by focusing on patient priorities. This tool has the further advantage of being an electronic online patient reported outcome measure since it can be used to monitor impact of the disease and/or interventions overtime. Furthermore, this instrument is preference based, unlike the identified vascular PROMs **(4-8)**; once further validated the disease specific scales can be used to generate utility values either by mapping to the values of a generic utility measure, or by further utility studies **(18).**

This instrument has been developed in line with FDA guidelines for developing PROMs **(3)**. Items were developed based on themes extracted from primary qualitative data, systematic qualitative evidence synthesis and clinicians' consensus exercise **(9-13)**. We have made efforts through purposive sampling to ensure that we have included diverse demographic groupings in the primary research, and this is augmented by the inclusion of systematic reviews that include evidence gathered in national and international studies. Another strength of this study is that the qualitative evidence in the review and the primary study included patients at different stages of their disease. The data collected included the impact of disease, including symptoms, on daily living and the impact of diagnosis and treatment on the daily living. The vascular clinicians' input into developing and rating the items was sought, and new items were incorporated based on recommendation from twenty-five vascular clinicians.

The work of developing individual items and their assignment to putative scales and sections was based both on the framework of existing PROMs **(4-8),** and on input from vascular clinicians. In this stage of the ePAQ-VAS development, an inclusive approach was chosen, and all relevant items were incorporated except for those with clear repetition. The main limitation of this draft version of ePAQ-VAS is that it is long and potentially repetitive; It is expected that factor analysis and psychometric

For peer review only

testing will lead to a reduction in the number of individual items and will confirm (or refute) the putative scales identified in the current version. Furthermore, skipping rules embedded within the questionnaire will only present the items relevant to the patient completing the online instrument.

Another limitation is that ePAQ-VAS only cover the five main vascular conditions and it might not be relevant to patients with other vascular disease. However, including all vascular conditions in one instrument is not possible and the evidence to include only these conditions was based on input from clinicians treating vascular disease. As stated by the FDA **(3)** a fundamental consideration in the development of PROMs is the adequacy of item generation. Due to the heterogeneous nature of vascular disease, it was not straightforward to identify what exactly should be measured when developing and defining the initial conceptual framework for the ePAQ-VAS. To this end, as recommended by the FDA, the initial conceptual framework was based on information gathered from reviews of the literature, patients and expert opinion.

The findings of the qualitative study indicated an overlap in patient experiences of the various conditions. However, there was also a clear difference in how each condition impacted on different aspects of quality of life. There were conditions with many physical symptoms and others with none. This demonstrated that while it may be possible to develop a PROMs for use across a variety of vascular conditions, it would also need to include condition-specific items to fully capture the impact and clinically relevant information for each disease or condition. A further limitation is that the face validity study was not able to examine the comprehensiveness of ePAQ-VAS since it covered multiple conditions and it was difficult to expect from any of the patient groups interviewed to comment on diseases they have not experienced. Therefore, they only commented on the generic questions and the disease specific items relating to their condition.

In conclusion ePAQ-VAS is a multi-dimensional measure developed for use in a range of vascular conditions. It is a single electronic tool, covering most vascular conditions. This is important for those patients presenting with mixed symptoms or multiple conditions. The items in ePAQ-VAS can capture information about disease

For peer review only

symptoms, HRQoL, co-morbidities, medical history and other relevant healthcare issues. This type of information can aid communication between healthcare professionals and patients and support shared-decision making. The electronic format may make it easier to monitor patients over time, especially those with chronic conditions and those treated with lifestyle modification or conservatively. Based on methods used in its construction, this tool has a strong degree of content validity; however, further psychometric testing for reliability, responsiveness, and validity is needed. Once this electronic PROMs is validated, it can be used as an outcome measure in clinical practice and research.

Contributorship statement

JAM, GJ, AA, SCR designed the study. AA, PP, EL, SCR, GJ performed the analysis of the data and theme generation. ST and SN helped with consensus study and qualitative study recruitment. AA wrote the paper and all authors reviewed and edited the paper. All authors approved the manuscript.

Competing interests

Stephen Radley is a shareholder in ePAQ Systems Ltd, an NHS spin-out technology company, the majority shareholder being Sheffield Teaching Hospitals. The other authors have no conflict of interest.

Funding

This manuscript presents independent research funded by the National Institute for Health Research (NIHR) under the Programme Grants for Applied Research (RP-PG-1210-12009).

Data sharing statement

No data are available.

References:

1- Beebe-Dimmer JL, Pfeifer JR, Engle JS, Schottenfeld D. The epidemiology of chronic venous insufficiency and varicose veins. Ann Epidemiol. 2005 Mar;15(3):175-84.
2- Peach G, Griffin M, Jones KG, Thompson MM, Hinchliffe RJ. Diagnosis and management of peripheral arterial disease. BMJ. 2012 Aug 14;345:e5208.
3- Food and Drug Administration (FDA), Guidance for Industry: Patient-Reported Outcome Measures: Use in Medical Product Development to Support Labeling Claims, accessed and available at: https://www.fda.gov/downloads/drugs/guidances/ucm193282.pdf

4- Aber A, Poku E, Phillips P, Essat M, Buckley Woods H, Palfreyman S, Kaltenthaler E, Jones G, Michaels J. Systematic review of patient-reported outcome measures in patients with varicose veins. Br J Surg. 2017 Oct;104(11):1424-1432.

5- Duncan R, Essat M, Jones G, Booth A, Buckley Woods H, Poku E, Kaltenthaler E, Keetharuth AD, Palfreyman S, Michaels J. Systematic review and qualitative evidence synthesis of patient-reported outcome measures for abdominal aortic aneurysm. Br J Surg. 2017 Mar;104(4):317-327. doi: 10.1002/bjs.

6- Poku E, Duncan R, Keetharuth A, Essat M, Phillips P, Woods HB, Palfreyman S, Jones G, Kaltenthaler E, Michaels J. Patient-reported outcome measures in patients with peripheral arterial disease: a systematic review of psychometric properties. Health Qual Life Outcomes. 2016 Nov 24;14(1):161.

7- Poku E, Aber A, Phillips P, Essat M, Buckley Woods H, Palfreyman S, Kaltenthaler E, Jones G, Michaels J. Systematic review assessing the measurement properties of patient-reported outcomes for venous leg ulcers. 2017 Oct;1(5):138-147.

8- Essat M, Aber A, Phillips P, Poku E, Woods HB, Howard A, Palfreyman S, Kaltenthaler E, Jones G, Michaels J. Patient-Reported Outcome Measures in Carotid Artery Revascularization: Systematic Review and Psychometric Analysis. Ann Vasc Surg. 2018 Jul;50:275-283.

9- Aber A, Lumley E, Phillips P, Woods HB, Jones G, Michaels J. Themes that Determine Quality of Life in Patients with Peripheral Arterial Disease: A Systematic Review. Patient. 2018 May 8. doi: 10.1007/s40271-018-0307-7.

10- Phillips P, Lumley E, Duncan R, Aber A, Woods HB, Jones GL, Michaels J. A systematic review of qualitative research into people's experiences of living with venous leg ulcers. J Adv Nurs. 2018 Mar;74(3):550-563.

11- Aber A, Howard A, Woods HB, Jones G, Michaels J. Impact of Carotid Artery Stenosis on Quality of Life: A Systematic Review. Patient. 2018 Oct 17. doi: 10.1007/s40271-018-0337-1.

12- Lumley E, Phillips P, Aber A, Woods HB, Jones G, Michaels J. Experiences of living with varicose veins: a systematic review of qualitative research. J Clin Nurs. 2018 Nov. J Clin Nurs. 2018 Nov 21. doi: 10.1111/jocn.14720.

13- Duncan R, Lumley E, Tod A, Hughes J, Palfreyman S, Nawaz S, Jones G, Michaels J. The experiences of people living with a vascular condition: a qualitative study. HEDS Discussion Papers, University of Sheffield. 2018 Jul. https://www.sheffield.ac.uk/scharr/sections/heds/discussion-papers/18_07-1.794000 (Accessed 19 November 2018).

14- Mokkink LB, Terwee CB, Patrick DL, Alonso J, Stratford PW, Knol DL et al. The COSMIN study reached international consensus on taxonomy, terminology, and definitions of measurement properties for health-related patient-reported outcomes. J Clin Epidemiol 2010; 63: 737–745.

15- Ritchie J, Spencer L. Qualitative data analysis for applied policy research. In: Bryman G, Burgess A, editors. Analysing qualitative data. London: Routledge; 1994. p. 173–94.

16- Hsu, C., & Sandford, B. (2007). The Delphi Technique : Making Sense Of Consensus. Practical Assessment, Research & Evaluation, 12(10). http://www.endoedu.com/mobile/guideline/delphi.pdf (Accessed 19 November 2018).

17- Garratt AM, Macdonald LM, Ruta DA, Russell IT, Buckingham JK, Krukowski ZH. Towards measurement of outcome for patients with varicose veins. Qual Health Care 1993; 2: 5–10

18- Brazier J, Deverill M. A checklist for judging preference-based measures of health related quality of life: learning from psychometrics. Health Econ 1999; 8: 41–5

For peer review only

For peer review only

For peer review only

Figure 1. Development of ePAQ-VAS conceptual framework

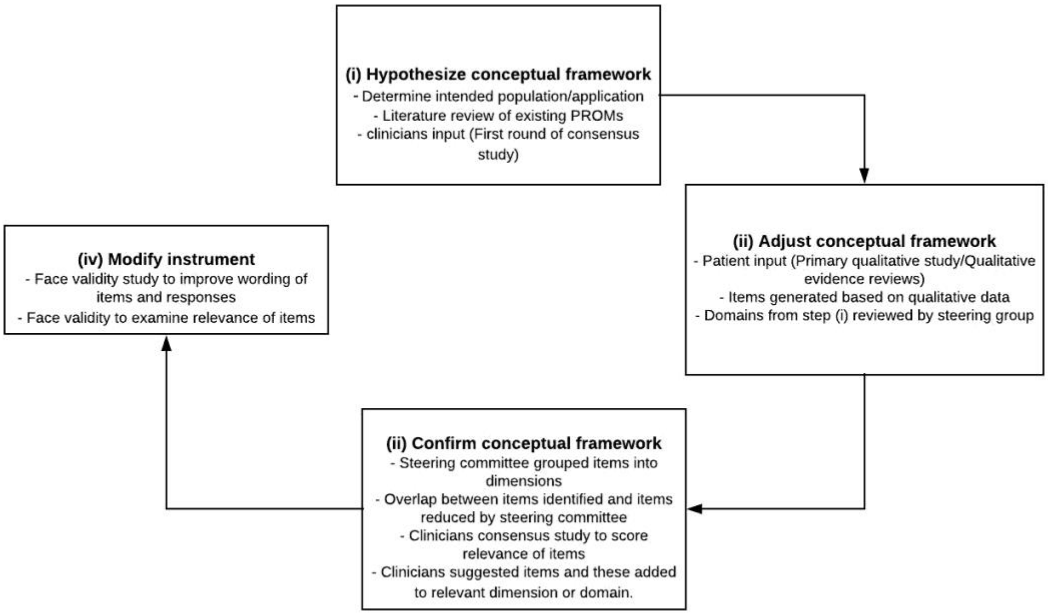

For peer review only

Figure 2. Evidence synthesis to develop of ePAQ-VAS

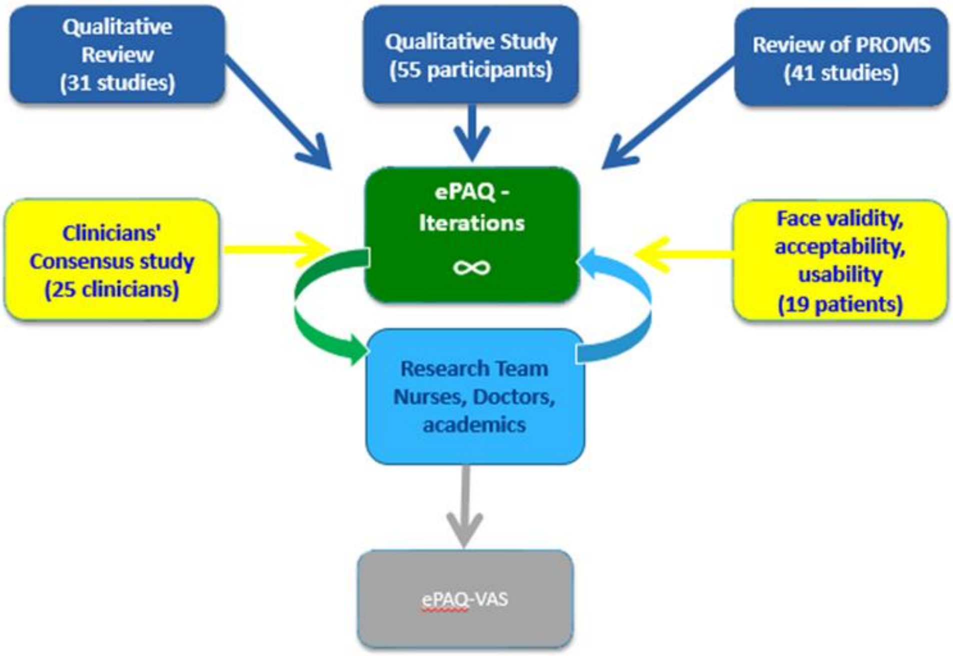

For peer review only

Figure 3. Overview of ePAQ-VAS structure

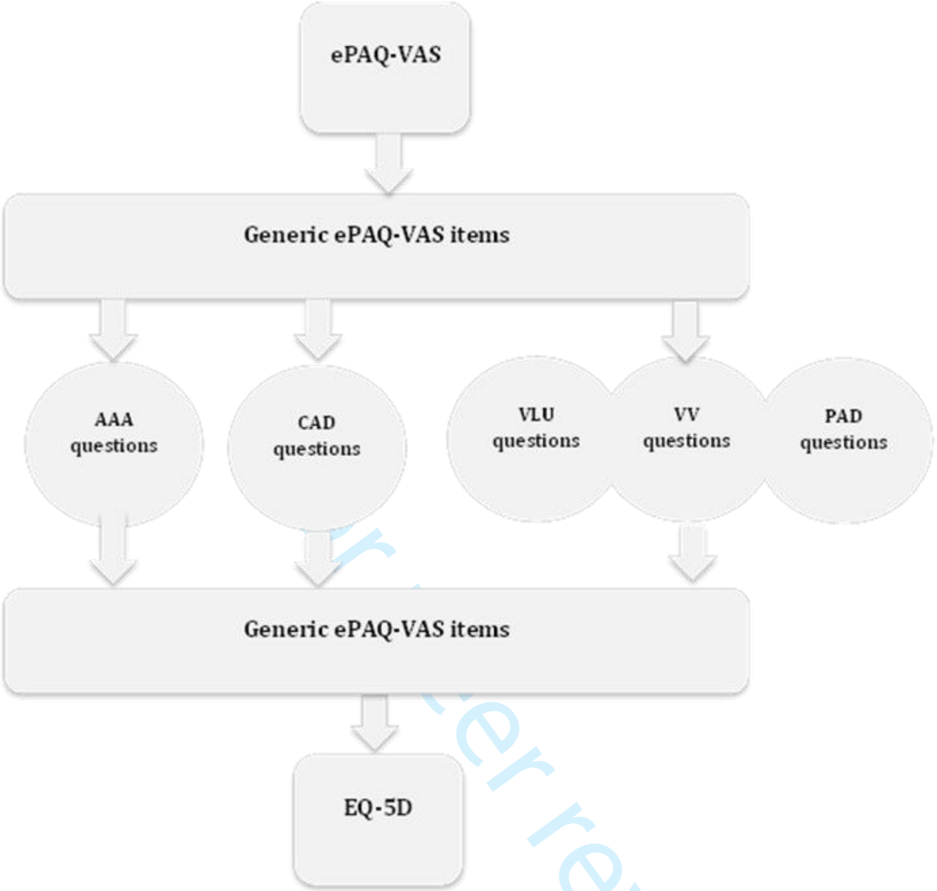

Supplementary material

S 1

Table 1- Appraisal criteria for assessing the psychometric properties of patient-reported outcome measures

Psychometric property domains	Subdomain	Thresholds
**Reliability**	**Test–retest reliability**	**Intraclass correlation/weighted κ ≥ 0·70 for group comparisons**
		**Intraclass correlation/weighted κ ≥ 0·90 for individual scores**
		**Evidence of mean difference between time point 1 and time point 2, reported with 95 per cent c.i. (using paired *t* test or Wilcoxon signed-rank test)**
	**Internal consistency**	**Cronbach's α score of ≥ 0·70 indicates good evidence, but the score should not exceed ≥ 0·92 for group comparison**
		**Item total correlations should be ≥ 0·20**
**Validity**	**Content validity**	**Evidence that the instrument has been developed by undertaking a literature review, consulting patients, clinicians and other experts**

For peer review only

Psychometric property domains	Subdomain	Thresholds
	Construct validity	Correlation coefficient of ≥ 0·60 indicates strong evidence<br><br>This should be supported by specific directional hypotheses and a previous estimation of strength of correlation.
	Criterion validity	Justification for selection of standard should be adequate<br><br>Correlation between PROM and standard ≥ 0·70
Responsiveness	Responsiveness	Statistically significant changes in score of an expected magnitude based on methods including *t* tests, effect size, standardized response means, Guyatt's responsiveness index or responsiveness statistics
Acceptability	Floor/ceiling effects	Evidence of floor effect: 15 per cent of respondents are achieving the lowest score on the instrument<br><br>Evidence of ceiling effect: 15 per cent of respondents are achieving the highest score on the instrument
	Acceptability	Completeness of data supplied ≥ 80 per cent

For peer review only

S 2

AAA search strategies: search one
Database: Ovid MEDLINE(R) In-Process & Other Non-Indexed Citations and Ovid MEDLINE(R) <1946 to Present>
Search Strategy:
----
1. (Aortic aneurysm$ or triple A or true aneurysm$).tw.
2. Aortic Aneurysm/
3. Aortic Aneurysm, Abdominal/
4. 1 or 2 or 3
5. (patient reported outcome$ or patient-reported outcome$).tw.
6. (prom or proms).tw.
7. (disease reported outcome$ or disease-reported outcome$).tw.
8. 5 or 6 or 7
9. "Quality of Life"/
10. "Outcome and Process Assessment (Health Care)"/
11. "Outcome Assessment (Health Care)"/
12. quality of life.tw.
13. qol.tw.
14. outcome measure$.tw.
15. health outcome$.tw.
16. or/9-15
17. (patient adj20 report$).tw.
18. 16 and 17
19. 8 or 18
20. nottingham health profile.tw.
21. health related quality of life.tw.
22. health related qol.tw.
23. health related ql.tw.
24. hrqol.tw.
25. hql.tw.
26. health state utilit$.tw.
27. hsuv$.tw.

For peer review only

28. (euroqol or euro qol or eq5d or eq 5d).tw.
29. (sf6d or sf 6d or sf 6 dimension$ or sf six dimension$ or shortform 6d or shortform six dimension$ or short form 6d or short form 6 dimension$ or short form six dimension$).tw.
30. (sf12 or sf 12 or short form 12 or shortform 12 or sf twelve or sftwelve or shortform twelve or short form twelve).tw.
31. (sf36 or sf 36 or short form 36 or shortform 36 or sf thirtysix or sf thirty six or shortform thirtysix or shortform thirty six or short form thirtysix or short form thirty six).tw.
32. (item adj3 short form).tw.
33. (item adj3 shortform).tw.
34. medical outcomes survey.tw.
35. medical outcomes study.tw.
36. mos.tw.
37. psychological general wellbeing index.tw.
38. psychological general well being index.tw.
39. pgwb$.tw.
40. health utilit$.tw.
41. hui$.tw.
42. quality of wellbeing.tw.
43. quality of well being.tw.
44. qwb$.tw.
45. rosser.tw.
46. trade off$.tw.
47. standard gamble$.tw.
48. tto$.tw.
49. qaly$.tw.
50. quality adjusted life year$.tw.
51. quality-adjusted life years/
52. hye$.tw.
53. health$ year$ equivalent$.tw.
54. disutilit$.tw.
55. disbenefit$.tw.
56. "Quality of Life"/
57. "Outcome Assessment (Health Care)"/
58. quality of life.tw.
59. 56 or 57 or 58
60. (preference based or utilit$ or generic preference).tw.
61. 59 and 60
62. (preference$ adj2 (elicit$ or patient$ or population$ or measure$ or based or cost$)).tw.
63. (utilit$ adj2 (elicit$ or patient$ or population$ or measure$ or based or cost$)).tw.
64. or/20-55

For peer review only

65. 61 or 62 or 63 or 64
66. 19 or 65
67. 4 and 66

AAA search strategies: search two
Database: Ovid MEDLINE(R) In-Process & Other Non-Indexed Citations and Ovid MEDLINE(R) <1946 to Present>
Search Strategy:
----
1. (Aortic aneurysm$ or triple A or true aneurysm$).tw.
2. Aortic Aneurysm/
3. Aortic Aneurysm, Abdominal/
4. or/1-3
5. time trade off.mp.
6. Watt index.mp.
7. (Rand-36 or Rand36 or Rand 36).mp.
8. self-assessed health.mp.
9. screenQL.mp.
10. general health questionnaire.mp.
11. female sexual function index.mp.
12. Rose questionnaire.mp.
13. Australian Vascular Quality of Life Index.mp.
14. AUSVIQUOL.mp.
15. Nottingham Health Profile.mp.
16. NHP.mp.
17. (sf36 or sf 36 or short form 36 or shortform 36 or sf thirtysix or sf thirty six or shortform thirtysix or shortform thirty six or short form thirtysix or short form thirty six).mp. [mp=title, abstract, original title, name of substance word, subject heading word, keyword heading word, protocol supplementary concept word, rare disease supplementary concept word, unique identifier]
18. (sf12 or sf 12 or short form 12 or shortform 12 or sf twelve or sftwelve or shortform twelve or short form twelve).mp. [mp=title, abstract, original title, name of substance word, subject heading word, keyword heading word, protocol supplementary concept word, rare disease supplementary concept word, unique identifier]
19. (sf20 or sf 20 or short form 20 or shortform 20 or sf twenty or sftwenty or shortform twenty or short form twenty).mp. [mp=title, abstract, original title, name of substance word, subject heading word, keyword heading word, protocol supplementary concept word, rare disease supplementary concept word, unique identifier]
20. (Hospital Anxiety and Depression Scale).mp. [mp=title, abstract, original title, name of substance word, subject heading word, keyword heading word, protocol supplementary concept word, rare disease supplementary concept word, unique identifier]
21. HADS.mp.
22. Standard gamble$.mp. [mp=title, abstract, original title, name of substance word, subject heading word, keyword heading word, protocol supplementary concept word, rare disease supplementary concept word, unique identifier]
23. rosser.tw.
24. (euroqol or euro qol or eq5d or eq 5d or eq-5d or eq-5d VAS).mp. [mp=title, abstract, original title, name of substance word, subject heading word, keyword heading word, protocol supplementary concept word, rare disease supplementary concept word, unique identifier]

For peer review only

25. World Health Organization Quality of Life-BREF.mp. [mp=title, abstract, original title, name of substance word, subject heading word, keyword heading word, protocol supplementary concept word, rare disease supplementary concept word, unique identifier]
26. WHOQOL-BREF.mp. [mp=title, abstract, original title, name of substance word, subject heading word, keyword heading word, protocol supplementary concept word, rare disease supplementary concept word, unique identifier]
27. or/5-26
28. instrumentation.sh.
29. methods.sh.
30. Validation Studies.pt.
31. Comparative Study.pt.
32. Psychometrics/
33. psychometr*.ti,ab.
34. clinimetr*.tw.
35. clinometr*.tw.
36. "Outcome Assessment (Health Care)"/
37. outcome assessment.ti,ab.
38. outcome measure*.tw.
39. Observer Variation/
40. observer variation.ti,ab.
41. Health Status Indicators/
42. "Reproducibility of Results"/
43. reproducib*.ti,ab.
44. Discriminant Analysis/
45. reliab*.ti,ab.
46. unreliab*.ti,ab.
47. valid*.ti,ab.
48. coefficient.ti,ab.
49. homogeneity.ti,ab.
50. homogeneous.ti,ab.
51. "internal consistency".ti,ab.
52. 28 or 29 or 30 or 31 or 32 or 33 or 34 or 35 or 36 or 37 or 38 or 39 or 40 or 41 or 42 or 43 or 44 or 45 or 46 or 47 or 48 or 49 or 50 or 51
53. cronbach*.ti,ab.
54. (alpha or alphas).ti,ab.
55. 53 and 54
56. item.ti,ab.
57. (correlation* or selection* or reduction*).ti,ab.
58. 56 and 57
59. (agreement or precision or imprecision or "precise values" or "test–retest").ti,ab.
60. (test and retest).ti,ab.
61. reliab*.ti,ab.

For peer review only

62. (test or retest).ti,ab.
63. 61 and 62
64. 55 or 58 or 59 or 60 or 63
65. (stability or interrater or inter-rater or intrarater or intra-rater or intertester or inter-tester or intratester or intra-tester or interobserver or inter-observer or intraobserver or intra-observer).ti,ab.
66. (intertechnician or inter-technician or intratechnician or intra-technician or interexaminer or inter-examiner or intraexaminer or intra-examiner or interassay or inter-assay or intraassay or intra-assay or interindividual or inter-individual or intraindividual or intra-individual or interparticipant or inter-participant or intraparticipant or intra-participant).ti,ab.
67. (kappa or "kappa's" or kappas or repeatab*).ti,ab.
68. repeatab*.ti,ab.
69. 65 or 66 or 67 or 68
70. (replicab* or repeated).ti,ab.
71. (measure or measures or findings or result or results or test or tests).ti,ab.
72. 70 and 71
73. (generaliza* or generalisa* or concordance).ti,ab.
74. (intraclass and correlation*).ti,ab.
75. (discriminative or "known group" or factor analysis or factor analyses or dimension* or subscale*).ti,ab.
76. (multitrait and scaling and (analysis or analyses)).ti,ab.
77. 72 or 73 or 74 or 75 or 76
78. (item discriminant or interscale correlation* or error or errors or "individual variability").ti,ab.
79. (variability and (analysis or values)).ti,ab.
80. (uncertainty and (measurement or measuring)).ti,ab.
81. ("standard error of measurement" or sensitiv* or responsive*).ti,ab.
82. ((minimal or minimally or clinical or clinically) and (important or significant or detectable) and (change or difference)).ti,ab.
83. (small* and (real or detectable) and (change or difference)).ti,ab.
84. (meaningful change or "ceiling effect" or "floor effect" or "Item response model" or IRT or Rasch or "Differential item functioning" or DIF or "computer adaptive testing" or "item bank" or "cross-cultural equivalence").ti,ab.
85. 78 or 79 or 80 or 81 or 82 or 83 or 84
86. 52 or 64 or 69 or 77 or 85
87. 4 and 27 and 86

Databases and research registers searched:

- MEDLINE and MEDLINE In Process: Ovid.
- EMBASE: Ovid.
- Cochrane Library
  - Cochrane Database of Systematic Reviews (CDSR)
  - Database of Abstracts of Reviews of Effects (DARE)
  - Cochrane Central Register of Controlled Trials (CCRCT)

For peer review only

   - Health Technology Assessment Database (HTA)
   - NHS Economic Evaluation Database (NHS EED)
- CINAHL
- PROQOLID
- PsychINFO
- Science Citation Index Expanded (SCIE): Web of Science

Varicose veins systematic review search strategy

1 ((Venous or vein) adj2 (disease or stasis or obstruction or incompetence or insufficiency or syndrome or thromboembolism)).tw. (25175)
2 (Venous ulcer$ or leg ulcer$ or venous leg ulcer$).tw. (6632)
3 (Venous insufficiency or Venous incompetence or Venous thromboembolism or VTE or Venous outflow obstruction or VOO or Venous valvular incompetence or VVI).tw. (19687)
4 exp Venous Insufficiency/ (6167)
5 exp Varicose Veins/ (15946)
6 (Venous adj2 (edema or oedema)).tw. (262)
7 Venous disorder$ of the leg$.tw. (16)
8 (Varicose vein$ or spider vein$ or varicosity or varices or telangiectasia$).tw. (28086)
9 Endovenous occlusion.tw. (11)
10 post-thrombotic syndrome.tw. (716)
11 Venous Thrombosis/ or Venous Thromboembolism/ (24327)
12 or/1-11 (82600)
13 (patient reported outcome$ or patient-reported outcome$).tw. (5201)
14 (prom or proms).tw. (1755)
15 (disease reported outcome$ or disease-reported outcome$).tw. (2)
16 13 or 14 or 15 (6667)
17 "Quality of Life"/ (129051)
18 "Outcome and Process Assessment (Health Care)"/ (22708)
19 "Outcome Assessment (Health Care)"/ (52618)
20 quality of life.tw. (169753)
21 qol.tw. (22367)
22 outcome measure$.tw. (161039)
23 health outcome$.tw. (26145)
24 ((evalua$ or assess$ or measure$) adj (function$ or symptom$)).tw. (8832)
25 or/17-24 (451890)
26 (patient adj20 report$).tw. (188387)
27 25 and 26 (14509)
28 16 or 27 (17478)

For peer review only

29 (nottingham health profile or aberdeen varicose vein questionnaire or AVVQ or chronic venous insufficiency questionnaire or CIVIQ or charing cross venous ulceration questionnaire or CXVUQ or Venous Insufficiency Epidemiological Economic Study tool or VEINES-QOL or VEINES-SYM or Venous Clinical Severity Score or VCSS or Deep Vein Thrombosis Leg Symptom Index or DVT-LSI or Ferrans Powers Quality of Life Index or QLI).tw. (1447)
30 health related quality of life.tw. (25867)
31 health related qol.tw. (978)
32 health related ql.tw. (5)
33 hrqol.tw. (8922)
34 hql.tw. (87)
35 health state utilit$.tw. (334)
36 hsuv$.tw. (35)
37 (activities of daily living or ADL).tw. (19652)
38 "Activities of Daily Living"/ (52765)
39 (euroqol or euro qol or eq5d or eq 5d).tw. (5064)
40 (sf8 or sf 8 or short form 8 or shortform 8 or sf eight or shortform eight or short form eight).tw. (310)
41 (sf6d or sf 6d or sf 6 dimension$ or sf six dimension$ or shortform 6d or shortform six dimension$ or short form 6d or short form 6 dimension$ or short form six dimension$).tw. (524)
42 (sf12 or sf 12 or short form 12 or shortform 12 or sf twelve or sftwelve or shortform twelve or short form twelve).tw. (3332)
43 (sf36 or sf 36 or short form 36 or shortform 36 or sf thirtysix or sf thirty six or shortform thirtysix or shortform thirty six or short form thirtysix or short form thirty six).tw. (18047)
44 (item adj3 short form).tw. (3935)
45 (item adj3 shortform).tw. (1)
46 medical outcomes survey.tw. (240)
47 medical outcomes study.tw. (3459)
48 mos.tw. (5442)
49 psychological general wellbeing index.tw. (10)
50 psychological general well being index.tw. (203)
51 pgwb$.tw. (252)
52 health utilit$.tw. (1278)
53 hui$.tw. (2939)
54 quality of wellbeing.tw. (8)
55 quality of well being.tw. (354)
56 qwb$.tw. (221)
57 rosser.tw. (74)
58 trade off$.tw. (12716)
59 standard gamble$.tw. (732)
60 tto$.tw. (1143)
61 qaly$.tw. (6062)
62 quality adjusted life year$.tw. (7209)
63 quality-adjusted life years/ (7816)

For peer review only

64 hye$.tw. (753)
65 health$ year$ equivalent$.tw. (39)
66 disutilit$.tw. (274)
67 disbenefit$.tw. (24)
68 "Quality of Life"/ (129051)
69 "Outcome Assessment (Health Care)"/ (52618)
70 quality of life.tw. (169753)
71 68 or 69 or 70 (262013)
72 (preference based or utilit$ or generic preference).tw. (136252)
73 71 and 72 (7974)
74 (preference$ adj2 (elicit$ or patient$ or population$ or measure$ or based or cost$)).tw. (11249)
75 (utilit$ adj2 (elicit$ or patient$ or population$ or measure$ or based or cost$)).tw. (5889)
76 or/29-67 (136441)
77 73 or 74 or 75 or 76 (150495)
78 28 or 77 (164078)
79 12 and 78 (993)

S3

AAA qualitative literature review
Database: Ovid MEDLINE(R) In-Process & Other Non-Indexed Citations and Ovid MEDLINE(R) <1946 to Present>
Search Strategy:
----
1 (Aortic aneurysm$ or triple A or true aneurysm$).tw.
2 Aortic Aneurysm/
3 Aortic Aneurysm, Abdominal/
4 or/1-3 (38292)
5 *Attitude to Health/
6 *Self Care/
7 *Health Knowledge, Attitudes, Practice/
8 (patient* adj4 (feeling* or emotion* or view* or symptom* or perception* or attribute*)).ti,ab.
9 ("health related quality of life" or "health related qol" or "health related ql" or hrqol or hql or "patient reported outcome*" or "patient-reported outcome*" or prom or proms or "disease reported outcome*").ti,ab.
10 ("quality of life" or "qol" or "outcome measure*" or "health outcome*").ti,ab.
11 *"Quality of Life"/
12 *"Outcome Assessment (Health Care)"/ or *"Outcome and Process Assessment (Health Care)"/
13 5 or 6 or 7 or 8 or 9 or 10 or 11 or 12
14 (qualitative* or findings or interview*).mp.

For peer review only

15 focus groups/ or interviews as topic/
16 exp qualitative research/
17 14 or 15 or 16
18 14 or 15 or 16
19 4 and 13
20 17 and 19

****

Databases and research registers which were searched:

- MEDLINE and MEDLINE In Process: Ovid.
- EMBASE: Ovid.
- CINAHL: EBSCO
- PROQOLID
- PsychINFO
- Science Citation Index Expanded (SCIE): Web of Science
- Social Science Citation Index
- Proquest dissertation and theses

For peer review only

S4

Table 1: Example of triangulation between PAD PROM review, qualitative evidence synthesis and qualitative interview themes

Qualitative Review Themes	Qualitative Interview Themes	EQ-5D	NHP	SF-6D	SF-8	SF-36	AUSVI QoL	ICQ	PADQOL	SIPic	WIQ	VascuQOL
PAD Health Outcomes												
**Pain - location**	Positive	+/-	-	-	-	-	+	-	+	+/-	-	+
**Pain - sensation**	Positive	+/-	+	+	+	+	-	+	+	-	+	-
**Pain - severity**	Positive	+	-	-	-	-	-	-	+/-	+	-	+
**Pain on walking**	Positive	+	+	-	-	-	+	+	+	+	+	+
**Pain at rest**	Positive	+	+	-	-	-	+	+	+	-	-	+
**Mobility - walking ability**	Postive	+/-	+	-	-	+	+	+	+	+	+	+
**Mobility - walking distance**	Positive	+/-	+/-	-	-	+	+	+	-	+	+	+
**Mobility - walking speed**	Positive	+/-	-	-	-	-	-	-	-	-	+	-
**Mobility - stair climbing**	Positive	+/-	+/-	-	-	+	-	+	-	+	+	-

For peer review only

**Mobility - walking uphill**	Positive	+/-	-	-	-	-	-	-	-	-	-	-
**Bending/ picking up things**	Negative	+/-	-	-	-	+	-	-	-	-	-	-
**Sleep**	Positive	-	+	-	-	+	+	-	-	-	-	--
**Sleep/ pain**	Silence	-	+	-	-	-	+	-	-	-	-	-
**Fatigue**	Negative	-	+	-	-	+	-	-	-	-	-	+
**Sexual functioning**	Negative	-	+	-	-	-	-	-	-	-	-	-
**Comorbiditi es**	Negative	-	-	-	-	-	-	-	-	-	-	-
**Non-healing wounds**	Positive	-	-	-	-	-	+	-	-	-	-	-
**Symptom progression**	Postive	-	-	-	-	-	-	-	-	-	-	-
**Appearance of leg**	Silence	-	-	-	-	-	+	-	-	-	-	-
**Altered sensations in leg**	Silence	-	-	-	-	-	-	-	-	-	-	-
**Foot temperature**	Silence	-	-	-	-	-	-	-	-	-	-	-
Impact on Physical Functioning												
**Personal care**	Positive	+	+	+/-	+/-	+	+	-	+	+	-	+
**Hobbies**	Positive	+	-	-	-	+/-	-	+	+	+	-	+/-
**Exercise**	Negative	+/-	-	-	-	+/-	-	-	-	-	-	+/-

For peer review only

**Daily activities**	Positive	+/-	+	+	+	+	+	+	+	+	-	+
Impact on Social Functioning												
**Social support**	Positive	-	+	-	-	-	-	-	-	-	-	-
**Social activities**	Positive	-	+	+	+	+	+	+	+	+	-	+
**Role**	Positive	-	+/-	+	+	-	-	+	+	-	-	+/-
**Isolation**	Positive	-	+	+/-	+/-	-	+	-	+	-	-	-
**Independence/ burden**	Positive	-	+	+	-	+/-	-	+	-	-	-	+
Impact on Psychological Function												
**Loss/ grief**	Positive	-	-	-	-	+/-	+	-	+	-	-	
**Unfulfilled desire**	Negative	-	+	-	-	-	-	-	+	-	-	
**Illness beliefs**	Positive	-	+	-	-	-	-	-	+	-	-	
**Feeling old**	Positive	-	-	-	-	-	-	-	-	-	-	
**Feeling abnormal**	Positive	-	-	-	-	-	-	-	+	-	-	
**Emotions/ mood**	Positive	-	+/-	+	+	+	+	+	+	+	-	
**Depression**	Positive	-	+	+	+	+	+	+	+	-	-	
**Fears**	Positive	-	-	-	-	+	+	+	+	-	-	
**Concentration/ intellectual**		-	-	-	-	-	-	-	+	-	-	

For peer review only

**capacity**												
**Treatment beliefs**		-	-	-	-	-		-	+	-	-	
**Uncertainty over the future**	Partial agreement	-	-	-	-	-	-	+/-	+	-	-	
Key:<br>. = silence<br>-/+ = partial agreement<br>- = dissonance<br>+ = agreement												

For peer review only

S5

AAA - Symptom anxiety	—		
AAA - Size increase anxiety	—		
Rupture	—		
Lifting	—		
AAA - Anxiety Independent travel	—		
AAA - ADL Overall impact	—		
AAA - ADL Physical activities	—		
AAA - ADL Personal responsibilities	—		
AAA - ADL Independent living	—		
AAA - ADL Social activity	—		
AAA - ADL Low mood	—		

ePAQ VASC 1.7 Minnie Mouse Completed on 27/11/2019

Name	Minnie Mouse	Date of birth	21/09/1951	NHS Number	111 222 TEST (02)
Clinician	Mr PAD Surgeon	Clinic	Claudication Clinic	Hospital Number	666555

Lower Limb

Lower limb - Vascular disease	Yes	Lower limb - Vascular disease date	Over 5 years ago
Lower limb - Precise diagnosis date			
Lower limb - Treatments	Yes		
Lower limb - Procedures	Bypass graft of a blockage , Balloon stretch of a vessel ,	LL - Other procedures	—
Lower limb - Cramping pain	0 1 **2** 3	Impact	0 **1** 2 3
Lower limb - Walking	Most of the time		
Lower limb - Walking distance	0 1 2 **3**		
Lower limb - Walking speed	0 1 **2** 3		
Lower limb - Walking uphill	0 1 **2** 3		
Lower limb - Climbing stairs	0 1 **2** 3		
Lower limb - Pain at night	Never	Impact	—
Lower limb - Dangling	—		
Lower limb - pain resting	—		
Lower limb - Leg ulcers	—	Impact	—
Lower limb - Ulceration	Yes	Lower limb - Ulceration first notice	3 - 5 years
LL - Ulceration location	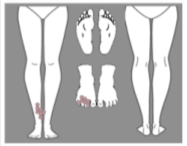		
LL - Ulceration smell	**0** 1 2 3		
LL - Ulceration appearance	**0** 1 2 3		
LL - Ulceration fluid leakage	**0** 1 2 3		
LL - Ulceration infection	**0** 1 2 3		
LL - Ulceration recurrence	No		
LL - Ulceration healing anxiety	**0** 1 2 3		

©ePAQ systems Ltd 2020

ePAQ VASC 1.7 Minnie Mouse Completed on 27/11/2019

Name	Minnie Mouse	Date of birth	21/09/1951	NHS Number	111 222 TEST (02)
Clinician	Mr PAD Surgeon	Clinic	Claudication Clinic	Hospital Number	666555

Carotid Artery Disease

CAD Diagnosis	No	Diagnosis time frame	—
CAD - Diagnosis date			
CAD - Previous TIA	—	CAD - TIA Count	—
CAD - Most recent TIA			
CAD - First TIA			
CAD - Previous Stroke	—	CAD - Stroke Count	—
CAD - Most recent stroke			
CAD - First stroke			
CAD - TIA/Stroke Anxiety	—		
CAD - Anxiety	—		
Health anxiety	—		
Independence anxiety	—		
CAD - Balance	—		
CAD - Memory symptoms	—		
CAD - Altered speech	—		
CAD - Swallowing	—		
CAD - Vision	—		
Right eye symptoms	—		
CAD - Left eye	—		
CAD - ADL overall impact	—		
CAD - Physical activity	—		
CAD - Personal responsibilities	—		
CAD - Independent living	—		
CAD - Social activity	—		

©ePAQ systems Ltd 2020

ePAQ electronic Personal Assessment Questionnaire **Ms Minnie Mouse - 666555 - 111222TEST - 27/11/2019** Page 3 of 7

Frequency of pain	Occasionally	Pain - length of time	Less than 6 months
Pain - ADL Overall impact	0 1 2 3		
Sensation change	Yes	Cause	Numb right below knee stump
Sensation change - Location	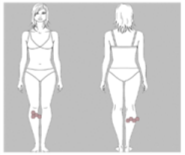		
Sensation change - Description	Stump of right leg has always been numb since my operation		
Sensation change - Frequency	All of the time	Sensation change - Duration	2 or more years
Sensation change - ADL Overall impact	0 1 2 3		
Weakness	No	Weakness - Cause	**##QuestionShown##**
Weakness location	—		
Weakness - Description	—		
Weakness - Frequency	—	Weakness - Duration	—
Weakness - ADL Overall impact	—		
BMI	Height:157.48cm Weight:60.33kg BMI: 24		
Smoking status	Ex-smoker (quit more than 1 year ago)	Current habit	
Smoking history	30		
Smoking history cont.	15		
Smoking habit	;		
Diabetes status	Yes	Diabetes duration	3-5 years
Diabetes treatment	Diet , Tablets ,		
Blood clots	None ,		
Medical treatments	Aspirin , Cholesterol lowering (Statin)		

ePAQ VASC 1.7 Minnie Mouse Completed on 27/11/2019

Name	Minnie Mouse	Date of birth	21/09/1951	NHS Number	111 222 TEST

©ePAQ systems Ltd 2020 Page 3 of 7

ePAQ electronic Personal Assessment Questionnaire **Ms Minnie Mouse - 666555 - 111222TEST - 27/11/2019** Page 4 of 7

Anxiety		Dimension skipped	
ADL		Dimension skipped	
CNS Symptoms		Dimension skipped	

**Aortic aneurysm**		**Score (0 - 100)**	**Impact**
Quality of Life		Dimension skipped	
Anxiety		Dimension skipped	
AAA Symptoms		Insufficient data	

**Lower Limb**		**Score (0 - 100)**	**Impact**
Pain	0		
Ulceration	0		
Varicose veins		Insufficient data	
ADL	35		
Claudication	62		

ePAQ VASC 1.7 Minnie Mouse Completed on 27/11/2019

Name	Minnie Mouse	Date of birth	21/09/1951	NHS Number	111 222 TEST (02)
Clinician	Mr PAD Surgeon	Clinic	Claudication Clinic	Hospital Number	666555

Generic

Consent	Yes		
Pain	Yes	Cause of pain	Poor circulation in my left leg

©ePAQ systems Ltd 2020 Page 4 of 7

ePAQ electronic Personal Assessment Questionnaire **Ms Minnie Mouse - 666555 - 111222TEST - 27/11/2019** Page 5 of 7

Clinician	Mr PAD Surgeon	Clinic	Claudication Clinic	Hospital Number	666555
Smoker	Ex-smoker (quit more than 1 year ago)	Referal reason		Condition change	
Diabetes status	Yes			BMI	24 (Height:157.48 cm) (Weight:60.32778521 kg)
Goals					
Concerns	1. Poor circulation left leg<br>2. Risk of losing my other leg				
Questions	1. What can be done to treat my poor circulation				

Generic		Score (0 - 100)	Impact
EQ-5D	35		
Pain	67		
Sensation	33		
Weakness		Insufficient data	

Carotid Artery Disease		Score (0 - 100)	Impact
Anxiety		Dimension skipped	
ADL		Dimension skipped	
CNS Symptoms		Dimension skipped	

Aortic aneurysm		Score (0 - 100)	Impact
Quality of Life		Dimension skipped	
Anxiety		Dimension skipped	
AAA Symptoms		Insufficient data	

Lower Limb		Score (0 - 100)	Impact
Pain	0		
Ulceration	0		
Varicose veins		Insufficient data	
ADL	35		
Claudication	62		

ePAQ VASC 1.7 Minnie Mouse Completed on 27/11/2019

Name	Minnie Mouse	Date of birth	21/09/1951	NHS Number	111 222 TEST (02)
Clinician	Mr PAD Surgeon	Clinic	Claudication Clinic	Hospital Number	666555

Generic

Consent	Yes		
Pain	Yes	Cause of pain	Poor circulation in my left leg
Pain - Location	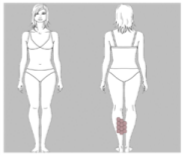		
Pain - Description	Cramping pain on walking		
Frequency of pain	Occasionally	Pain - length of time	Less than 6 months
Pain - ADL Overall impact	0 1 2 3		

©ePAQ systems Ltd 2020 Page 5 of 7

ePAQ electronic Personal Assessment Questionnaire **Ms Minnie Mouse - 666555 - 111222TEST - 27/11/2019** Page 6 of 7

Clinician	Mr PAD Surgeon	Clinic	Claudication Clinic	Hospital Number	666555
Smoker	Ex-smoker (quit more than 1 year ago)	Referal reason		Condition change	
Diabetes status	Yes			BMI	24 (Height:157.48 cm) (Weight:60.32778521 kg)
Goals					
Concerns	1. Poor circulation left leg<br>2. Risk of losing my other leg				
Questions	1. What can be done to treat my poor circulation				

Generic		Score (0 - 100)	Impact
EQ-5D	35		
Pain	67		
Sensation	33		
Weakness		Insufficient data	

Carotid Artery Disease		Score (0 - 100)	Impact
Anxiety		Dimension skipped	
ADL		Dimension skipped	

©ePAQ systems Ltd 2020 Page 6 of 7

For peer review only

ePAQ electronic Personal Assessment Questionnaire **Ms Minnie Mouse - 666555 - 111222TEST - 27/11/2019** Page 7 of 7

Clinician	Mr PAD Surgeon	Clinic	Claudication Clinic	Hospital Number	666555
Smoker	Ex-smoker (quit more than 1 year ago)	Referal reason		Condition change	
Diabetes status	Yes	BMI	24 (Height:157.48 cm) (Weight:60.32778521 kg)		
Goals					
Concerns	1. Poor circulation left leg<br>2. Risk of losing my other leg				
Questions	1. What can be done to treat my poor circulation				

Generic		Score (0 - 100)	Impact
EQ-5D	35		○
Pain	67		○
Sensation	33		○
Weakness		Insufficient data	

Carotid Artery Disease		Score (0 - 100)	Impact
Anxiety		Dimension skipped	
ADL		Dimension skipped	
CNS Symptoms		Dimension skipped	

Aortic aneurysm		Score (0 - 100)	Impact
Quality of Life		Dimension skipped	
Anxiety		Dimension skipped	
AAA Symptoms		Insufficient data	

Lower Limb		Score (0 - 100)	Impact
Pain	0		○
Ulceration	0		○
Varicose veins		Insufficient data	
ADL	35		●
Claudication	62		◔

©ePAQ systems Ltd 2020

r review only

ePAQ VASC 1.7 Minnie Mouse Completed on 27/11/2019					
Name	Minnie Mouse	Date of birth	21/09/1951	NHS Number	111 222 TEST (02)
Clinician	Mr PAD Surgeon	Clinic	Claudication Clinic	Hospital Number	666555

Smoker	Ex-smoker (quit more than 1 year ago)	Referal reason		Condition change	
Diabetes status	Yes	BMI	24 (Height:157.48 cm) (Weight:60.32778521 kg)		
Goals					
Concerns	1. Poor circulation left leg<br>2. Risk of losing my other leg				
Questions	1. What can be done to treat my poor circulation				

Generic		Score (0 - 100)	Impact
EQ-5D	35		
Pain	67		
Sensation	33		
Weakness		Insufficient data	

Carotid Artery Disease		Score (0 - 100)	Impact
Anxiety		Dimension skipped	
ADL		Dimension skipped	
CNS Symptoms		Dimension skipped	

Aortic aneurysm		Score (0 - 100)	Impact
Quality of Life		Dimension skipped	
Anxiety		Dimension skipped	
AAA Symptoms		Insufficient data	

Lower Limb		Score (0 - 100)	Impact
Pain	0		
Ulceration	0		
Varicose veins		Insufficient data	
ADL	35		
Claudication	62		

ePAQ VASC 1.7 Minnie Mouse Completed on 27/11/2019					
Name	Minnie Mouse	Date of birth	21/09/1951	NHS Number	111 222 TEST (02)
Clinician	Mr PAD Surgeon	Clinic	Claudication Clinic	Hospital Number	666555

Generic			
Consent	Yes		
Pain	Yes	Cause of pain	Poor circulation in my left leg
Pain - Location	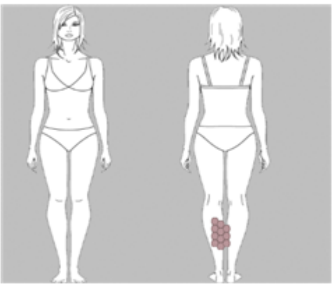		
Pain - Description	Cramping pain on walking		

Pain - Description	Cramping pain on walking		
Frequency of pain	Occasionally	Pain - length of time	Less than 6 months
Pain - ADL Overall impact	0 1 **2** 3		
Sensation change	Yes	Cause	Numb right below knee stump
Sensation change - Location	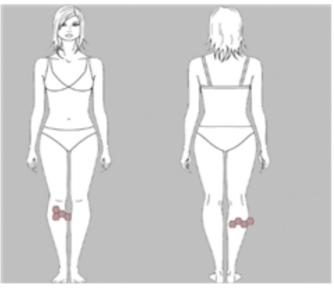		
Sensation change - Description	Stump of right leg has always been numb since my operation		
Sensation change - Frequency	All of the time	Sensation change - Duration	2 or more years
Sensation change - ADL Overall impact	0 **1** 2 3		
Weakness	No	Weakness - Cause	**##QuestionShown##**
Weakness location	—		
Weakness - Description	—		
Weakness - Frequency	—	Weakness - Duration	—
Weakness - ADL Overall impact	—		
BMI	Height:157.48cm Weight:60.33kg BMI: 24		
Smoking status	Ex-smoker (quit more than 1 year ago)	Current habit	
Smoking history	30		
Smoking history cont.	15		
Smoking habit	;		
Diabetes status	Yes	Diabetes duration	3-5 years
Diabetes treatment	Diet , Tablets ,		
Blood clots	None ,		
Medical treatments	Aspirin , Cholesterol lowering (Statin)		

only

ePAQ VASC 1.7 Minnie Mouse Completed on 27/11/2019

Name	Minnie Mouse	Date of birth	21/09/1951	NHS Number	111 222 TEST (02)
Clinician	Mr PAD Surgeon	Clinic	Claudication Clinic	Hospital Number	666555

Carotid Artery Disease

CAD Diagnosis	No	Diagnosis time frame	—
CAD - Diagnosis date			
CAD - Previous TIA	—	CAD - TIA Count	—
CAD - Most recent TIA			
CAD - First TIA			
CAD - Previous Stroke	—	CAD - Stroke Count	—
CAD - Most recent stroke			
CAD - First stroke			
CAD - TIA/Stroke Anxiety	—		
CAD - Anxiety	—		
Health anxiety	—		
Independence anxiety	—		
CAD - Balance	—		
CAD - Memory symptoms	—		
CAD - Altered speech	—		
CAD - Swallowing	—		
CAD - Vision	—		
Right eye symptoms	—		
CAD - Left eye	—		
CAD - ADL overall impact	—		
CAD - Physical activity	—		
CAD - Personal responsibilities	—		
CAD - Independent living	—		
CAD - Social activity	—		
CAD - Low mood	—		

ePAQ VASC 1.7 Minnie Mouse Completed on 27/11/2019					
Name	Minnie Mouse	Date of birth	21/09/1951	NHS Number	111 222 TEST (02)
Clinician	Mr PAD Surgeon	Clinic	Claudication Clinic	Hospital Number	666555

Aortic aneurysm

Aortic aneurysm - Diagnosis	No	Aortic aneurysm - First aware	—
Aortic aneurysm - Diagnosis date			
AAA - Family or friends	—		
AAA - Abdominal pain	—		
AAA - Throbbing	—		
AAA - Anxiety	—		
AAA - Symptom anxiety	—		
AAA - Size increase anxiety	—		
Rupture	—		
Lifting	—		
AAA - Anxiety Independent travel	—		
AAA - ADL Overall impact	—		
AAA - ADL Physical activities	—		
AAA - ADL Personal responsibilities	—		
AAA - ADL Independent living	—		
AAA - ADL Social activity	—		
AAA - ADL Low mood	—		

©ePAQ systems Ltd 2020 For peer review only - http://bmjopen.bmj.com/site/about/guidelines.xhtml

ePAQ VASC 1.7 Minnie Mouse Completed on 27/11/2019

Name	Minnie Mouse	Date of birth	21/09/1951	NHS Number	111 222 TEST (02)
Clinician	Mr PAD Surgeon	Clinic	Claudication Clinic	Hospital Number	666555

Lower Limb

Lower limb - Vascular disease	Yes	Lower limb - Vascular disease date	Over 5 years ago
Lower limb - Precise diagnosis date			
Lower limb - Treatments	Yes		
Lower limb - Procedures	Bypass graft of a blockage , Balloon stretch of a vessel ,	LL - Other procedures	—
Lower limb - Cramping pain	0 1 **2** 3	Impact	0 **1** 2 3
Lower limb - Walking	Most of the time		
Lower limb - Walking distance	0 1 2 **3**		
Lower limb - Walking speed	0 1 **2** 3		
Lower limb - Walking uphill	0 1 **2** 3		
Lower limb - Climbing stairs	0 1 **2** 3		
Lower limb - Pain at night	Never	Impact	—
Lower limb - Dangling	—		
Lower limb - pain resting	—		
Lower limb - Leg ulcers	—	Impact	—
Lower limb - Ulceration	Yes	Lower limb - Ulceration first notice	3 - 5 years
LL - Ulceration location	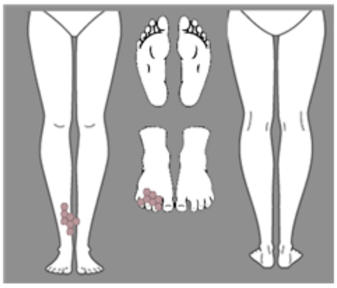		
LL - Ulceration smell	**0** 1 2 3		
LL - Ulceration appearance	**0** 1 2 3		
LL - Ulceration fluid leakage	**0** 1 2 3		
LL - Ulceration infection	**0** 1 2 3		
LL - Ulceration recurrence	No		
LL - Ulceration healing anxiety	**0** 1 2 3		
Lower limb - Varicose veins	No	Impact	—
Lower limb - Varicose veins location	—		
LL - Varicose veins bleeding	—		
LL - Varicose veins skin problems	—		
LL - Varicose veins self-consciousness	—		
Lower limb - ADL Clothing	—		
Lower limb - Swelling	—		
Lower limb - Itching	—		
Compression	—		
Lower limb - loss	Yes	Details	Leg ulcers in right leg (current problem on

			[illegible] left)
Limb loss location	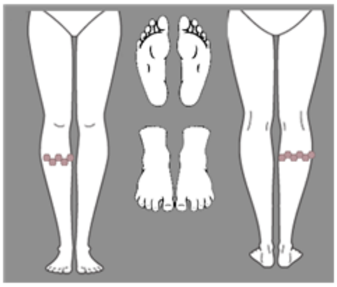		
Lower limb - Future problem anxiety	0 1 2 **3**	Impact	A serious problem
Lower limb - ADL Physical activities	A lot		
Lower limb - ADL Personal responsibilities	0 **1** 2 3		
Lower limb - ADL Independent living	0 **1** 2 3		
Lower limb - ADL Social activities	0 **1** 2 3		
Lower limb - Help with daily living	No, I don't need any help	Lower limb - Sufficient support	Entirely
Sex life	0 **1** 2 3		
ADL low mood	0 **1** 2 3		

peer review only

ePAQ VASC 1.7 Minnie Mouse Completed on 27/11/2019					
Name	Minnie Mouse	Date of birth	21/09/1951	NHS Number	111 222 TEST (02)
Clinician	Mr PAD Surgeon	Clinic	Claudication Clinic	Hospital Number	666555

Understanding the ePAQ Report

Disclaimer

The information provided by ePAQ® is intended for guidance only and should not be used in isolation. It should only be used in conjunction with other assessments of health, including previous and current medical history, examination findings and the results of relevant investigations. Some of these assessments may only be available by consulting a general practitioner or specialist. The symptoms scores provided are also only a guide to possible conditions or causes of underlying symptoms. They do not exclude serious illness, nor do they confirm the presence of any specific disease or abnormality. ePAQ Systems Ltd is not responsible or liable for any form of damages whatsoever resulting from the use (or misuse) of information contained in, generated by or implied by this questionnaire and associated software.

When interpreting reports, it should always be borne in mind that errors sometimes occur in understanding, transcribing, transferring or storing questionnaires, information and data. Subjects who are concerned about any aspect of their health (whether or not covered by this questionnaire) are strongly advised to seek medical advice, irrespective of the results of the results of this questionnaire.

Understanding ePAQ analysis & symptoms scores

This section provides clinically meaningful domain scores for related symptoms in each dimension The clock face to the right of each domain score indicates the maximum severity of any of the symptoms that contribute to that domain score: Empty circle = 'Not a problem', 1/3 circle = 'A bit of a problem', 2/3 circle = 'Quite a problem', full circle = 'A serious problem'.

Information about the domain score and related medical conditions can be found in the information links (reached by pressing the clock face icon to the right of the domain score itself). Each item within a domain contributes equally to the final domain score.

Domain scores are presented on a scale of 0 - 100: Low score = absent or minimal symptoms, high score = frequent or severe symptoms (0 = minimum symptoms, 100 = maximum symptoms). If any data are missing, a score is not calculated for that domain. Items that do not contribute to a domain score can be viewed in the Itemised Report

Key To Symbols

-	=	Skipped automatically			
x	=	Declined to answer			
**Symptom frequency / Severity (left hand column)**			**Impact (right hand column)**		
0	=	Never / Not at all	0	=	Not a problem
1	=	Occasionally / A little	1	=	A bit of a problem
2	=	Most of the time / Moderately	2	=	Quite a problem
3	=	All of the time / A lot	3	=	A serious problem

only

BMJ Open

Mixed methods study to develop the content validity and the conceptual framework of the electronic patient-reported outcome measure for vascular conditions

Journal:	*BMJ Open*
Manuscript ID	bmjopen-2019-034154.R2
Article Type:	Original research
Date Submitted by the Author:	05-Jun-2020
Complete List of Authors:	Aber, Ahmed; The University of Sheffield, ScHARR<br>Phillips, Patrick; The University of Sheffield, ScHARR<br>Lumley, Elizabeth; The University of Sheffield, ScHARR<br>Radley, Stephen; Sheffield Teaching Hospitals NHS Foundation Trust<br>Thomas, Steve; The University of Sheffield, ScHARR<br>Nawaz, Shah; Sheffield Teaching Hospitals NHS Foundation Trust, Sheffield Vascular Institute<br>Jones, Georgina; Leeds Beckett University Faculty of Health and Social Sciences,<br>Michaels, Jonathan; University of Sheffield, ScHARR
**Primary Subject Heading**:	Surgery
Secondary Subject Heading:	Health policy, Health services research, Qualitative research, Surgery, Cardiovascular medicine
Keywords:	Patient reported outcome measures, Vascular surgery < SURGERY, electronic health record (EHR), Quality in health care < HEALTH SERVICES ADMINISTRATION & MANAGEMENT, QUALITATIVE RESEARCH, Mixed methods

SCHOLARONE™
Manuscripts

I, the Submitting Author has the right to grant and does grant on behalf of all authors of the Work (as defined in the below author licence), an exclusive licence and/or a non-exclusive licence for contributions from authors who are: i) UK Crown employees; ii) where BMJ has agreed a CC-BY licence shall apply, and/or iii) in accordance with the terms applicable for US Federal Government officers or employees acting as part of their official duties; on a worldwide, perpetual, irrevocable, royalty-free basis to BMJ Publishing Group Ltd ("BMJ") its licensees and where the relevant Journal is co-owned by BMJ to the co-owners of the Journal, to publish the Work in this journal and any other BMJ products and to exploit all rights, as set out in our licence.

The Submitting Author accepts and understands that any supply made under these terms is made by BMJ to the Submitting Author unless you are acting as an employee on behalf of your employer or a postgraduate student of an affiliated institution which is paying any applicable article publishing charge ("APC") for Open Access articles. Where the Submitting Author wishes to make the Work available on an Open Access basis (and intends to pay the relevant APC), the terms of reuse of such Open Access shall be governed by a Creative Commons licence – details of these licences and which Creative Commons licence will apply to this Work are set out in our licence referred to above.

Other than as permitted in any relevant BMJ Author's Self Archiving Policies, I confirm this Work has not been accepted for publication elsewhere, is not being considered for publication elsewhere and does not duplicate material already published. I confirm all authors consent to publication of this Work and authorise the granting of this licence.

For peer review only

Mixed methods study to develop the content validity and the conceptual framework of the electronic patient-reported outcome measure for vascular conditions

1) Authors: Ahmed Aber[a], Patrick Phillips[a], Elizabeth Lumley[a], Stephen Radley[b], Steve Thomas[c], Shah Nawaz[c], Georgina Jones[d], Jonathan Michaels[a]

Ahmed Aber, University of Sheffield, a.aber@sheffield.ac.uk
Patrick Phillips, University of Sheffield, p.phillips@sheffield.ac.uk
Elizabeth Lumley, University of Sheffield, e.lumley@sheffield.ac.uk
Stephen Radley, Sheffield Teaching Hospitals, Stephen.Radley@sth.nhs.uk
Steve Thomas, Sheffield Teaching Hospitals, s.m.thomas@sheffield.ac.uk
Shah Nawaz, Sheffield Teaching Hospitals, shah.nawaz@sth.nhs.uk
Georgina Jones, Leeds Beckett University, g.l.jones@leedsbeckett.ac.uk
Jonathan Michaels, University of Sheffield, j.michaels@sheffield.ac.uk

2) [a]Institution: School of Health and Related Research, University of Sheffield, Sheffield
[b]Institution: Jessop Wing, Sheffield Teaching Hospitals, Sheffield
[c]Institution: Sheffield Vascular Institute, Sheffield Teaching Hospitals, Sheffield
[d]Institution: Department of Psychology, School of Social Sciences, Leeds Beckett University, Leeds

3) *Corresponding author: Ahmed Aber, Regent Court, 30 Regent Street, School of Health and Related Research, University of Sheffield, Sheffield, S1 4DA.
E-mail: a.aber@sheffield.ac.uk.
Tel: +44 (0) 114 222 4029
Fax: +44 (0) 114 222 0740

4) Category for manuscript submission: Original Research

5) Source of funding for research and publication: This manuscript presents independent research funded by the National Institute for Health Research (NIHR) under the Programme Grants for Applied Research (RP-PG-1210-12009).
The views expressed are those of the authors and not necessarily those of the NHS, the NIHR or the Department of Health.

6) The authors thank the UK Vascular PROMs Group and Cardiovascular Research Patient Panel at Sheffield Teaching Hospitals NHS Foundation Trust.

7) Conflict of interest: Stephen Radley is a shareholder in ePAQ Systems Ltd, an NHS spin-out technology company, the majority shareholder being Sheffield Teaching Hospitals. The other authors have no conflict of interest.

For peer review only

UK Vascular PROMs Group:

Ms. Alison Abbott, Vascular Clinical Nurse Specialist.
Mr. Ayman Badawy, Vascular Surgery Associate Specialist.
Mr. Nasim Akhtar, Vascular Consultant Surgeon.
Mr. Faisal, Alam, Vascular Consultant Surgeon.
Mr. Wissam Al-Jundi, Vascular Consultant Surgeon.
Mr. Matthew Armon, Vascular Consultant Surgeon.
Mr. Lukla Biasi, Vascular Consultant Surgeon.
Professor Matt Bown, Vascular Consultant Surgeon.
Professor Bruce Campbell, Vascular Consultant surgeon.
Mr. Tommaso Donati, Vascular Consultant Surgeon.
Mr. Tony Fox, Vascular Consultant Surgeon.
Mr. Andrew Gordon, Vascular Consultant Surgeon.
Professor Linda Hands, Vascular Consultant Surgeon.
Mr. Gary Hicken, Vascular Consultant Surgeon.
Mr. Peter Holt, Vascular Consultant Surgeon.
Dr. Shelley Jackson, General Practitioner.
Ms. Brenda King, Specialist Nurse.
Ms. Talia Lea, Vascular Clinical Nurse Specialist.
Mr. Greg McMahon, Vascular Consultant Surgeon.
Mr. John Mosley, Vascular Consultant Surgeon.
Mr. Sanjay Patel, Vascular Consultant Surgeon.
Dr. Gaynor Radley, General Practitioner.
Mr. Harjeet Rayt, Vascular Consultant Surgeon.
Professor Rob Sayers, Vascular Consultant Surgeon.
Mr. Paul Tisi, Vascular Consultant Surgeon.

Keywords:

Vascular disease, patient-reported outcome measures, quality of life, content validity, qualitative research, electronic questionnaire.

For peer review only

Abstract

Objective:

The aim of this paper is to describe the stages undertaken to generate the items and conceptual framework of a new electronic personal assessment questionnaire for vascular conditions.

Design:

A mixed methods study: a clinician survey followed by quantitative systematic reviews to identify validated patient reported outcome measures (PROMs). A qualitative study of patients and systematic reviews of the qualitative evidence were used to develop this new PROMs. This was followed by a quantitative clinicians' consensus study and finally, a qualitative face validity study with patients.

Participants:

Vascular patients participated in the primary qualitative study and the face validity study. In the qualitative study fifty-five patients were interviewed and for the face validity nineteen patients gave feedback. Twelve clinicians completed the survey and thirteen completed two cycles of the clinicians' consensus study.

Results:

The items and scales in the electronic personal assessment questionnaire for vascular conditions (ePAQ-VAS) were generated based on the results of five systematic reviews evaluating existing PROMs for possible inclusion in ePAQ-VAS, five systematic reviews of qualitative evidence, a primary qualitative study involving 55 patients and clinicians' input. One hundred and sixty-eight items were initially generated; of which 59 were eliminated by the expert panel due to repetition. The instrument was divided into one generic, and three disease-specific sections (abdominal aortic aneurysm (AAA), carotid artery disease (CAD), and lower limb vascular conditions). In each section, items were grouped together into putative scales. Fifty-five items were grouped across eight scales; the remaining items were kept as individual items, because of relevance to service users.

Conclusions:

This multi-dimensional electronic questionnaire covers the most common vascular conditions. This is particularly important for patients presenting with mixed symptoms or multiple conditions. This tool captures symptomatology, HRQoL and other clinically relevant data, such as experience with services and co-morbidities.

For peer review only

Strengths and limitations of the study

- This electronic patient assessment questionnaire for vascular conditions was developed with input from patients and clinicians.
- The themes generated from previously published five comprehensive qualitative reviews and a qualitative study of vascular patients were used to develop the items.
- Vascular clinicians were surveyed to ensure clinically relevant conditions and questions were included.
- The burden of questionnaire is its main limitation; however, providing strict skipping rules only relevant questions are presented to patients.
- The face validity study examined the clarity and relevance of the items, however, the comprehensiveness this PROMs was not assessed.

For peer review only

Introduction

Vascular conditions can cause problems throughout the body; epidemiological studies suggest that both venous and arterial diseases are very common **(1, 2)**. It therefore makes sense to assess individuals with vascular disease holistically; investigating not only existing or potential manifestations of vascular disease, but also the impact of conditions on health-related quality of life. PROMs are questionnaires or instruments, designed to elicit information directly from the patient and can be used as part of such an assessment.

Validity and reliability are integral to developing or selecting a PROMs. A key aspect of validity is *content validity,* and international guidelines including the U.S. Food and Drug Administration (FDA) guidance stress the importance of this psychometric property **(3)**.

Many generic and condition specific PROMs have been adopted to examine impact of vascular conditions on patients and measure outcomes. This is despite a lack of evidence that they have been developed and evaluated in-line with accepted guidelines; in addition, these instruments are rarely used or formally evaluated in routine patient assessment in day to day clinical practice. We conducted scoping searches and informal discussions with vascular clinicians to identify any existing PROMs, however, these preliminary stages in the research process suggested an absence of valid and reliable PROMs for use in vascular populations.

In this paper, we report the stages in developing an electronic personal assessment questionnaire for vascular patients (ePAQ-VAS). This includes:

(i) Identifying the main vascular conditions to be included in this electronic measure based on a survey of clinicians treating vascular disease.

(ii) Developing a hypothesized framework for the sections for different disease categories based on the previous systematic reviews that identified PROMs used in patients with abdominal aortic aneurysm (AAA), carotid artery disease (CAD), peripheral arterial disease (PAD), venous leg ulcers (VLU) and varicose veins (VV) **(4-8).**

(iii) Developing the items within each section of ePAQ-VAS based on qualitative systematic reviews **(9-12),** and a primary qualitative study **(13).**

(iv) A consensus study with clinicians to rate the relevance of included items, and to add items to ePAQ-VAS based on the opinion of vascular surgeons, radiologists and nurses.

(v) A face validity study with vascular patients to examine the clarity and relevance of the items within ePAQ-VAS.

The aim of these steps was to develop a single electronic instrument covering most vascular conditions in line with international guidance **(3)**. The conceptual

For peer review only

framework and items were developed in a way to ensure this assessment tool can be used in patients with mixed symptoms and multiple vascular conditions. Every patient to receive a unique voucher code along with their clinic letters. The code can be used to access and complete ePAQ-VAS at home or in the outpatient clinic using computers or other electronic devices.

The server of ePAQ is hosted and integrated with NHS N3-based informatics systems. Other ePAQ questionnaires such as ePAQ-Pelvic floor and ePAQ- pre-assessment are in clinical use in different NHS hospitals. ePAQ Ltd. is an NHS spin-out technology company and the patient data collected by the company can be linked to the unique NHS number of each patient; and although there is a lack of integrated digital infrastructure in the NHS; the technology is available for future use to link records collected by different NHS providers

Methods

Clinicians involved in the care of vascular patients were invited to identify the common vascular conditions treated by vascular surgeons and vascular specialists. They were asked to list the key issues, symptoms and the impact of these conditions on patients suffering with these diseases. Data from this round were used to inform qualitative evidence synthesis.

The conceptual framework of ePAQ-VAS was based on primary qualitative interviews with vascular patients, input from clinicians, systematic reviews examining the validity of existing PROMs, and qualitative reviews of the impact of vascular diseases on quality of life. Ethical approval was obtained from Yorkshire & Humber NRES committee– Bradford Leeds (REC Number: 14/YH/1117) on 25.09.2014. Figure 1. Illustrates the process used to develop ePAQ-VAS in accordance to FDA guidelines **(3)**.

Figure 1. Development of ePAQ-VAS conceptual framework

Insert figure 1 here

For peer review only

Systematic Reviews to Identify and Appraise Existing PROMs

Systematic searches were conducted of bibliographic databases including CINAHL via EBSCO, MEDLINE and MEDLINE in Process via Ovid, EMBASE via Ovid, PsycINFO via Ovid, Social Science Citation Index/Science Citation Index via Web of Science (Thomson Reuters), and Proquest dissertations and theses. PROMs were included where there was evidence that they had undergone some form of psychometric evaluation that would allow the validity, reliability and responsiveness of the PROMs to be assessed. Included PROMs were categorised per type, (generic or condition specific) and the vascular population(s) in which they had been validated. Quality assessment **(3, 14)** was conducted to identify high quality existing PROMs for possible direct inclusion in ePAQ-VAS, or to be used as a basis to inform the qualitative evidence synthesis. For further information about the appraisal criteria to examine the robustness of the psychometric analysis and samples of search strategies please see the supplementary materials.

Primary qualitative study

Semi-structured interviews were conducted with 55 vascular patients from Sheffield Teaching Hospitals NHS Foundation Trust (STH) following purposeful sampling to ensure a range of participants of different age and gender, at different stages of treatment and covering the main five vascular conditions (AAA, PAD, CAD, VLU, VV). A consultant vascular surgeon or specialist nurse approached each patient either in clinic or over the telephone to explain about the project and ask if the patient would be interested in participating in the study. If the initial approach was by the clinician in clinic, the researcher would then speak to the patient and give further information about the project including a participant information sheet (PIS) before taking contact details. For those patients that were first contacted over the phone the clinician would then gain verbal consent to pass on their contact details to a researcher. Copies of the PIS were sent out through the mail to those who had not been initially approached in clinic. The researcher gave at least 24 hours for the patient to read through the PIS and consider the information before contacting each person by telephone to ask if they would be interested in participating in an interview. If they were interested in taking

For peer review only

part a date and time was agreed for a researcher to visit the participant at home to carry out an interview. Questions were asked about the signs, symptoms, and impact of the condition on function and lifestyle. On the day of the interview the trained qualitative researcher checked the participant understood the PIS and took informed written consent. Field notes were taken to aid interpretation of the interview data. Each interview was recorded and transcribed verbatim. Personal details were removed from the transcript to enhance participant anonymity. The interview transcripts were typed and uploaded into NVIVO 11 (QSR International, Warrington, UK) for management and analysis.

Systematic reviews of the qualitative evidence

Systematic searches of the following databases; CINAHL via EBSCO, MEDLINE and MEDLINE in Process via Ovid, EMBASE via Ovid, PsycINFO via Ovid, Social Science Citation Index/Science Citation Index via Web of Science (Thomson Reuters), and Proquest dissertations were conducted to identify existing qualitative research detailing vascular patients' experience of living with AAA, PAD, CAD, VLU, and VV. For further samples of search strategies please see the supplementary materials.

Analysis of the qualitative evidence

Qualitative data from the primary study and each of the systematic qualitative reviews were analysed separately. Framework analysis was used to analyse the interviews **(15).** This analysis includes five stages,

- the first involved familiarization by reading of the transcripts and reading the primary data.
- The second stage involved identification of a thematic framework; the thematic framework was based either on clinical opinion for areas with no valid PROMs, such as AAA and CAD, or a combination of clinical opinion and, when available, the scales of PROMs with good content validity.
- In the third stage the data was coded and indexed by applying the thematic framework to the whole data set until saturation was achieved. Anindependent

For peer review only

researcher checked all the themes that were identified, and differences in were discussed and adjusted involving a third senior author (GJ)

- At the fourth stage, a framework matrix was created by arranging the data per the thematic references.
- finally mapping and interpretation, including examining patterns within the data and associations with it.

Clinicians input and consensus exercise

Twenty-three clinicians involved in the care and management of patients with vascular conditions were invited to a survey to list the most common vascular conditions managed by them and to list the key issues, symptoms and the impact of these diseases on patients. Data from this round were used to inform qualitative evidence synthesis.

Different group of clinicians involved in the care of vascular patients were invited to a consensus study to score the relevance of items (questions) in the provisional version of ePAQ-VAS. In total, thirty clinicians including vascular surgeons, interventional radiologists, vascular nurses, physiotherapists and occupational therapists were invited. Participants were asked to rate the appropriateness of each question on a five-point Likert scale of "Strongly disagree" (=0) to 'Strongly agree' (=4). This process was repeated, and members of the clinicians' panel were presented with the aggregate findings of the previous round, and again asked to score each question. This process aimed to examine the relevance of each item from the clinicians' perspective and to identify any new items suggested by the clinicians **(16)**.

Developing scales and items

The ePAQ development team (AA, EL, PP, GJ, SR) employed an iterative process, incorporating evidence from the systematic reviews, qualitative study, and the clinicians' consensus study. In line with the FDA guidance **(3)**, items (questions) were developed from the qualitative data using the following three steps: interpretation, translation, and triangulation of themes.

For peer review only

For peer review only - http://bmjopen.bmj.com/site/about/guidelines.xhtml

Interpretation involved familiarisation with the language used in the primary data included in the synthesis. This enabled translation of descriptions of apparently diverse issues affecting vascular patients into a single set of harmonised themes. The resulting themes were used to develop the items for ePAQ-VAS. The items were grouped into sections, and each section, further divided into scales consisting of a connected group of items. Triangulation was performed across evidence sources to ensure the items comprehensively covered all issues of importance to patients with AAA, PAD, CAD, VLU, and VV.

Face validity of ePAQ-VAS

A second phase of semi-structured patient interviews was conducted by (EL, PP) with 19 participants, purposefully sampled from the vascular populations previously described. This sample included patients with AAA, CAD, PAD, VLU and VVs. ePAQ-VAS (version 1) was presented to these patients and a focused interview was conducted to investigate vascular patients' perceptions of the questionnaire in its entirety as well as the relevant items to the individual being interviewed. Questions were asked under the following headings of:

- overall impressions,
- clarity,
- relevance and emotional response.

Interviews were audio taped, transcribed and analysed. A pragmatic approach was used for the analysis, with comments collated and presented back to the working group who made consensus decisions on revisions to ePAQ-VAS. Written consent was obtained from the participants.

Patient and Public involvement:

The research question and output was developed in consultation with patients and public. The authors thank the Cardiovascular Research Patient Panel at Sheffield Teaching Hospitals NHS Foundation Trust. The aim of the research was to develop a patient focused outcome measure. In this process patients were recruited for two qualitative studies to ensure content validity and face validity of this tool. Patients

For peer review only

were involved in every stage of the development of the study. The developed electronic patient assessment questionnaire for vascular conditions (ePAQ-VAS) have been used by patients in a clinical study and there are plans for regular clinical use. The results will also be disseminated in relevant meetings and among patient groups.

Results

In total twelve clinicians completed the first survey and identified PAD, AAA, VLU, VV and CAD as the most common vascular conditions treated by them. They listed common issues such as pain on walking, rest pain, reduced mobility or lack of mobility for patients with PAD, no physical symptoms for those with AAA but need for multidisciplinary approach to manage these patients. For patients with VLU, the main issues included burning pain, recurrence and healing; for patients suffering with VV, skin changes, appearance of leg and ulcer as well as ache were the main issues raised. The clinicians felt the key issue for patients with CAD was identifying patients benefiting from intervention and reducing the risk of stroke. The result from this survey was used to inform the analysis of qualitative data used to develop ePAQ-VAS.

Systematic reviews and assessment of psychometric evaluation were conducted for PROMs validated for use in PAD, AAA, VLU, VV and CAD. A total of 33 PROMs that had undergone some form of validation were identified in 41 studies **(Table 1.)**.

Condition	Number of Citations	Number of Included Papers	Results	Conclusions
AAA	1232	3	4 validated PROMs identified –1 generic, 1 vascular generic and 2 condition-specific	This review has highlighted a gap in the evidence for validated PROMs in AAA. Due to a lack of rigorous psychometric testing.
CAD	1670	5	6 validated PROMs identified –	There was a lack of validated PROMs to measure outcomes for CAS patients.

For peer review only

			4 generic and 2 condition-specific	
PAD	6981	14	13 validated PROMs identified – 6 generic and 7 condition-specific	VascQol was the most psychometrically robust instrument.
VV	3879	7	3 validated PROMs identified – 1 generic and 2 condition-specific	Aberdeen Varicose Vein Questionnaire is the most psychometrically robust disease specific PROMs for use with VV patients.
VLU	3879	6	7 validated PROMs identified – 3 generic and 4 condition-specific	The most valid and reliable condition specific PROMs was VLU-QOL.
Abbreviations: Abdominal aortic aneurysm (AAA), Carotid artery disease (CAD), Peripheral arterial disease (PAD), Venous leg ulcer (VLU), Varicose veins (VV).				

Table 1: Results from the systematic reviews of psychometric evaluation of vascular PROMs

No PROMs were identified that had undergone sufficiently rigorous development and validation to suggest that they were suitable for direct use in ePAQ-VAS, the details of these reviews have been reported previously **(4-8).** Where evidence existed, this fell short of required standards **(3,14)**. For instance, the review investigating VV PROMs **(4)** found some evidence for, and discussion of, content validity in relation to the Aberdeen varicose vein questionnaire (AVVQ) and suggest that it is the most appropriate existing condition specific measure for use in a VV population. However, item generation for this PROMs involved a literature review and assessment by clinicians of relevance of included items with no direct involvement of patients, therefore suggesting a deficiency in terms of content validity **(17)**. The scales from these reviews were used to provide a framework for the systematic qualitative reviews and the primary qualitative study **(5, 9-13)**.

For peer review only

In total 111 patients were approached but only fifty-five patients (69.1% male) were interviewed about their experience of living with vascular disease, ages ranged from 35-77 years. For further information about the study participants please see table 2.

	AAA	CAD	PAD	VV	VLU	TOTAL
Gender n(%):						
Male	10(77)	5(56)	11(79)	5(50)	8(80)	39(70)
Female	3	4	3	5	2	17
Age Range	53-87	52-86	47-82	35-77	47-84	35-87
(mean)	(72)	missing	(69)	(50)	(59)	missing

Table 2: Participant characteristics of the primary qualitative study

Six overarching themes relating to the impact of the five vascular conditions were identified. These were symptoms (including pain), impact on physical function, social impact, psychological impact, financial impact and lifestyle. Pain and mobility were the most commonly reported themes by participants with PAD. The extent to which they impacted daily living was dependent on the severity of the disease, age expectations and social support. Fear of symptoms worsening and future amputation had a significant impact on daily living.

Most participants with AAA reported having no physical symptoms, a small number of participants reported abdominal pain and pain in their legs. Uncertainty, anxiety and fear of sudden death had the most impact on their quality of life. This was similar for patients with CAD who reported few lasting symptoms since the majority had what they described as a “mini-stroke”. However, CAD patients reported the widest range of signs and symptoms, with nine different symptoms. This condition had the least impact on physical and social function, although psychologically it caused a sense of worry and anxiety. This was mainly caused by fear of having a major stroke.

Pain was the most common issue reported by patients with VVs, other issues included swelling of the legs and the impact of this on mobility. The perceived unpleasant

For peer review only

appearance of the VV seemed to have had the greatest psychological impact, and was described by many of the participants. The impact of VLU on daily living and quality of life differed within the group that was interviewed. For some there were no major issues and having a VLU was accepted as part of their life, with the hope that it would heal eventually. For others, there was a far more significant effect with reports of severe sharp pain that significantly reduced their quality of life. This had a bearing on people's mobility and their ability, or desire, to go out and socialise. Sleep was also disturbed due to pain. The progression of VLU had resulted in participants suffering for long periods of time. In addition, the non-healing or reoccurring nature of the condition had a significant impact for many. VLU appeared to have a significant psychological impact causing a high degree of distress for some patients. Summary results are shown in **Table 3**.

Condition	Sample size	Key Findings
AAA	13	No physical symptoms, a small number of participants reported abdominal pain and pain in their legs. Uncertainty, anxiety and fear of rupture and death appeared to impact most greatly on people's QoL.
CAD	9	This condition seemed to have had the least impact on physical and social function, although psychologically it created a sense of worry and anxiety for some participants. The main reported outcome was fear of having a major stroke.
PAD	14	Pain and mobility were the most commonly reported themes. The extent to which they impacted on QoL was associated with the severity, age expectations and social support. Fear of the symptoms worsening and amputation was evident.
VV	10	VV do not appear to have had a major impact on overall QoL for most the participants. Pain was the most common issue. The perceived unpleasant appearance of the VV seemed to have the greatest psychological impact. Many of the participants had had their VV for very long periods of time, often just "putting up with it'' for numerous years before seeking help.
VLU	10	The impact of VLU on QoL differed within the group. For some there were no major issues and having a VLU was accepted as part of their current life, with the hope that it would heal eventually. For others, there was a far more significant effect. Pain was quite severe for some

For peer review only

		participants leading to a significantly reduced QoL. VLU appeared to have a significant psychological impact causing a high degree of distress for some.
Abbreviations: Abdominal aortic aneurysm (AAA), Carotid artery disease (CAD), Peripheral arterial disease (PAD), Venous leg ulcer (VLU), Varicose veins (VV).		

Table 3: Findings from the primary qualitative study with vascular patients

Identified signs, symptoms and impact of the conditions were then mapped and tabulated to see which themes were relevant to which condition, and where the similarities and differences lay **(Table 4.)**.

	PAD	AAA	CAD	VV	VLU
**Symptoms**					
No symptoms		x	x		
Pain	x	x	x	x	x
Neck pain			x		
Leg pain	x	x	x	x	x
Abdominal pain	x	x			
Arm pain	x				
Cramp/ aching	x	x		x	x
Burning sensation					x
Pain severity	x	x	x	x	x
Pain on walking	x	x		x	x
Pain at rest	x			x	
Pain when standing				x	x
Mobility	x	x	x	x	x
Distance	x	x		x	x
Speed	x	x			
Stairs/ slopes	x	x			
Non-healing wounds	x				x
Co-morbidities	x	x	x		x

Progression of symptoms	x	x		x	x
Sleep	x		x	x	x
Swelling			x	x	
Loss of balance			x		
Confusion			x		
**Impact on physical functioning**					
Hobbies	x			x	x
Exercise	x	x		x	x
Daily activities	x				x
**Social impact**					
Travel	x	x	x		
Social activities	x	x		x	x
Social support	x	x	x		
**Psychological impact**					
Anxiety	x	x	x	x	x
Depression	x	x			x
Feelings of loss	x			x	x
Health expectations	x	x	x	x	x
Unsightly appearance				x	
Feeling self-conscious	x			x	x
Fear of worsening symptoms	x	x	x	x	x
Fear of rupture death		x			
Fear of amputation				x	x
Fear of stroke			x		
**Financial impact**					
Income	x	x			x
Time off work				x	x
**Lifestyle**					
Smoking	x	x	x	x	x

For peer review only

Exercise	x	x	x	x	
Diet	x	x	x		x
Weight				x	x
Abbreviations: Abdominal aortic aneurysm (AAA), Carotid artery disease (CAD), Peripheral arterial disease (PAD), Venous leg ulcer (VLU), Varicose veins (VV).					

Table 4: Map of symptoms and Quality of life concepts across five conditions.

A total of 31 studies were included across the five reviews of existing qualitative research **(6, 10-13)**. A short summary of the main themes to emerge for each condition is shown in **Table 5**.

Condition	Numbers of Citations	Number of Included Studies	Key Themes
AAA	315	3	Anxiety and *lack* of physical symptoms.
CAD	964	3	Symptoms, psychological and social impact, risk and service experience.
PAD	973	9	Pain, compromised physical function, impact on social life.
VV	1804	3	Adaptation – coping strategies employed to limit various impacts, appearance of VV.
VLU	1804	13	Pain, odour and exudate - impact on sleep, mobility and mood.
Abbreviations: Abdominal aortic aneurysm (AAA), Carotid artery disease (CAD), Peripheral arterial disease (PAD), Venous leg ulcer (VLU), Varicose veins (VV).			

Table 5: Results from Qualitative Reviews examining the impact of the major vascular conditions on quality of life

The themes from the first round of the clinicians' consensus study, as well as scales of identified PROMs, were used to inform the framework analysis of the qualitative

For peer review only

data. Items from existing PROMs were then mapped against emerging themes from the qualitative study, and the qualitative review synthesis for each condition, to explore which PROMs items or scales captured themes deemed to be the most pertinent to patients. A triangulation approach was followed, whereby researchers evaluated whether the concepts were the same (agreement), offered similar concepts (partial agreement), were in contradiction (dissonance) or were not present (silence). An example of this triangulation approach is provided in **the supplementary material**. The results of the triangulation study were only used to group symptoms together and avoid repetition. No items were deleted based on the triangulation.

The ePAQ-VAS development team used the findings from the triangulation for AAA, PAD, VV, VLU and CAD to develop themes for distinct sections relevant for each of these vascular conditions. The primary qualitative data were used to create each item. Items were then grouped into sections and within each section there were scales consisting of items that measured the same latent variable such as anxiety related to the diagnosis of AAA. The results of the clinicians' consensus study were considered to add further items to the relevant sections **(Table 6)**.

The items of ePAQ-VAS were arranged into four sections; generic, AAA, CAD and lower limb (LL) vascular conditions. A single LL section was developed as common themes were identified for conditions affecting the legs, regardless of whether the underlying pathology was venous or arterial. An inclusive approach to development was used and a comprehensive questionnaire was produced with 168 questions. (See Figure 2 for an overview of the process to develop ePAQ-VAS).

For peer review only

Section	Scale	Clinicians' consensus Study	Most valid Condition Specific PROMs			Qualitative study					Qualitative Reviews					Question Text
			VLU-QOL	PADQOL	AVVQ	AAA	CAD	VV	VLU	PAD	AAA	CAD	VV	VLU	PAD	
Generic	Pain	4	X	X	X	X	X	X	X	X	X	X	X	X	X	Do you suffer with any pain?
Generic	Pain	4	X	-	-	X	X	X	X	X	X	X	X	X	X	Use the below Image and click on body parts where you experience pain or discomfort.
Generic	Pain	-	X	-	-	X	X	X	X	X	X	X	X	X	X	Please use your own words to describe this problem.
Generic	Pain	4	X	X	X	X	X	X	X	X	X	X	X	X	X	How often do you experience a significant amount of pain?
Generic	Pain	4	-	-	-	X	X	X	X	X	X	X	X	X	X	How much do problems caused by pain affect your overall enjoyment of life?
Generic	Sensation	4	-	-	-		X	X		X	X	X	X		X	Do you experience any numbness or pins and needles in any part of your body?
Generic	Sensation	4	-	-	-		X	X		X	X	X	X		X	Please use the image below to select where you experience sensation change in your body.
Generic	Sensation	-	-	-	-		X	X		X	X	X	X		X	Please use your own words to describe this problem.
Generic	Sensation	4	-	-	-		X	X		X	X	X	X		X	How often do you experience numbness or pins and needles?
Generic	Sensation	4	-	-	-		X	X		X	X	X	X		X	How much do problems caused by numbness or pins & needles affect your overall enjoyment of life?
Generic	Weakness	4	-	-	-						X	X			X	Do you have any loss of strength or weakness in any part of your body?
Generic	Weakness	3	-	-	-						X	X			X	Please use the image below to indicate the areas where you experience any physical weakness.
Generic	Weakness	-	-	-	-						X	X			X	Please use your own words to describe this problem.
Generic	Weakness	4	-	-	-						X	X			X	How often do you experience loss of strength or weakness?
Generic	Weakness	3	-	-	-						X	X			X	How much do problems caused by weakness affect your overall enjoyment of life?
Carotid	Anxiety	4					X					X				Do you worry about having a stroke?
Carotid	Anxiety	3					X					X				Does carotid artery disease make you feel anxious?
Carotid	Anxiety	New item														Are you worried about your health getting worse because of carotid artery disease?
Carotid	Anxiety	New item														Are you worried about losing your independence because of carotid artery disease?
Carotid	Symptoms	2					X									Do you have any problems with maintaining your balance?
Carotid	Symptoms	2					X									Do you suffer with any problems with your memory? (E.g. Forgetting or losing things)

Carotid		Symptoms	4					X					X				Have you had any problems with your speech? (E.g. Slurring your words or not being able to speak or say things properly)
Carotid		Symptoms	New item														Do you have any problems with swallowing food?
Carotid		Symptoms	4					X					X				Have you had any problems with partial or complete loss of vision in either of your eyes?
Carotid		Symptoms	4					X					X				How would you describe any loss of vision in your RIGHT eye?
Carotid		Symptoms	4					X					X				How would you describe any loss of vision in your LEFT eye?
Carotid		ADL	3					X					X				How much do problems caused by carotid artery disease (anxiety associated with diagnosis or physical symptoms) affect your overall enjoyment of life?
Carotid		ADL	4					X					X				How much do problems caused by carotid artery disease (e.g. mini-stroke or stroke memory balance speech visual or other related issues) affect your physical activities such as exercise walking or running?
Carotid		ADL	3					X					X				How much do problems caused by carotid artery disease (e.g. mini-stroke or stroke memory balance speech visual or other related issues) affect your ability to undertake personal roles and responsibilities such as caring for others study or work?
Carotid		ADL	4					X					X				How much do problems caused by carotid artery disease (e.g. mini-stroke or stroke memory balance speech visual or other related issues) affect your ability to look after yourself?
Carotid		ADL	3					X					X				How much do problems caused by carotid artery disease (e.g. mini-stroke or stroke memory balance speech visual or other related issues) affect your social activities such as visiting friends and family?
Carotid		ADL	2					X					X				How much do problems caused by carotid artery disease visual or other related issues) affect your mood?
AAA		Symptoms	4				X					X					Do you have any abdominal (tummy) pain?
AAA		Symptoms	New item (3)														Do you experience a throbbing feeling in your abdomen (tummy)?
AAA		Anxiety	4				X					X					Do you worry about aortic aneurysm?
AAA		Anxiety	4				X					X					Do you worry about any symptoms you experience that may be caused by aortic aneurysm?
AAA		Anxiety	4				X					X					Do you worry about possible increase in the size of your aneurysm?
AAA		Anxiety	4				X					X					Do you fear sudden death or rupture of your aortic aneurysm?
AAA		Anxiety	4				X					X					Do you avoid physical exertion because of having an aortic aneurysm?
AAA		Anxiety	3				X					X					Do you avoid travelling independently because of aortic aneurysm?
AAA		ADL	3									X					How much do problems caused by aortic aneurysm affect your overall enjoyment of life?
AAA		ADL	4				X					X					How much does aortic aneurysm affect your physical activities? (E.g. Exercise walking or going out)
AAA		ADL	3				X					X					How much does aortic aneurysm affect your ability to undertake personal roles and responsibilities? (E.g.

																	**Caring for others study or work?**
**AAA**		**ADL**	**3**									**X**					**How much do you feel aortic aneurysm affects your ability to look after yourself? (E.g. Rest wash toilet or feed yourself)**
**AAA**		**ADL**	**4**									**X**					**How much does aortic aneurysm affect your social activities? (E.g. Visiting friends or family)**
**AAA**		**ADL**	**4**				**X**					**X**					**Do you suffer from low mood because of having an aortic aneurysm?**
**LL**		**Ischaemic Pain**	**4**	+/-	+/-				**X**	**X**	**X**			**X**		**X**	**Do you experience any cramping pain in your legs or feet?**
**LL**		**Ischaemic Pain**	**4**		+/-				**X**		**X**					**X**	**Do you experience cramping pain in your legs or feet when walking?**
**LL**		**Ischaemic Pain**	**4**		+/-						**X**					**X**	**How far can you walk before you experience any cramping pain in your legs or feet?**
**LL**		**Ischaemic Pain**	**4**		+/-						**X**					**X**	**Do you walk more slowly than you would choose to in order to avoid cramping pain in your legs and feet?**
**LL**		**Ischaemic Pain**	**4**		-						**X**					**X**	**Do you experience cramping pain in your legs or feet when walking uphill?**
**LL**		**Ischaemic Pain**	**4**		-						**X**					**X**	**Do you experience pain in your legs or feet when you climb stairs?**
**LL**		**Ischaemic Pain**	**4**		-				**X**	**X**	**X**			**X**	**X**	**X**	**Do you experience pain in your feet at night?**
**LL**		**Ischaemic Pain**	**4**		-												**Do you dangle one or both of your legs over the side of the bed to help reduce foot pain?**
**LL**		**Ischaemic Pain**	**4**		-				**X**	**X**	**X**			**X**	**X**	**X**	**Do you experience severe pain in your legs or feet when you are resting or sitting?**
**LL**		**Ischaemic Pain**	**2**		-											**X**	**Are you troubled by cold feet?**
**LL**		**Ulcer**	**4**	**X**	-					**X**	**X**			**X**	**X**	**X**	**Have you ever had any ulcers on your legs or feet now or at any time in the past?**
**LL**		**Ulcer**	**4**		-					**X**	**X**			**X**	**X**	**X**	**Please use the image below to show where you currently have any leg or foot ulcers.**
**LL**		**Ulcer**	**4**	-	-					**X**					**X**	**X**	**Are you concerned about the smell of your leg ulcers?**
**LL**		**Ulcer**	**4**	**X**	-										**X**	**X**	**Are you concerned about the appearance of your leg ulcers?**
**LL**		**Ulcer**	**4**	-	-										**X**	**X**	**Do you have leg ulcers that leak fluid (watery liquid)?**
**LL**		**Ulcer**	**4**	-	-										**X**	**X**	**Do you experience infections in your leg ulcers? (E.g. foul smell or pus)**
**LL**		**Ulcer**	**4**	-	-					**X**					**X**	**X**	**Do you experience repeated leg ulcers?**
**LL**		**Ulc**	**4**	**X**	-					**X**					**X**	**X**	**Do you worry about your leg ulcers? (E.g. Not healing**

		er															becoming infected losing part of your leg or foot).
LL		VV	4		-	-								X			Do you experience any bleeding from veins in your legs or feet?
LL		VV	4		-	X			X					X			Do you have any problems with the skin over your varicose veins?
LL		VV	4		-	X			X					X			Do varicose veins make you feel self-conscious or embarrassed?
LL		VV	4	X	-	X			X					X	X	X	Do leg or foot problems affect what clothing or shoes you can wear?
LL		VV	4		-	X			X					X	X		Do you experience any swelling in your legs or feet?
LL		VV	4		-	X			X					X	X	X	Do you experience itching in your legs or feet?
LL		VV	4		-	X			X					X	X		Do you wear compression stockings or tights for your legs?
LL		Tissue loss	4		-	-				X	X					X	Have you lost any part of your legs or feet through amputation or gangrene?
LL		Tissue loss	4			-				X	X					X	Please click on the appropriate part or parts of your legs feet or toes that you have had amputated or have been lost â€¦
LL		Anxiety	3	X	X	X			X	X	X			X	X	X	Do you worry about your leg problems getting worse in the future?
LL		ADL	3	+/-	X	+/-			X	X	X			X	X	X	How much do leg or foot problems affect your overall enjoyment of life?
LL		ADL	3	+/-	X	-			X	X	X			X	X	X	How much do leg or foot problems affect your ability to carry out physical activities? (E.g. Walking housework or exercise)
LL		ADL	3	+/-	X	+/-			X	X	X			X	X	X	How much do leg or foot problems affect your personal responsibilities? E.g. caring for others study or work
LL		ADL	3	-	X	+/-			X	X	X			X	X	X	How much do leg or foot problems affect your ability to look after yourself? (E.g. Rest wash toilet or feed yourself)
LL		ADL	3	+/-	X	+/-			X	X	X			X	X	X	How much do leg or foot problems affect your social activities? (E.g. Going out visiting friends or family)
LL		ADL	4	X	X	-			X	X	X			X	X	X	Do you suffer from low mood because of leg or foot problems?
Generic		Personal data		-	X	-	X	X		X	X	X	X		X	X	Do you rely on any people to help you with your everyday activities?
Generic		Personal data		-			X		X	X	X	X			X	X	Do you experience financial problems because of your vascular condition?

Abbreviations: Abdominal aortic aneurysm (AAA), Aberdeen varicose veins questionnaire (AVVQ), Activity of daily living (ADL), Carotid artery disease (CAD), Peripheral arterial disease (PAD), , Lower limb (LL), Peripheral arterial disease (PADQOL), Venous leg ulcer (VLU), Venous leg ulcer Quality of life (VLU-QOL), Varicose veins (VV). The items of the eight scales are colored in this table.

Table 6: Structure of the main ePAQ-VAS with evidence base for inclusion of individual items, scales and sections.

Figure 2. Evidence synthesis to develop of ePAQ-VAS

Insert figure 2 here

ePAQ-VAS was presented to 19 vascular patients. Overall the response was positive; the participants felt the generic and the relevant disease specific were comprehensive,

fit for purposeand potentially useful. There was little consistency in items that participants found difficult and no individual item was identified with which most participants had difficulty.

Discussion included the use of abbreviations, font size and contrast between text and background, response options and scales, electronic format versus paper format, relevance to patients and clinicians, the use of free text boxes and the language and wording used, when and how to use the skip button, repetition of items and subject matter and the possibility of emotional distress associated with questions about the possibility of deterioration or death.

Figure 3. Overview of ePAQ-VAS structure

Insert figure 3 here

Based on the findings from the face validity exercise, and input from the Vascular PROMs group, further revisions were made in an iterative process; culminating in the development of ePAQ-VAS. The structure of the questionnaire is illustrated in Figure 3. Fifty-nine items were eliminated for overlap; these include questions asking about common symptoms experienced across most vascular conditions. Five items were added based on suggestion from clinicians. Generic items for all respondents were presented in the first section and include questions about pain, altered sensation, weakness, weight/height, smoking habit, previous medical history and regular medication. This information was deemed important for assessment of vascular conditions both by patients and clinicians.

The next three sections are condition specific relating to CAD, AAA and LL vascular disease sections. These sections are further divided into scales. There are fifty-five items within eight scales and the remainder of questions do not contribute to scales but are kept due to their clinical relevance. The eight scales are part of the of condition specific sections and include CAD related anxiety, impact of CAD on activities of daily living (ADL), AAA related anxiety, Impact of AAA on activities of ADL, PAD symptoms, VLU symptoms, VV symptoms and Impact of LL vascular

For peer review only

disease on ADL. Individual items, scales and sections of ePAQ-VAS in its initial version can be viewed on https://demo-questionnaire.epaq.co.uk/home/project?id=VASC_1.6&page=1

The evidence used to develop each item in ePAQ-VAS is made explicit in **Table 5**, this table show whether the source for the item is the qualitative study, reviews or consensus study.

Discussion

This study documents stages undertaken to develop ePAQ-VAS and the conceptual framework underpinning this new tool for use in undifferentiated vascular populations. The main strength of this new instrument is that it can be used as a holistic clinical assessment tool that can be completed by patients before meeting the vascular surgeon in the clinic. The information generated can be used to help shared decision making by focusing on patient priorities. This tool has the further advantage of being an electronic online patient reported outcome measure since it can be used to monitor impact of the disease and/or interventions overtime. Furthermore, this instrument is preference based, unlike the identified vascular PROMs **(4-8)**; once further validated the disease specific scales can be used to generate utility values either by mapping to the values of a generic utility measure, or by further utility studies **(18).**

This instrument has been developed in line with FDA guidelines for developing PROMs **(3)**. Items were developed based on themes extracted from primary qualitative data, systematic qualitative evidence synthesis and clinicians' consensus exercise **(9-13)**. We have made efforts through purposive sampling to ensure that we have included diverse demographic groupings in the primary research, and this is augmented by the inclusion of systematic reviews that include evidence gathered in national and international studies. Another strength of this study is that the qualitative evidence in the review and the primary study included patients at different stages of their disease. The data collected included the impact of disease, including symptoms, on daily living and the impact of diagnosis and treatment on the daily living. The vascular clinicians' input into developing and rating the items was sought, and new

For peer review only

items were incorporated based on recommendation from twenty-five vascular clinicians.

The work of developing individual items and their assignment to putative scales and sections was based both on the framework of existing PROMs **(4-8),** and on input from vascular clinicians. In this stage of the ePAQ-VAS development, an inclusive approach was chosen, and all relevant items were incorporated except for those with clear repetition. The main limitation of this draft version of ePAQ-VAS is that it is long and potentially repetitive; It is expected that factor analysis and psychometric testing will lead to a reduction in the number of individual items and will confirm (or refute) the putative scales identified in the current version. Furthermore, skipping rules embedded within the questionnaire will only present the items relevant to the patient completing the online instrument.

Another limitation is that ePAQ-VAS only cover the five main vascular conditions and it might not be relevant to patients with other vascular disease. However, including all vascular conditions in one instrument is not possible and the evidence to include only these conditions was based on input from clinicians treating vascular disease. As stated by the FDA **(3)** a fundamental consideration in the development of PROMs is the adequacy of item generation. Due to the heterogeneous nature of vascular disease, it was not straightforward to identify what exactly should be measured when developing and defining the initial conceptual framework for the ePAQ-VAS. To this end, as recommended by the FDA, the initial conceptual framework was based on information gathered from reviews of the literature, patients and expert opinion.

The findings of the qualitative study indicated an overlap in patient experiences of the various conditions. However, there was also a clear difference in how each condition impacted on different aspects of quality of life. There were conditions with many physical symptoms and others with none. This demonstrated that while it may be possible to develop a PROMs for use across a variety of vascular conditions, it would also need to include condition-specific items to fully capture the impact and clinically relevant information for each disease or condition. A further limitation is that the face validity study was not able to examine the comprehensiveness of ePAQ-VAS since it

For peer review only

covered multiple conditions and it was difficult to expect from any of the patient groups interviewed to comment on diseases they have not experienced. Therefore, they only commented on the generic questions and the disease specific items relating to their condition.

In conclusion ePAQ-VAS is a multi-dimensional measure developed for use in a range of vascular conditions. It is a single electronic tool, covering most vascular conditions. This is important for those patients presenting with mixed symptoms or multiple conditions. The items in ePAQ-VAS can capture information about disease symptoms, HRQoL, co-morbidities, medical history and other relevant healthcare issues. This type of information can aid communication between healthcare professionals and patients and support shared-decision making. The electronic format may make it easier to monitor patients over time, especially those with chronic conditions and those treated with lifestyle modification or conservatively. Based on methods used in its construction, this tool has a strong degree of content validity; however, further psychometric testing for reliability, responsiveness, and validity is needed. Once this electronic PROMs is validated, it can be used as an outcome measure in clinical practice and research.

Contributorship statement

JM, GJ, AA, SR designed the study. AA, PP, EL, SR, GJ performed the analysis of the data and theme generation. ST and SN helped with consensus study and qualitative study recruitment. AA wrote the paper and all authors reviewed and edited the paper. All authors approved the manuscript.

Competing interests

Stephen Radley is a shareholder in ePAQ Systems Ltd, an NHS spin-out technology company, the majority shareholder being Sheffield Teaching Hospitals. The other authors have no conflict of interest.

Funding

This manuscript presents independent research funded by the National Institute for Health Research (NIHR) under the Programme Grants for Applied Research (RP-PG-1210-12009).

For peer review only - http://bmjopen.bmj.com/site/about/guidelines.xhtml

Data sharing statement

No data are available

References:

1- Beebe-Dimmer JL, Pfeifer JR, Engle JS, Schottenfeld D. The epidemiology of chronic venous insufficiency and varicose veins. Ann Epidemiol. 2005 Mar;15(3):175-84.
2- Peach G, Griffin M, Jones KG, Thompson MM, Hinchliffe RJ. Diagnosis and management of peripheral arterial disease. BMJ. 2012 Aug 14;345:e5208.
3- Food and Drug Administration (FDA), Guidance for Industry: Patient-Reported Outcome Measures: Use in Medical Product Development to Support Labeling Claims, accessed and available at: https://www.fda.gov/downloads/drugs/guidances/ucm193282.pdf
4- Aber A, Poku E, Phillips P, Essat M, Buckley Woods H, Palfreyman S, Kaltenthaler E, Jones G, Michaels J. Systematic review of patient-reported outcome measures in patients with varicose veins. Br J Surg. 2017 Oct;104(11):1424-1432.
5- Duncan R, Essat M, Jones G, Booth A, Buckley Woods H, Poku E, Kaltenthaler E, Keetharuth AD, Palfreyman S, Michaels J. Systematic review and qualitative evidence synthesis of patient-reported outcome measures for abdominal aortic aneurysm. Br J Surg. 2017 Mar;104(4):317-327. doi: 10.1002/bjs.
6- Poku E, Duncan R, Keetharuth A, Essat M, Phillips P, Woods HB, Palfreyman S, Jones G, Kaltenthaler E, Michaels J. Patient-reported outcome measures in patients with peripheral arterial disease: a systematic review of psychometric properties. Health Qual Life Outcomes. 2016 Nov 24;14(1):161.
7- Poku E, Aber A, Phillips P, Essat M, Buckley Woods H, Palfreyman S, Kaltenthaler E, Jones G, Michaels J. Systematic review assessing the measurement properties of patient-reported outcomes for venous leg ulcers. 2017 Oct;1(5):138-147.
8- Essat M, Aber A, Phillips P, Poku E, Woods HB, Howard A, Palfreyman S, Kaltenthaler E, Jones G, Michaels J. Patient-Reported Outcome Measures in Carotid Artery Revascularization: Systematic Review and Psychometric Analysis. Ann Vasc Surg. 2018 Jul;50:275-283.
9- Aber A, Lumley E, Phillips P, Woods HB, Jones G, Michaels J. Themes that Determine Quality of Life in Patients with Peripheral Arterial Disease: A Systematic Review. Patient. 2018 May 8. doi: 10.1007/s40271-018-0307-7.
10- Phillips P, Lumley E, Duncan R, Aber A, Woods HB, Jones GL, Michaels J. A systematic review of qualitative research into people's experiences of living with venous leg ulcers. J Adv Nurs. 2018 Mar;74(3):550-563.
11- Aber A, Howard A, Woods HB, Jones G, Michaels J. Impact of Carotid Artery Stenosis on Quality of Life: A Systematic Review. Patient. 2018 Oct 17. doi: 10.1007/s40271-018-0337-1.
12- Lumley E, Phillips P, Aber A, Woods HB, Jones G, Michaels J. Experiences of living with varicose veins: a systematic review of qualitative research. J Clin Nurs. 2018 Nov. J Clin Nurs. 2018 Nov 21. doi: 10.1111/jocn.14720.
13- Duncan R, Lumley E, Tod A, Hughes J, Palfreyman S, Nawaz S, Jones G, Michaels J. The experiences of people living with a vascular condition: a qualitative study. HEDS Discussion Papers, University of Sheffield. 2018 Jul. https://www.sheffield.ac.uk/scharr/sections/heds/discussion-papers/18_07-1.794000 (Accessed 19 November 2018).
14- Mokkink LB, Terwee CB, Patrick DL, Alonso J, Stratford PW, Knol DL et al. The COSMIN study reached international consensus on taxonomy, terminology, and definitions of measurement properties for health-related patient-reported outcomes. J Clin Epidemiol 2010; 63: 737–745.
15- Ritchie J, Spencer L. Qualitative data analysis for applied policy research. In: Bryman G, Burgess A, editors. Analysing qualitative data. London: Routledge; 1994. p. 173–94.
16- Hsu, C., & Sandford, B. (2007). The Delphi Technique : Making Sense Of Consensus. Practical Assessment, Research & Evaluation, 12(10). http://www.endoedu.com/mobile/guideline/delphi.pdf (Accessed 19 November 2018).
17- Garratt AM, Macdonald LM, Ruta DA, Russell IT, Buckingham JK, Krukowski ZH. Towards measurement of outcome for patients with varicose veins. Qual Health Care 1993; 2: 5–10
18- Brazier J, Deverill M. A checklist for judging preference-based measures of health related quality of life: learning from psychometrics. Health Econ 1999; 8: 41–5

For peer review only

For peer review only

Figure 1. Development of ePAQ-VAS conceptual framework

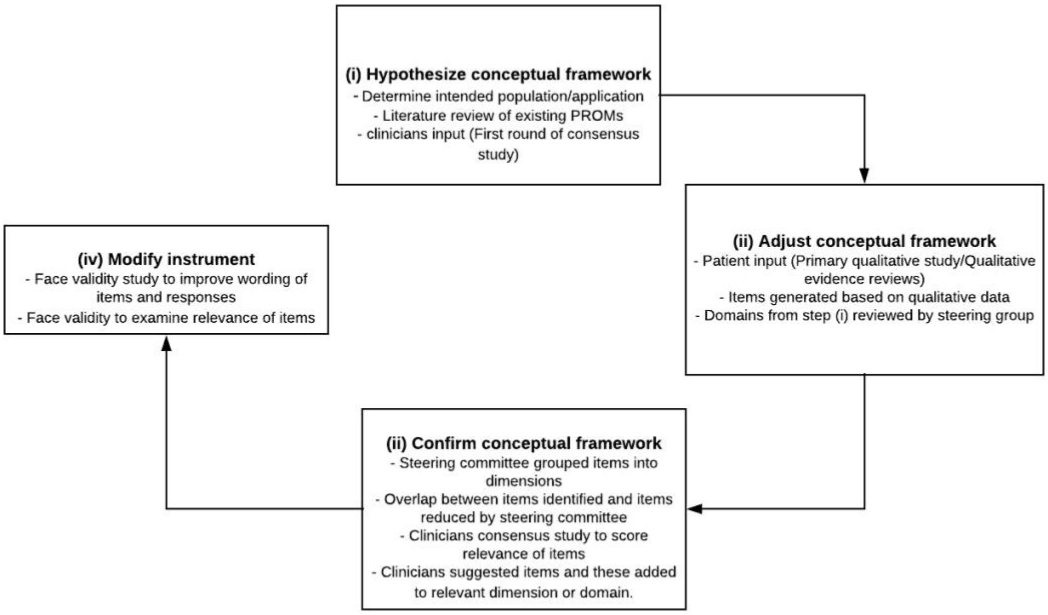

Figure 2. Evidence synthesis to develop of ePAQ-VAS

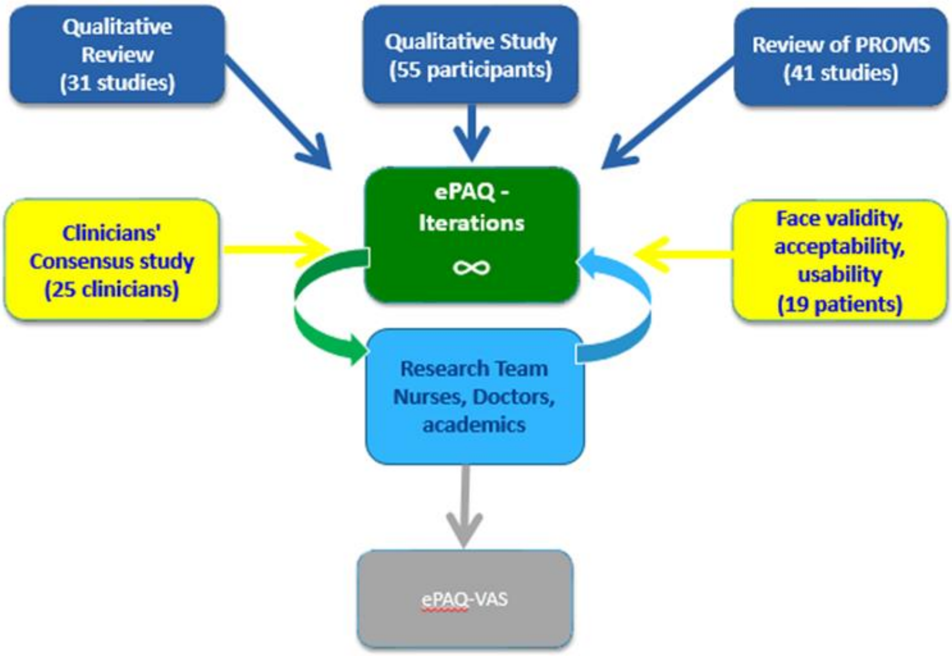

For peer review only

Figure 3. Overview of ePAQ-VAS structure

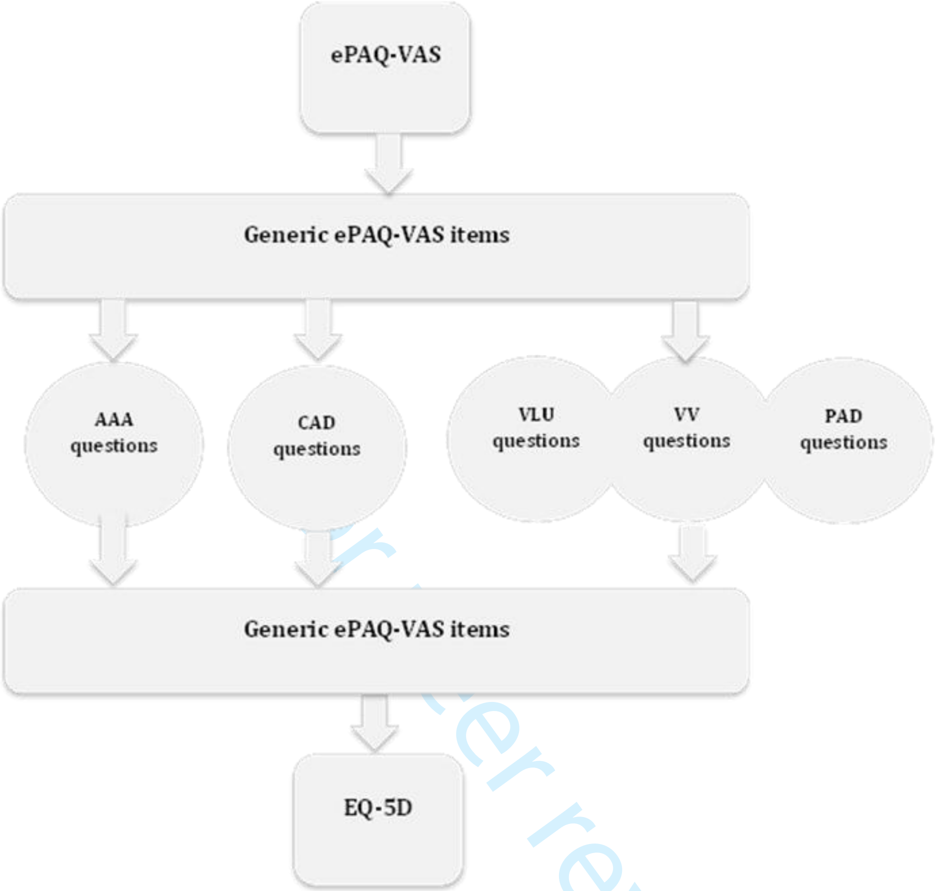

Supplementary material

S 1

Table 1- Appraisal criteria for assessing the psychometric properties of patient-reported outcome measures

Psychometric property domains	Subdomain	Thresholds
Reliability	Test–retest reliability	Intraclass correlation/weighted κ ≥ 0·70 for group comparisons
		Intraclass correlation/weighted κ ≥ 0·90 for individual scores
		Evidence of mean difference between time point 1 and time point 2, reported with 95 per cent c.i. (using paired *t* test or Wilcoxon signed-rank test)
	Internal consistency	Cronbach's α score of ≥ 0·70 indicates good evidence, but the score should not exceed ≥ 0·92 for group comparison
		Item total correlations should be ≥ 0·20
Validity	Content validity	Evidence that the instrument has been developed by undertaking a literature review, consulting patients, clinicians and other experts

For peer review only

Psychometric property domains	Subdomain	Thresholds
	Construct validity	Correlation coefficient of ≥ 0·60 indicates strong evidence<br>This should be supported by specific directional hypotheses and a previous estimation of strength of correlation.
	Criterion validity	Justification for selection of standard should be adequate<br>Correlation between PROM and standard ≥ 0·70
Responsiveness	Responsiveness	Statistically significant changes in score of an expected magnitude based on methods including *t* tests, effect size, standardized response means, Guyatt's responsiveness index or responsiveness statistics
Acceptability	Floor/ceiling effects	Evidence of floor effect: 15 per cent of respondents are achieving the lowest score on the instrument<br>Evidence of ceiling effect: 15 per cent of respondents are achieving the highest score on the instrument
	Acceptability	Completeness of data supplied ≥ 80 per cent

For peer review only

S 2

AAA search strategies: search one
Database: Ovid MEDLINE(R) In-Process & Other Non-Indexed Citations and Ovid MEDLINE(R) <1946 to Present>
Search Strategy:
----
1. (Aortic aneurysm$ or triple A or true aneurysm$).tw.
2. Aortic Aneurysm/
3. Aortic Aneurysm, Abdominal/
4. 1 or 2 or 3
5. (patient reported outcome$ or patient-reported outcome$).tw.
6. (prom or proms).tw.
7. (disease reported outcome$ or disease-reported outcome$).tw.
8. 5 or 6 or 7
9. "Quality of Life"/
10. "Outcome and Process Assessment (Health Care)"/
11. "Outcome Assessment (Health Care)"/
12. quality of life.tw.
13. qol.tw.
14. outcome measure$.tw.
15. health outcome$.tw.
16. or/9-15
17. (patient adj20 report$).tw.
18. 16 and 17
19. 8 or 18
20. nottingham health profile.tw.
21. health related quality of life.tw.
22. health related qol.tw.
23. health related ql.tw.
24. hrqol.tw.
25. hql.tw.
26. health state utilit$.tw.
27. hsuv$.tw.

For peer review only

28. (euroqol or euro qol or eq5d or eq 5d).tw.
29. (sf6d or sf 6d or sf 6 dimension$ or sf six dimension$ or shortform 6d or shortform six dimension$ or short form 6d or short form 6 dimension$ or short form six dimension$).tw.
30. (sf12 or sf 12 or short form 12 or shortform 12 or sf twelve or sftwelve or shortform twelve or short form twelve).tw.
31. (sf36 or sf 36 or short form 36 or shortform 36 or sf thirtysix or sf thirty six or shortform thirtysix or shortform thirty six or short form thirtysix or short form thirty six).tw.
32. (item adj3 short form).tw.
33. (item adj3 shortform).tw.
34. medical outcomes survey.tw.
35. medical outcomes study.tw.
36. mos.tw.
37. psychological general wellbeing index.tw.
38. psychological general well being index.tw.
39. pgwb$.tw.
40. health utilit$.tw.
41. hui$.tw.
42. quality of wellbeing.tw.
43. quality of well being.tw.
44. qwb$.tw.
45. rosser.tw.
46. trade off$.tw.
47. standard gamble$.tw.
48. tto$.tw.
49. qaly$.tw.
50. quality adjusted life year$.tw.
51. quality-adjusted life years/
52. hye$.tw.
53. health$ year$ equivalent$.tw.
54. disutilit$.tw.
55. disbenefit$.tw.
56. "Quality of Life"/
57. "Outcome Assessment (Health Care)"/
58. quality of life.tw.
59. 56 or 57 or 58
60. (preference based or utilit$ or generic preference).tw.
61. 59 and 60
62. (preference$ adj2 (elicit$ or patient$ or population$ or measure$ or based or cost$)).tw.
63. (utilit$ adj2 (elicit$ or patient$ or population$ or measure$ or based or cost$)).tw.
64. or/20-55

For peer review only

65. 61 or 62 or 63 or 64
66. 19 or 65
67. 4 and 66

AAA search strategies: search two
Database: Ovid MEDLINE(R) In-Process & Other Non-Indexed Citations and Ovid MEDLINE(R) <1946 to Present>
Search Strategy:
----
1. (Aortic aneurysm$ or triple A or true aneurysm$).tw.
2. Aortic Aneurysm/
3. Aortic Aneurysm, Abdominal/
4. or/1-3
5. time trade off.mp.
6. Watt index.mp.
7. (Rand-36 or Rand36 or Rand 36).mp.
8. self-assessed health.mp.
9. screenQL.mp.
10. general health questionnaire.mp.
11. female sexual function index.mp.
12. Rose questionnaire.mp.
13. Australian Vascular Quality of Life Index.mp.
14. AUSVIQUOL.mp.
15. Nottingham Health Profile.mp.
16. NHP.mp.
17. (sf36 or sf 36 or short form 36 or shortform 36 or sf thirtysix or sf thirty six or shortform thirtysix or shortform thirty six or short form thirtysix or short form thirty six).mp. [mp=title, abstract, original title, name of substance word, subject heading word, keyword heading word, protocol supplementary concept word, rare disease supplementary concept word, unique identifier]
18. (sf12 or sf 12 or short form 12 or shortform 12 or sf twelve or sftwelve or shortform twelve or short form twelve).mp. [mp=title, abstract, original title, name of substance word, subject heading word, keyword heading word, protocol supplementary concept word, rare disease supplementary concept word, unique identifier]
19. (sf20 or sf 20 or short form 20 or shortform 20 or sf twenty or sftwenty or shortform twenty or short form twenty).mp. [mp=title, abstract, original title, name of substance word, subject heading word, keyword heading word, protocol supplementary concept word, rare disease supplementary concept word, unique identifier]
20. (Hospital Anxiety and Depression Scale).mp. [mp=title, abstract, original title, name of substance word, subject heading word, keyword heading word, protocol supplementary concept word, rare disease supplementary concept word, unique identifier]
21. HADS.mp.
22. Standard gamble$.mp. [mp=title, abstract, original title, name of substance word, subject heading word, keyword heading word, protocol supplementary concept word, rare disease supplementary concept word, unique identifier]
23. rosser.tw.
24. (euroqol or euro qol or eq5d or eq 5d or eq-5d or eq-5d VAS).mp. [mp=title, abstract, original title, name of substance word, subject heading word, keyword heading word, protocol supplementary concept word, rare disease supplementary concept word, unique identifier]

For peer review only

25. World Health Organization Quality of Life-BREF.mp. [mp=title, abstract, original title, name of substance word, subject heading word, keyword heading word, protocol supplementary concept word, rare disease supplementary concept word, unique identifier]
26. WHOQOL-BREF.mp. [mp=title, abstract, original title, name of substance word, subject heading word, keyword heading word, protocol supplementary concept word, rare disease supplementary concept word, unique identifier]
27. or/5-26
28. instrumentation.sh.
29. methods.sh.
30. Validation Studies.pt.
31. Comparative Study.pt.
32. Psychometrics/
33. psychometr*.ti,ab.
34. clinimetr*.tw.
35. clinometr*.tw.
36. "Outcome Assessment (Health Care)"/
37. outcome assessment.ti,ab.
38. outcome measure*.tw.
39. Observer Variation/
40. observer variation.ti,ab.
41. Health Status Indicators/
42. "Reproducibility of Results"/
43. reproducib*.ti,ab.
44. Discriminant Analysis/
45. reliab*.ti,ab.
46. unreliab*.ti,ab.
47. valid*.ti,ab.
48. coefficient.ti,ab.
49. homogeneity.ti,ab.
50. homogeneous.ti,ab.
51. "internal consistency".ti,ab.
52. 28 or 29 or 30 or 31 or 32 or 33 or 34 or 35 or 36 or 37 or 38 or 39 or 40 or 41 or 42 or 43 or 44 or 45 or 46 or 47 or 48 or 49 or 50 or 51
53. cronbach*.ti,ab.
54. (alpha or alphas).ti,ab.
55. 53 and 54
56. item.ti,ab.
57. (correlation* or selection* or reduction*).ti,ab.
58. 56 and 57
59. (agreement or precision or imprecision or "precise values" or "test–retest").ti,ab.
60. (test and retest).ti,ab.
61. reliab*.ti,ab.

For peer review only

62. (test or retest).ti,ab.
63. 61 and 62
64. 55 or 58 or 59 or 60 or 63
65. (stability or interrater or inter-rater or intrarater or intra-rater or intertester or inter-tester or intratester or intra-tester or interobserver or inter-observer or intraobserver or intra-observer).ti,ab.
66. (intertechnician or inter-technician or intratechnician or intra-technician or interexaminer or inter-examiner or intraexaminer or intra-examiner or interassay or inter-assay or intraassay or intra-assay or interindividual or inter-individual or intraindividual or intra-individual or interparticipant or inter-participant or intraparticipant or intra-participant).ti,ab.
67. (kappa or "kappa's" or kappas or repeatab*).ti,ab.
68. repeatab*.ti,ab.
69. 65 or 66 or 67 or 68
70. (replicab* or repeated).ti,ab.
71. (measure or measures or findings or result or results or test or tests).ti,ab.
72. 70 and 71
73. (generaliza* or generalisa* or concordance).ti,ab.
74. (intraclass and correlation*).ti,ab.
75. (discriminative or "known group" or factor analysis or factor analyses or dimension* or subscale*).ti,ab.
76. (multitrait and scaling and (analysis or analyses)).ti,ab.
77. 72 or 73 or 74 or 75 or 76
78. (item discriminant or interscale correlation* or error or errors or "individual variability").ti,ab.
79. (variability and (analysis or values)).ti,ab.
80. (uncertainty and (measurement or measuring)).ti,ab.
81. ("standard error of measurement" or sensitiv* or responsive*).ti,ab.
82. ((minimal or minimally or clinical or clinically) and (important or significant or detectable) and (change or difference)).ti,ab.
83. (small* and (real or detectable) and (change or difference)).ti,ab.
84. (meaningful change or "ceiling effect" or "floor effect" or "Item response model" or IRT or Rasch or "Differential item functioning" or DIF or "computer adaptive testing" or "item bank" or "cross-cultural equivalence").ti,ab.
85. 78 or 79 or 80 or 81 or 82 or 83 or 84
86. 52 or 64 or 69 or 77 or 85
87. 4 and 27 and 86

Databases and research registers searched:

- MEDLINE and MEDLINE In Process: Ovid.
- EMBASE: Ovid.
- Cochrane Library
    - Cochrane Database of Systematic Reviews (CDSR)
    - Database of Abstracts of Reviews of Effects (DARE)
    - Cochrane Central Register of Controlled Trials (CCRCT)

For peer review only

- Health Technology Assessment Database (HTA)
- NHS Economic Evaluation Database (NHS EED)

- CINAHL
- PROQOLID
- PsychINFO
- Science Citation Index Expanded (SCIE): Web of Science

Varicose veins systematic review search strategy

1 ((Venous or vein) adj2 (disease or stasis or obstruction or incompetence or insufficiency or syndrome or thromboembolism)).tw. (25175)
2 (Venous ulcer$ or leg ulcer$ or venous leg ulcer$).tw. (6632)
3 (Venous insufficiency or Venous incompetence or Venous thromboembolism or VTE or Venous outflow obstruction or VOO or Venous valvular incompetence or VVI).tw. (19687)
4 exp Venous Insufficiency/ (6167)
5 exp Varicose Veins/ (15946)
6 (Venous adj2 (edema or oedema)).tw. (262)
7 Venous disorder$ of the leg$.tw. (16)
8 (Varicose vein$ or spider vein$ or varicosity or varices or telangiectasia$).tw. (28086)
9 Endovenous occlusion.tw. (11)
10 post-thrombotic syndrome.tw. (716)
11 Venous Thrombosis/ or Venous Thromboembolism/ (24327)
12 or/1-11 (82600)
13 (patient reported outcome$ or patient-reported outcome$).tw. (5201)
14 (prom or proms).tw. (1755)
15 (disease reported outcome$ or disease-reported outcome$).tw. (2)
16 13 or 14 or 15 (6667)
17 "Quality of Life"/ (129051)
18 "Outcome and Process Assessment (Health Care)"/ (22708)
19 "Outcome Assessment (Health Care)"/ (52618)
20 quality of life.tw. (169753)
21 qol.tw. (22367)
22 outcome measure$.tw. (161039)
23 health outcome$.tw. (26145)
24 ((evalua$ or assess$ or measure$) adj (function$ or symptom$)).tw. (8832)
25 or/17-24 (451890)
26 (patient adj20 report$).tw. (188387)
27 25 and 26 (14509)
28 16 or 27 (17478)

For peer review only

29 (nottingham health profile or aberdeen varicose vein questionnaire or AVVQ or chronic venous insufficiency questionnaire or CIVIQ or charing cross venous ulceration questionnaire or CXVUQ or Venous Insufficiency Epidemiological Economic Study tool or VEINES-QOL or VEINES-SYM or Venous Clinical Severity Score or VCSS or Deep Vein Thrombosis Leg Symptom Index or DVT-LSI or Ferrans Powers Quality of Life Index or QLI).tw. (1447)
30 health related quality of life.tw. (25867)
31 health related qol.tw. (978)
32 health related ql.tw. (5)
33 hrqol.tw. (8922)
34 hql.tw. (87)
35 health state utilit$.tw. (334)
36 hsuv$.tw. (35)
37 (activities of daily living or ADL).tw. (19652)
38 "Activities of Daily Living"/ (52765)
39 (euroqol or euro qol or eq5d or eq 5d).tw. (5064)
40 (sf8 or sf 8 or short form 8 or shortform 8 or sf eight or shortform eight or short form eight).tw. (310)
41 (sf6d or sf 6d or sf 6 dimension$ or sf six dimension$ or shortform 6d or shortform six dimension$ or short form 6d or short form 6 dimension$ or short form six dimension$).tw. (524)
42 (sf12 or sf 12 or short form 12 or shortform 12 or sf twelve or sftwelve or shortform twelve or short form twelve).tw. (3332)
43 (sf36 or sf 36 or short form 36 or shortform 36 or sf thirtysix or sf thirty six or shortform thirtysix or shortform thirty six or short form thirtysix or short form thirty six).tw. (18047)
44 (item adj3 short form).tw. (3935)
45 (item adj3 shortform).tw. (1)
46 medical outcomes survey.tw. (240)
47 medical outcomes study.tw. (3459)
48 mos.tw. (5442)
49 psychological general wellbeing index.tw. (10)
50 psychological general well being index.tw. (203)
51 pgwb$.tw. (252)
52 health utilit$.tw. (1278)
53 hui$.tw. (2939)
54 quality of wellbeing.tw. (8)
55 quality of well being.tw. (354)
56 qwb$.tw. (221)
57 rosser.tw. (74)
58 trade off$.tw. (12716)
59 standard gamble$.tw. (732)
60 tto$.tw. (1143)
61 qaly$.tw. (6062)
62 quality adjusted life year$.tw. (7209)
63 quality-adjusted life years/ (7816)

For peer review only

64 hye$.tw. (753)
65 health$ year$ equivalent$.tw. (39)
66 disutilit$.tw. (274)
67 disbenefit$.tw. (24)
68 "Quality of Life"/ (129051)
69 "Outcome Assessment (Health Care)"/ (52618)
70 quality of life.tw. (169753)
71 68 or 69 or 70 (262013)
72 (preference based or utilit$ or generic preference).tw. (136252)
73 71 and 72 (7974)
74 (preference$ adj2 (elicit$ or patient$ or population$ or measure$ or based or cost$)).tw. (11249)
75 (utilit$ adj2 (elicit$ or patient$ or population$ or measure$ or based or cost$)).tw. (5889)
76 or/29-67 (136441)
77 73 or 74 or 75 or 76 (150495)
78 28 or 77 (164078)
79 12 and 78 (993)

S3

<u>AAA qualitative literature review</u>
Database: Ovid MEDLINE(R) In-Process & Other Non-Indexed Citations and Ovid MEDLINE(R) <1946 to Present>
Search Strategy:
----
1 (Aortic aneurysm$ or triple A or true aneurysm$).tw.
2 Aortic Aneurysm/
3 Aortic Aneurysm, Abdominal/
4 or/1-3 (38292)
5 *Attitude to Health/
6 *Self Care/
7 *Health Knowledge, Attitudes, Practice/
8 (patient* adj4 (feeling* or emotion* or view* or symptom* or perception* or attribute*)).ti,ab.
9 ("health related quality of life" or "health related qol" or "health related ql" or hrqol or hql or "patient reported outcome*" or "patient-reported outcome*" or prom or proms or "disease reported outcome*").ti,ab.
10 ("quality of life" or "qol" or "outcome measure*" or "health outcome*").ti,ab.
11 *"Quality of Life"/
12 *"Outcome Assessment (Health Care)"/ or *"Outcome and Process Assessment (Health Care)"/
13 5 or 6 or 7 or 8 or 9 or 10 or 11 or 12
14 (qualitative* or findings or interview*).mp.

For peer review only

15 focus groups/ or interviews as topic/
16 exp qualitative research/
17 14 or 15 or 16
18 14 or 15 or 16
19 4 and 13
20 17 and 19

****

Databases and research registers which were searched:

- MEDLINE and MEDLINE In Process: Ovid.
- EMBASE: Ovid.
- CINAHL: EBSCO
- PROQOLID
- PsychINFO
- Science Citation Index Expanded (SCIE): Web of Science
- Social Science Citation Index
- Proquest dissertation and theses

For peer review only

S4

Table 1: Example of triangulation between PAD PROM review, qualitative evidence synthesis and qualitative interview themes

Qualitative Review Themes	Qualitative Interview Themes	EQ-5D	NHP	SF-6D	SF-8	SF-36	AUSVI QoL	ICQ	PADQ OL	SIPic	WIQ	Vascu QOL
PAD Health Outcomes												
**Pain - location**	Positive	+/-	-	-	-	-	+	-	+	+/-	-	+
**Pain - sensation**	Positive	+/-	+	+	+	+	-	+	+	-	+	-
**Pain - severity**	Positive	+	-	-	-	-	-	-	+/-	+	-	+
**Pain on walking**	Positive	+	+	-	-	-	+	+	+	+	+	+
**Pain at rest**	Positive	+	+	-	-	-	+	+	+	-	-	+
**Mobility - walking ability**	Postive	+/-	+	-	-	+	+	+	+	+	+	+
**Mobility - walking distance**	Positive	+/-	+/-	-	-	+	+	+	-	+	+	+
**Mobility - walking speed**	Positive	+/-	-	-	-	-	-	-	-	-	+	-
**Mobility - stair climbing**	Positive	+/-	+/-	-	-	+	-	+	-	+	+	-

For peer review only

**Mobility - walking uphill**	Positive	+/-	-	-	-	-	-	-	-	-	-	-
**Bending/ picking up things**	Negative	+/-	-	-	-	+	-	-	-	-	-	-
**Sleep**	Positive	-	+	-	-	+	+	-	-	-	-	--
**Sleep/ pain**	Silence	-	+	-	-	-	+	-	-	-	-	-
**Fatigue**	Negative	-	+	-	-	+	-	-	-	-	-	+
**Sexual functioning**	Negative	-	+	-	-	-	-	-	-	-	-	-
**Comorbiditi es**	Negative	-	-	-	-	-	-	-	-	-	-	-
**Non-healing wounds**	Positive	-	-	-	-	-	+	-	-	-	-	-
**Symptom progression**	Postive	-	-	-	-	-	-	-	-	-	-	-
**Appearance of leg**	Silence	-	-	-	-	-	+	-	-	-	-	-
**Altered sensations in leg**	Silence	-	-	-	-	-	-	-	-	-	-	-
**Foot temperature**	Silence	-	-	-	-	-	-	-	-	-	-	-
Impact on Physical Functioning												
**Personal care**	Positive	+	+	+/-	+/-	+	+	-	+	+	-	+
**Hobbies**	Positive	+	-	-	-	+/-	-	+	+	+	-	+/-
**Exercise**	Negative	+/-	-	-	-	+/-	-	-	-	-	-	+/-

For peer review only

**Daily activities**	Positive	+/-	+	+	+	+	+	+	+	+	-	+
Impact on Social Functioning												
**Social support**	Positive	-	+	-	-	-	-	-	-	-	-	-
**Social activities**	Positive	-	+	+	+	+	+	+	+	+	-	+
**Role**	Positive	-	+/-	+	+	-	-	+	+	-	-	+/-
**Isolation**	Positive	-	+	+/-	+/-	-	+	-	+	-	-	-
**Independence/ burden**	Positive	-	+	+	-	+/-	-	+	-	-	-	+
Impact on Psychological Function												
**Loss/ grief**	Positive	-	-	-	-	+/-	+	-	+	-	-	
**Unfulfilled desire**	Negative	-	+	-	-	-	-	-	+	-	-	
**Illness beliefs**	Positive	-	+	-	-	-	-	-	+	-	-	
**Feeling old**	Positive	-	-	-	-	-	-	-	-	-	-	
**Feeling abnormal**	Positive	-	-	-	-	-	-	-	+	-	-	
**Emotions/ mood**	Positive	-	+/-	+	+	+	+	+	+	+	-	
**Depression**	Positive	-	+	+	+	+	+	+	+	-	-	
**Fears**	Positive	-	-	-	-	+	+	+	+	-	-	
**Concentration/ intellectual**		-	-	-	-	-	-	-	+	-	-	

For peer review only

**capacity**												
**Treatment beliefs**		-	-	-	-	-		-	+	-	-	
**Uncertainty over the future**	Partial agreement	-	-	-	-	-	-	+/-	+	-	-	
Key:<br>. = silence<br>-/+ = partial agreement<br>- = dissonance<br>+ = agreement												

For peer review only

S5

AAA - Symptom anxiety	—
AAA - Size increase anxiety	—
Rupture	—
Lifting	—
AAA - Anxiety Independent travel	—
AAA - ADL Overall impact	—
AAA - ADL Physical activities	—
AAA - ADL Personal responsibilities	—
AAA - ADL Independent living	—
AAA - ADL Social activity	—
AAA - ADL Low mood	—

ePAQ VASC 1.7   Minnie Mouse   Completed on 27/11/2019

Name	Minnie Mouse	Date of birth	21/09/1951	NHS Number	111 222 TEST (02)
Clinician	Mr PAD Surgeon	Clinic	Claudication Clinic	Hospital Number	666555

Lower Limb

Lower limb - Vascular disease	Yes	Lower limb - Vascular disease date	Over 5 years ago
Lower limb - Precise diagnosis date			
Lower limb - Treatments	Yes		
Lower limb - Procedures	Bypass graft of a blockage , Balloon stretch of a vessel ,	LL - Other procedures	—
Lower limb - Cramping pain	0 1 **2** 3	Impact	0 **1** 2 3
Lower limb - Walking	Most of the time		
Lower limb - Walking distance	0 1 2 **3**		
Lower limb - Walking speed	0 1 **2** 3		
Lower limb - Walking uphill	0 1 **2** 3		
Lower limb - Climbing stairs	0 1 **2** 3		
Lower limb - Pain at night	Never	Impact	—
Lower limb - Dangling	—		
Lower limb - pain resting	—		
Lower limb - Leg ulcers	—	Impact	—
Lower limb - Ulceration	Yes	Lower limb - Ulceration first notice	3 - 5 years
LL - Ulceration location	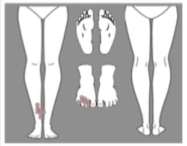		
LL - Ulceration smell	**0** 1 2 3		
LL - Ulceration appearance	**0** 1 2 3		
LL - Ulceration fluid leakage	**0** 1 2 3		
LL - Ulceration infection	**0** 1 2 3		
LL - Ulceration recurrence	No		
LL - Ulceration healing anxiety	**0** 1 2 3		

©ePAQ systems Ltd 2020

ePAQ VASC 1.7   Minnie Mouse   Completed on 27/11/2019

Name	Minnie Mouse	Date of birth	21/09/1951	NHS Number	111 222 TEST (02)
Clinician	Mr PAD Surgeon	Clinic	Claudication Clinic	Hospital Number	666555

Carotid Artery Disease

CAD Diagnosis	No	Diagnosis time frame	—
CAD - Diagnosis date			
CAD - Previous TIA	—	CAD - TIA Count	—
CAD - Most recent TIA			
CAD - First TIA			
CAD - Previous Stroke	—	CAD - Stroke Count	—
CAD - Most recent stroke			
CAD - First stroke			
CAD - TIA/Stroke Anxiety	—		
CAD - Anxiety	—		
Health anxiety	—		
Independence anxiety	—		
CAD - Balance	—		
CAD - Memory symptoms	—		
CAD - Altered speech	—		
CAD - Swallowing	—		
CAD - Vision	—		
Right eye symptoms	—		
CAD - Left eye	—		
CAD - ADL overall impact	—		
CAD - Physical activity	—		
CAD - Personal responsibilities	—		
CAD - Independent living	—		
CAD - Social activity	—		

©ePAQ systems Ltd 2020

Frequency of pain	Occasionally	Pain - length of time	Less than 6 months
Pain - ADL Overall impact	0 1 2 3		
Sensation change	Yes	Cause	Numb right below knee stump
Sensation change - Location	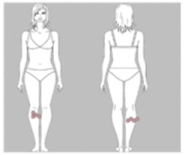		
Sensation change - Description	Stump of right leg has always been numb since my operation		
Sensation change - Frequency	All of the time	Sensation change - Duration	2 or more years
Sensation change - ADL Overall impact	0 1 2 3		
Weakness	No	Weakness - Cause	##QuestionShown##
Weakness location	—		
Weakness - Description	—		
Weakness - Frequency	—	Weakness - Duration	—
Weakness - ADL Overall impact	—		
BMI	Height:157.48cm Weight:60.33kg BMI: 24		
Smoking status	Ex-smoker (quit more than 1 year ago)	Current habit	
Smoking history	30		
Smoking history cont.	15		
Smoking habit	;		
Diabetes status	Yes	Diabetes duration	3-5 years
Diabetes treatment	Diet , Tablets ,		
Blood clots	None ,		
Medical treatments	Aspirin , Cholesterol lowering (Statin)		

ePAQ VASC 1.7 Minnie Mouse Completed on 27/11/2019

Name	Minnie Mouse	Date of birth	21/09/1951	NHS Number	111 222 TEST

Anxiety		Dimension skipped	
ADL		Dimension skipped	
CNS Symptoms		Dimension skipped	
**Aortic aneurysm**		**Score (0 - 100)**	**Impact**
Quality of Life		Dimension skipped	
Anxiety		Dimension skipped	
AAA Symptoms		Insufficient data	
**Lower Limb**		**Score (0 - 100)**	**Impact**
Pain	0		
Ulceration	0		
Varicose veins		Insufficient data	
ADL	35		
Claudication	62		

ePAQ VASC 1.7 Minnie Mouse Completed on 27/11/2019

Name	Minnie Mouse	Date of birth	21/09/1951	NHS Number	111 222 TEST (02)
Clinician	Mr PAD Surgeon	Clinic	Claudication Clinic	Hospital Number	666555

Generic

Consent	Yes		
Pain	Yes	Cause of pain	Poor circulation in my left leg

r review only

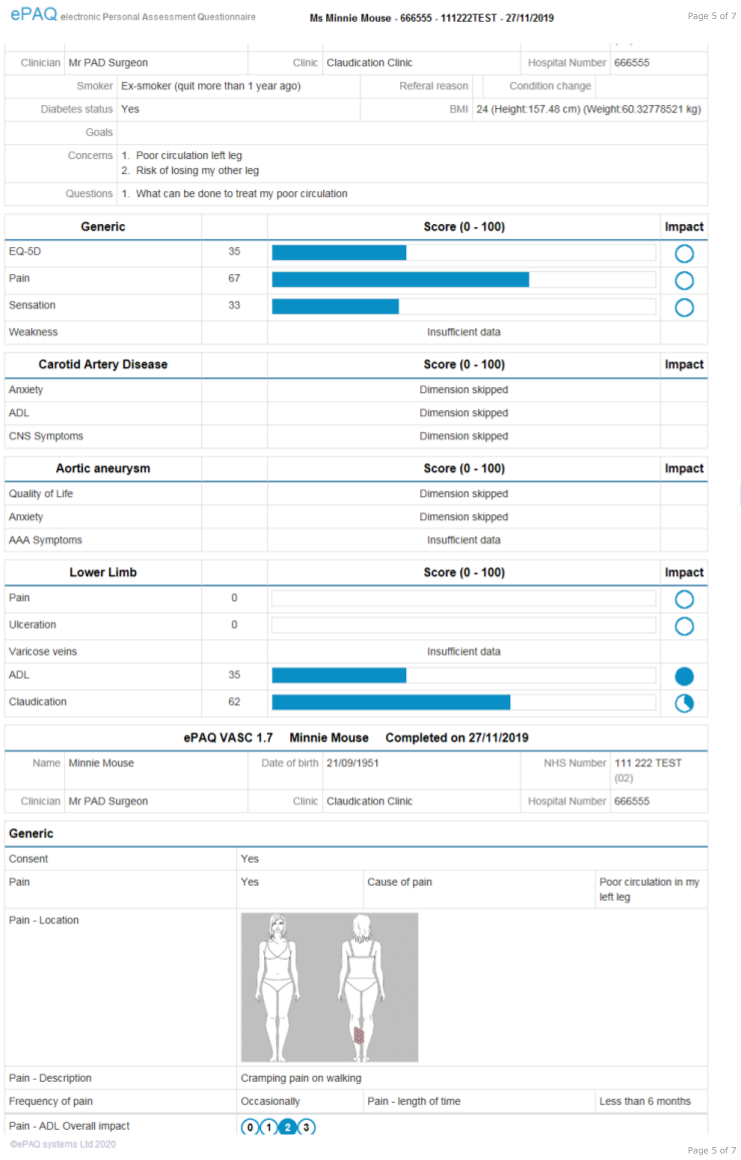

ePAQ electronic Personal Assessment Questionnaire — Ms Minnie Mouse - 666555 - 111222TEST - 27/11/2019 — Page 5 of 7

Clinician	Mr PAD Surgeon	Clinic	Claudication Clinic	Hospital Number	666555
Smoker	Ex-smoker (quit more than 1 year ago)	Referal reason		Condition change	
Diabetes status	Yes	BMI	24 (Height:157.48 cm) (Weight:60.32778521 kg)		
Goals					
Concerns	1. Poor circulation left leg<br>2. Risk of losing my other leg				
Questions	1. What can be done to treat my poor circulation				

Generic		Score (0 - 100)	Impact
EQ-5D	35		
Pain	67		
Sensation	33		
Weakness		Insufficient data	

Carotid Artery Disease		Score (0 - 100)	Impact
Anxiety		Dimension skipped	
ADL		Dimension skipped	
CNS Symptoms		Dimension skipped	

Aortic aneurysm		Score (0 - 100)	Impact
Quality of Life		Dimension skipped	
Anxiety		Dimension skipped	
AAA Symptoms		Insufficient data	

Lower Limb		Score (0 - 100)	Impact
Pain	0		
Ulceration	0		
Varicose veins		Insufficient data	
ADL	35		
Claudication	62		

ePAQ VASC 1.7 Minnie Mouse Completed on 27/11/2019

Name	Minnie Mouse	Date of birth	21/09/1951	NHS Number	111 222 TEST (02)
Clinician	Mr PAD Surgeon	Clinic	Claudication Clinic	Hospital Number	666555

Generic			
Consent	Yes		
Pain	Yes	Cause of pain	Poor circulation in my left leg
Pain - Location			
Pain - Description	Cramping pain on walking		
Frequency of pain	Occasionally	Pain - length of time	Less than 6 months
Pain - ADL Overall impact	0 1 2 3		

©ePAQ systems Ltd 2020 — Page 5 of 7

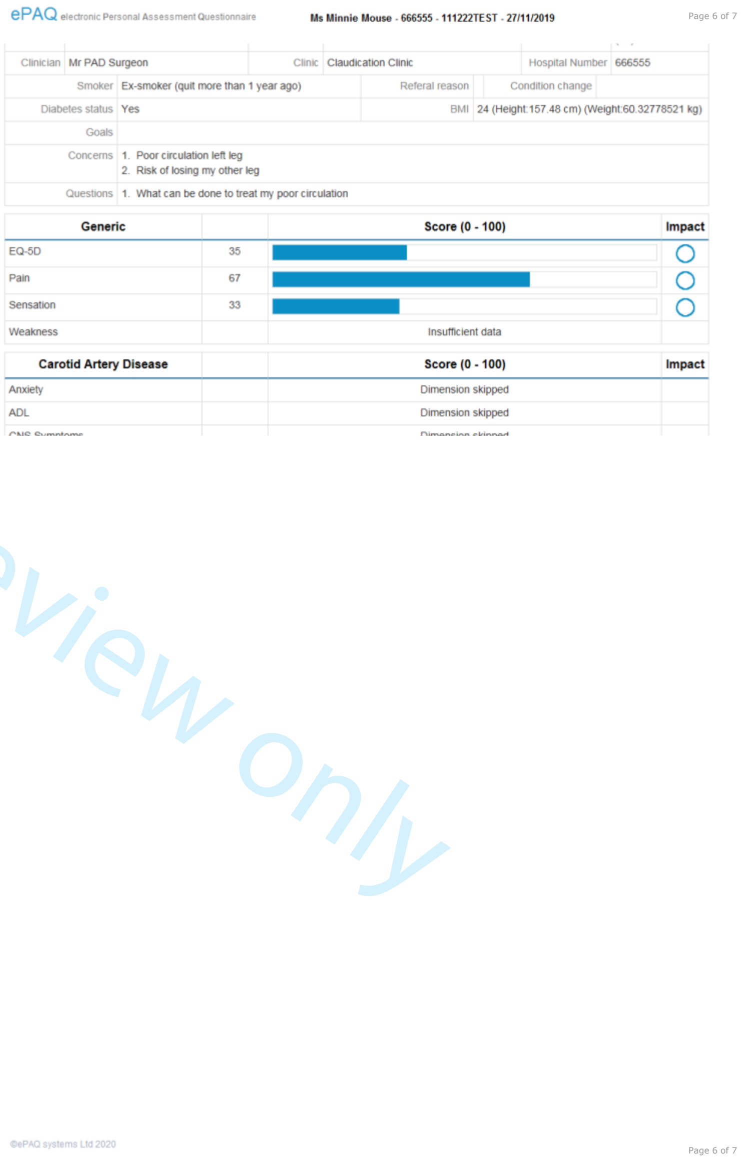

ePAQ electronic Personal Assessment Questionnaire — Ms Minnie Mouse - 666555 - 111222TEST - 27/11/2019 — Page 6 of 7

Clinician	Mr PAD Surgeon	Clinic	Claudication Clinic	Hospital Number	666555
Smoker	Ex-smoker (quit more than 1 year ago)	Referal reason		Condition change	
Diabetes status	Yes	BMI	24 (Height:157.48 cm) (Weight:60.32778521 kg)		
Goals					
Concerns	1. Poor circulation left leg<br>2. Risk of losing my other leg				
Questions	1. What can be done to treat my poor circulation				

Generic		Score (0 - 100)	Impact
EQ-5D	35		
Pain	67		
Sensation	33		
Weakness		Insufficient data	

Carotid Artery Disease		Score (0 - 100)	Impact
Anxiety		Dimension skipped	
ADL		Dimension skipped	

©ePAQ systems Ltd 2020 — Page 6 of 7

ePAQ electronic Personal Assessment Questionnaire **Ms Minnie Mouse - 666555 - 111222TEST - 27/11/2019** Page 7 of 7

Clinician	Mr PAD Surgeon	Clinic	Claudication Clinic	Hospital Number	666555
Smoker	Ex-smoker (quit more than 1 year ago)	Referal reason		Condition change	
Diabetes status	Yes	BMI	24 (Height:157.48 cm) (Weight:60.32778521 kg)		
Goals					
Concerns	1. Poor circulation left leg<br>2. Risk of losing my other leg				
Questions	1. What can be done to treat my poor circulation				

Generic		Score (0 - 100)	Impact
EQ-5D	35		
Pain	67		
Sensation	33		
Weakness		Insufficient data	

Carotid Artery Disease		Score (0 - 100)	Impact
Anxiety		Dimension skipped	
ADL		Dimension skipped	
CNS Symptoms		Dimension skipped	

Aortic aneurysm		Score (0 - 100)	Impact
Quality of Life		Dimension skipped	
Anxiety		Dimension skipped	
AAA Symptoms		Insufficient data	

Lower Limb		Score (0 - 100)	Impact
Pain	0		
Ulceration	0		
Varicose veins		Insufficient data	
ADL	35		
Claudication	62		

©ePAQ systems Ltd 2020

r review only

For peer review only - http://bmjopen.bmj.com/site/about/guidelines.xhtml
